# High-throughput deep sequencing reveals that microRNAs play important roles in salt tolerance of euhalophyte *Salicornia europaea*

**DOI:** 10.1186/s12870-015-0451-3

**Published:** 2015-02-26

**Authors:** Juanjuan Feng, Jinhui Wang, Pengxiang Fan, Weitao Jia, Lingling Nie, Ping Jiang, Xianyang Chen, Sulian Lv, Lichuan Wan, Sandra Chang, Shizhong Li, Yinxin Li

**Affiliations:** Institute of Botany, Key Laboratory of Plant Molecular Physiology, Chinese Academy of Sciences, Beijing, 100093 China; Department of Biochemistry and Molecular Biology, Michigan State University, 603 Wilson road, East Lansing, MI 48824 USA; Beijing Engineering Research Center for Biofuels, Tsinghua University, Beijing, 100084 China; Institute of Nuclear and New Energy Technology, Tsinghua University, Beijing, 100084 China

**Keywords:** *Salicornia europaea*, Euhalophyte, miRNA, Deep sequencing, Salt stress, Lignin biosynthesis, Biofuel crop

## Abstract

**Background:**

microRNAs (miRNAs) are implicated in plant development processes and play pivotal roles in plant adaptation to environmental stresses. *Salicornia europaea*, a salt mash euhalophyte, is a suitable model plant to study salt adaptation mechanisms. *S. europaea* is also a vegetable, forage, and oilseed that can be used for saline land reclamation and biofuel precursor production on marginal lands. Despite its importance, no miRNA has been identified from *S. europaea* thus far.

**Results:**

Deep sequencing was performed to investigate small RNA transcriptome of *S. europaea*. Two hundred and ten conserved miRNAs comprising 51 families and 31 novel miRNAs (including seven miRNA star sequences) belonging to 30 families were identified. About half (13 out of 31) of the novel miRNAs were only detected in salt-treated samples. The expression of 43 conserved and 13 novel miRNAs significantly changed in response to salinity. In addition, 53 conserved and 13 novel miRNAs were differentially expressed between the shoots and roots. Furthermore, 306 and 195 *S. europaea* unigenes were predicted to be targets of 41 conserved and 29 novel miRNA families, respectively. These targets encoded a wide range of proteins, and genes involved in transcription regulation constituted the largest category. Four of these genes encoding laccase, F-box family protein, SAC3/GANP family protein, and NADPH cytochrome P-450 reductase were validated using 5′-RACE.

**Conclusions:**

Our results indicate that specific miRNAs are tightly regulated by salinity in the shoots and/or roots of *S. europaea*, which may play important roles in salt tolerance of this euhalophyte. The *S. europaea* salt-responsive miRNAs and miRNAs that target transcription factors, nucleotide binding site-leucine-rich repeat proteins and enzymes involved in lignin biosynthesis as well as carbon and nitrogen metabolism may be applied in genetic engineering of crops with high stress tolerance, and genetic modification of biofuel crops with high biomass and regulatable lignin biosynthesis.

**Electronic supplementary material:**

The online version of this article (doi:10.1186/s12870-015-0451-3) contains supplementary material, which is available to authorized users.

## Background

microRNAs (miRNAs) are a class of endogenous small non-coding RNAs (sRNAs) that are 21–24 nt in length; they regulate gene expression at transcriptional and post-transcriptional levels [1]. Since their discovery in *Caenorhabditis elegans* in 1993 [2], miRNAs have been extensively detected in plants, animals, and some viruses through direct cloning, bioinformatic prediction, and high-throughput sequencing. In plants, miRNA genes (MIR) are transcribed by RNA polymerase II to form 5′-capped, spliced and 3′-poly (A)-tailed primary transcripts, known as primary-miRNAs (pri-miRNAs). Pri-miRNAs are folded into unique stem-loop structures that are subsequently recognized and processed by Dicer-like 1 (DCL1) enzymes of the RNase III family using two steps: first, into smaller stem-loop structures called precursor-miRNAs (pre-miRNAs), and then into a double-stranded miRNA/miRNA* duplex, usually with 2 nt overhangs on the 3′ end. One of the strands, called mature miRNA, is incorporated into the RNA-induced silencing complex (RISC), whereas the other strand is usually degraded. The incorporated mature miRNA guides the RISC to target mRNA by base pairing, either cleaving the target with near perfect complementarity or repressing its translation with lower complementarity [3].

miRNAs participate in diverse plant growth and development processes, including leaf morphogenesis and polarity, floral differentiation and development, root initiation and development, vascular development, and phase transition [4]. In addition, various studies have demonstrated that miRNAs are involved in plant responses to abiotic and biotic stresses [5,6].

Salinity is one of the most severe and wide-ranging abiotic stresses that adversely affect plant growth and limit the yields of major crops worldwide. Thus far, soil salinity has been an increasing agricultural problem. More than 800 million ha of the world’s land area, which account for over 6% of the land worldwide, are estimated to be affected by salinity (FAO, 2008). Elucidating the mechanisms of plant responses to salinity is an important topic for genetic engineering of crops to improve salt tolerance and ultimately improve crop yield and quality. As sessile organisms, plants have developed various adaptive mechanisms to improve their resistance against salt stress. Over the past decades, numerous studies have focused on revealing the complex mechanisms underlying plant tolerance to salt stress. Salt tolerance is a complex trait controlled by multiple genes, which are strictly regulated at several levels under salinity conditions [7]. In addition to transcriptional factors, miRNAs also play pivotal roles in plant responses to salt stress in many species [8-20].

In contrast to glycophytes, halophytes can thrive in highly saline conditions and are good candidate materials to study salt adaptation mechanisms in plants. The biomass production of halophytes with seawater irrigation varies from 10 ton/ha to 20 ton/ha, which is equivalent to that of conventional crops [21]. Thus, halophytes have been increasingly regarded as a new source of crop that can be used for saline land reclamation and biofuel precursor production. Investigating miRNAs in halophytes, particularly euhalophytes, will help us understand the molecular mechanisms of salt adaptation in plants. Moreover, it will pave the way for further applications in breeding practices and biofuel production in marginal lands. However, research on miRNAs in halophytes is relatively limited compared with that in other plant species. This gap is largely due to the lack of information on their genome or transcriptome sequences and the difficulties in genetic manipulation. Thus far, only two studies identified miRNAs from halophytes including *Thellungiela salsuginea* [9] and *Salicornia brachiata* [20].

*Salicornia europaea*, a salt marsh euhalophyte belonging to Chenopodiaceae, is one of the most salt-tolerant plant species worldwide [22]. *S. europaea* has been recognized as a model plant to study the molecular mechanisms of halophytes in surviving under salinity conditions. *Salicornia* seeds contain high levels of unsaturated oils and proteins [21,23]; hence, they are economically feasible as a feedstock farm crop for biodiesel and other energy products. Moreover, they can be grown on marginal land and typically do not compete with food crops for land resources. Physiological, proteomic, and transcriptome analyses have been applied to illustrate the salt tolerance mechanisms of *S. europaea* [24-28]. However, no investigation into *S. europaea* miRNAs has been reported to date. Taking advantage of *S. europaea* transcriptome data in our previous study [27], we globally analyzed miRNAs in *S. europaea* through high-throughput sequencing technology and bioinformatics analysis in the present study*.* Conserved and novel miRNAs of *S. europaea* were identified, and their targets were predicted. The expression profiles of miRNAs during salt treatment and between the shoots and roots were also investigated. This study contributed in elucidating the molecular mechanisms of salt tolerance in *S. europaea*. Specific miRNAs in *S. europaea* may be applied in breeding stress-tolerant plants and genetically engineering plants with improved properties, which are suitable for growing on marginal lands.

## Results

### Sequencing and data analysis

In the present study, six sRNA libraries were constructed from the shoots and roots of *S. europaea* seedlings treated with 200 mM NaCl for 0 h, 12 h, and 7 d (named S-0 h, R-0 h, S-12 h, R-12 h, S-7 d, and R-7 d, respectively). High-throughput sequencing was then performed to identify *S. europaea* miRNAs responsive to salt stress. Each library generated more than 13 million raw sRNA readouts. After removing low-quality sequences, adaptor contaminants, poly (A) sequences, RNAs smaller than 18 nt, and other artifacts, we obtained about five million unique high-quality sRNAs from each library (Table 1).

**Table 1:** **Statistics of sRNAs (small RNA) sequences from the individual libraries**

**Category**	S-0 h (%*)	R-0 h (%*)	S-12 h (%*)	R-12 h (%*)	S-7 d (%*)	R-7 d (%*)
Total raw reads	13,469,920 (100)	14,055,204 (100)	13,022,329 (100)	15,082,285 (100)	15,458,638 (100)	16,720,995 (100)
High quality reads	13,182,326 (97.865)	13,760,385 (97.902)	12,789,856 (98.215)	14,806,386 (98.171)	15,157,737 (98.054)	16,328,908 (97.655)
3′ adaptor null reads	41,860 (0.311)	41,310 (0.294)	33,666 (0.259)	44,797 (0.297)	46,888 (0.303)	56,998 (0.341)
Insert null reads	3,031 (0.023)	1,505 (0.011)	2,756 (0.021)	2,639 (0.018)	4,029 (0.026)	2,549 (0.015)
5′ adaptor contaminant reads	9,566 (0.071)	8,254 (0.059)	6,932 (0.053)	12,279 (0.081)	8,836 (0.057)	34,253 (0.205)
Smaller than 18 nt reads	84,194 (0.625)	192,261 (1.368)	49,530 (0.380)	60,857 (0.404)	82,752 (0.535)	185,583 (1.110)
Poly(A) sequence reads	269 (0.002)	447 (0.003)	485 (0.004)	1,678 (0.011)	308 (0.002)	812 (0.005)
Total clean sRNA reads	13,043,406 (96.834)	13,516,608 (96.168)	12,696,487 (97.498)	14,684,136 (97.360)	15,014,924 (97.130)	16,048,713 (95.979)
Unique sequence reads	5,692,793 (42.263)	4,919,934 (35.004)	5,245,389 (40.280)	5,250,004 (34.809)	5,624,720 (36.386)	5,139,762 (30.738)
Singleton sequence reads	4,503,775 (33.436)	3,841,747 (27.333)	4,033,830 (30.976)	3,959,027 (26.250)	4,392,276 (28.413)	3,881,932 (23.216)
Unique sequence reads (>2 reads)	1,189,018 (8.827)	1,078,187 (7.671)	1,211,559 (9.304)	1,290,977 (8.560)	1,232,444 (7.973)	1,257,830 (7.523)

* The ratio is equal to the separate reads divided by the total raw reads.

S-0 h, S-12 h, and S-7 d represent shoot treated with 200 mM NaCl for 0 h, 12 h, and 7 d, respectively. R-0 h, R-12 h, and R-7 d represent root treated with 200 mM NaCl for 0 h, 12 h, and 7 d, respectively.

In our previous study, we acquired *S. europaea* mRNA transcriptome sequences that contain 57,151 unigenes longer than 300 bp and 23,585 unigenes longer than 500 bp [27]. In the present study, we mapped these unique sRNA sequences to *S. europaea* transcriptome database by using the computational software SOAP (http://soap.genomics.org.cn) [29]. About 3% unique sRNAs (accounting for 11% to 17% redundant sRNAs) perfectly matched the *S. europaea* mRNA transcriptome sequences. Thereafter, the known non-coding RNAs, including rRNAs, tRNAs, snRNAs, and snoRNAs, were annotated and removed. The remaining sRNA sequences were used for BLASTn search against the known plant miRNAs in the public miRNA database miRBase [30]. The numbers and proportion of different types of small RNAs are shown in Table 2.

**Table 2:** **Annotations of sRNAs perfectly matching** ***S. europaea*** **mRNA transcriptome**

**Class**	**S-0 h (%)**		**R-0 h (%)**		**S-12 h (%)**		**R-12 h (%)**		**S-7 d (%)**		**R-7 d (%)**	
	**Unique**	**redundant**	**Unique**	**redundant**	**Unique**	**redundant**	**Unique**	**redundant**	**Unique**	**redundant**	**Unique**	**redundant**
**Clean reads**	5,692,793	13,043,406	4,919,934	13,516,608	5,245,389	12,696,487	5,250,004	14,684,136	5,624,720	15,014,924	5,139,762	16,048,713
	(100)	(100)	(100)	(100)	(100)	(100)	(100)	(100)	(100)	(100)	(100)	(100)
**Total match**	169,700	1,485,433	153,610	2,387,707	163,343	1,433,469	162,686	1,931,422	167,370	1,648,118	172,627	2,065,163
	(2.981)	(11.388)	(3.122)	(17.665)	(3.114)	(11.290)	(3.100)	(13.153)	(2.976)	(10.976)	(3.359)	(12.868)
**rRNA**	20,751	203,443	52,512	724,501	18,856	179,003	57,172	597,189	30,676	489,063	64,971	734,950
	(0.365)	(1.560)	(1.067)	(5.360)	(0.359)	(1.410)	(1.089)	(4.067)	(0.545)	(3.257)	(1.264)	(4.579)
**tRNA**	15,192	595,658	33,291	566,403	10,033	582,953	28,844	349,209	27,657	1,753,163	59,291	1,088,520
	(0.267)	(4.567)	(0.677)	(4.190)	(0.191)	(4.591)	(0.549)	(2.378)	(0.492)	(11.676)	(1.154)	(6.783)
**snRNA**	615	1,002	1,247	3,815	569	1,031	1,269	3,998	681	1,133	1,982	6,431
	(0.011)	(0.008)	(0.025)	(0.028)	(0.011)	(0.008)	(0.024)	(0.027)	(0.012)	(0.008)	(0.039)	(0.040)
**snoRNA**	305	637	446	1,496	261	560	566	1,779	342	830	1,153	4,322
	(0.005)	(0.005)	(0.0095)	(0.011)	(0.005)	(0.004)	(0.011)	(0.012)	(0.006)	(0.006)	(0.022)	(0.027)
**repeat**	2,072	36,665	5,019	440,957	2,016	16,666	5,891	41,192	2,338	222,854	4,953	80,746
	(0.036)	(0.281)	(0.102)	(3.262)	(0.038)	(0.131)	(0.112)	(0.281)	(0.042)	(1.484)	(0.096)	(0.503)
**miRNA**	19,973	559,972	20,531	796,548	15,457	482,091	20,198	912,186	23,822	600,269	21,115	1,047,498
	(0.351)	(4.293)	(0.417)	(5.893)	(0.295)	(3.797)	(0.385)	(6.212)	(0.424)	(3.998)	(0.411)	(6.527)
**siRNA**	189,944	1,403,603	135,787	1,571,363	206,456	1,359,560	152,158	1,348,629	180,530	1,219,863	134,728	1,255,102
	(3.337)	(10.761)	(2.760)	(11.625)	(3.936)	(10.708)	(2.898)	(9.184)	(3.209582)	(8.124)	(2.621)	(7.821)
**Un-anno-tated**	5,446,013	10,279,091	4,676,120	9,852,482	4,993,757	10,091,289	4,989,797	11,471,146	5,361,012	10,950,603	4,856,522	11,911,890
	(95.66505)	(78.807)	(95.044)	(72.892)	(95.203)	(79.481)	(95.044)	(78.119)	(95.31162)	(72.931)	(94.489)	(74.223)

S-0 h, S-12 h, and S-7 d represent shoot treated with 200 mM NaCl for 0 h, 12 h, and 7 d, respectively. R-0 h, R-12 h, and R-7 d represent root treated with 200 mM NaCl for 0 h, 12 h, and 7 d, respectively.

Although some sRNAs were abundant and appeared hundreds of thousands times in our database, the majority of sRNAs were only sequenced few times. For example, 4,503,775 (79%) sRNAs were sequenced only once in S-0 h (Table 1), indicating that *S. europaea* contained a large and complex sRNA population. The sRNA singleton rate of *S. europaea* (77% in average) is similar to that of *Arabidopsis thaliana* (65%), *Oryza sativa* (82%), and *Cunninghamia lanceolata* (74%) [31].

**Figure 1:**
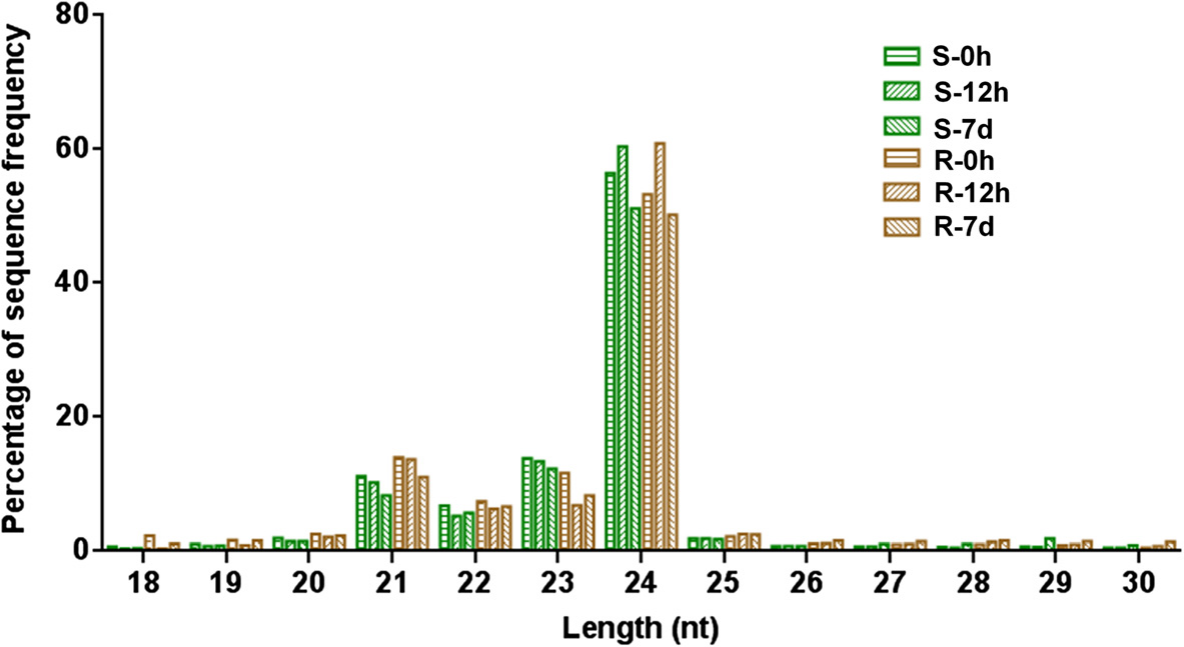
**Length distribution of small RNAs in different libraries.** nt, nucleotides. S-0 h, S-12 h, and S-7 d represent the shoots treated with 200 mM NaCl for 0 h, 12 h, and 7 d, respectively. R-0 h, R-12 h, and R-7 d denote the roots treated with 200 mM NaCl for 0 h, 12 h, and 7 d, respectively.

The majority of total sRNA reads ranged from 20 nt to 24 nt in length, which are the typical size range for Dicer-derived products [32]. For all six libraries, 24 nt sRNAs were the most abundant, which is consistent with the typical small RNA distribution patterns in angiosperms. However, the proportion of 24 nt sRNAs dynamically changed under salinity conditions; these sRNAs increased during short-term salt treatment but decreased during long-term treatment (Figure 1). The second most abundant class was 23 nt in the shoots and 21 nt in the roots (Figure 1); nevertheless, the differences in their proportions should be further clarified.

### Identification of conserved miRNAs

**Figure 2:**
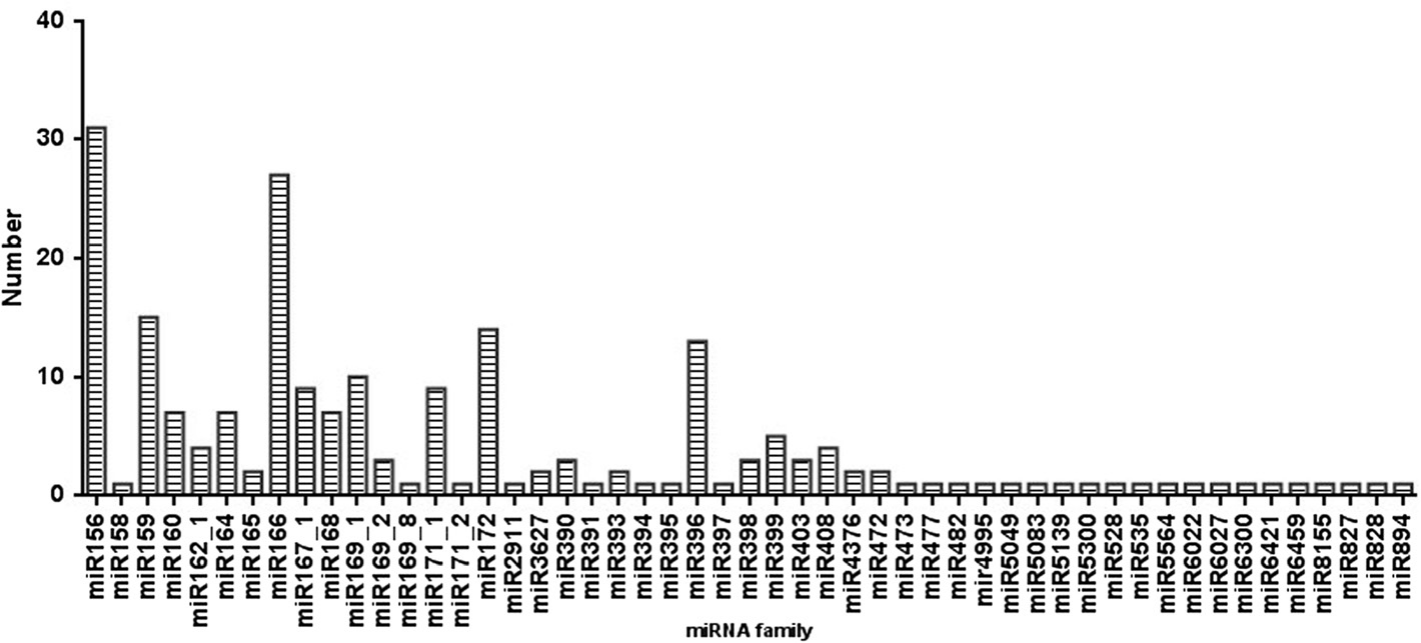
**Number of conserved miRNAs in each family in** ***S. europaea.***

Thus far, no *S. europaea* miRNAs have been reported. In this study, we conducted a local BLASTn search using *S. europaea* unique sRNA candidates against all plant miRNAs in the miRBase (Release 20.0, June 2013); this database contains 7,385 miRNAs across 72 plant species [30]. For precursor prediction, we used the transcriptome sequences of *S. europaea* [27] to determine inverted repeats and stem-loop structures. Only sequences that perfectly matched with known plant miRNA sequences or with stem-loop precursors were considered conserved miRNAs. Finally, 210 conserved mature miRNAs belonging to 51 miRNA families were identified in *S. europaea* (Additional file 1). The length varied from 18 nt to 23 nt, and 21 nt and 20 nt miRNAs were the two major size classes (Additional file 2). Notably, 148 (69.8%) conserved miRNAs started with a 5′ terminal uridine residue, a feature of miRNAs recognized by the AGO1 protein [33]. Moreover, the number of members within the miRNA family considerably differed. For example, seu-miR166 and seu-miR156 families contained 26 and 31 members, respectively; in addition, many miRNA families (e.g., seu-miR158, seu-miR394, and seu-miR395) comprised only one member. A total of 23 conserved miRNA families contained more than one member. The members of each family are summarized in Figure 2.

We identified 10 conserved miRNA precursors with lengths ranging from 92 nt to 252 nt. Their minimal folding free energy indices (MFEIs) varied from 0.45 to 1.02 with an average value of 0.85 (Additional file 1). These parameters are similar to those of other plant miRNAs, such as *A. thaliana*, *O. sativa*, *Glycine max*, *Medicago truncatula*, and *C. lanceolata* [31]. Only five conserved miRNAs contained star sequences, and three of them (seu-miR164a, seu-miR166a, and seu-miR172b) belonged to high confidence sequences according to the criterion of miRBase.

We also conducted cloning experiments to validate the pre-miRNA sequences, and six of these sequences, namely, pre-miR319a, pre-miR164a, pre-miR166a, pre-miR168a, pre-miR398a, and pre-miR399d, were confirmed. Three validated sequences contained only one mismatched nucleotide compared with the sequences obtained from Illumina sequencing. Moreover, two sequences contained less than six mismatched nucleotides and one sequence comprised more than six mismatched nucleotides. This discrepancy may be partially attributed to the sequence assembly errors during Illumina sequencing (Additional file 1). The primary, precursor sequences, and hairpin structures of *S. europaea* conserved miRNAs predicted by MFOLD are shown in Additional files 3 and 4.

### Identification of novel miRNAs in *S. europaea*

We also identified 31 putative novel miRNAs belonging to 30 families in *S. europaea* and named them as seu-miR1 to seu-miR30. Among these miRNAs, seu-miR10a and seu-miR10b shared similar mature sequence and therefore were classified into one family (Additional file 5). When we deposited these new miRNAs to miRBase, we found seu-miR14 was homologous to ata-miR319, a new added conserved miRNA in Release 21, with two mismatched nucleotides. Thus it was renamed seu-miR319. The other novel miRNAs were assigned names of seu-miR11021 to seu-miR11051 by miRBase, respectively (Additional file 5). The length of miRNA precursors specific to *S. europaea* ranged from 64 nt to 272 nt, and MFEIs varied from 0.49 to 1.88 with an average value of 0.85 (Additional files 5, 6 and 7). Seven miRNA star sequences were identified from the six sRNA libraries, confirming their identity as novel miRNAs. However, the star sequences for the remaining novel miRNAs were not detected, which could be due to their low expression or poor stability. Eighteen (58%) mature sequences of the novel miRNAs started with a 5′ terminal uridine residue. The length of *S. europaea* novel miRNAs varied from 19 nt to 23 nt, and 21 nt was the major class size (Additional file 8).

Most novel miRNAs showed unique expression patterns. Four of these miRNAs, namely, seu-miR3 to seu-miR6, were only expressed in *S. europaea* roots, whereas seu-miR7 was only detected in the shoots. Thirteen novel miRNAs (seu-miR8 and seu-miR19 to seu-miR30) were uniquely expressed in salt-treated shoots or roots. Furthermore, the expression levels of these novel miRNAs were relatively low (Additional file 5), which is a feature of species-specific miRNAs.

For validation, the precursor sequences of 15 novel miRNAs were cloned. Three of these sequences (seu-miR8, 14, and 29) were identical to the sequences obtained from Illumina sequencing. Ten of these novel miRNAs contained less than six mismatched nucleotides, and three had more than six mismatched nucleotides (Additional file 5). This finding may be partially attributed to sequence assembly mistakes during Illumina sequencing.

### Expression profiles of conserved and novel miRNAs

**Figure 3:**
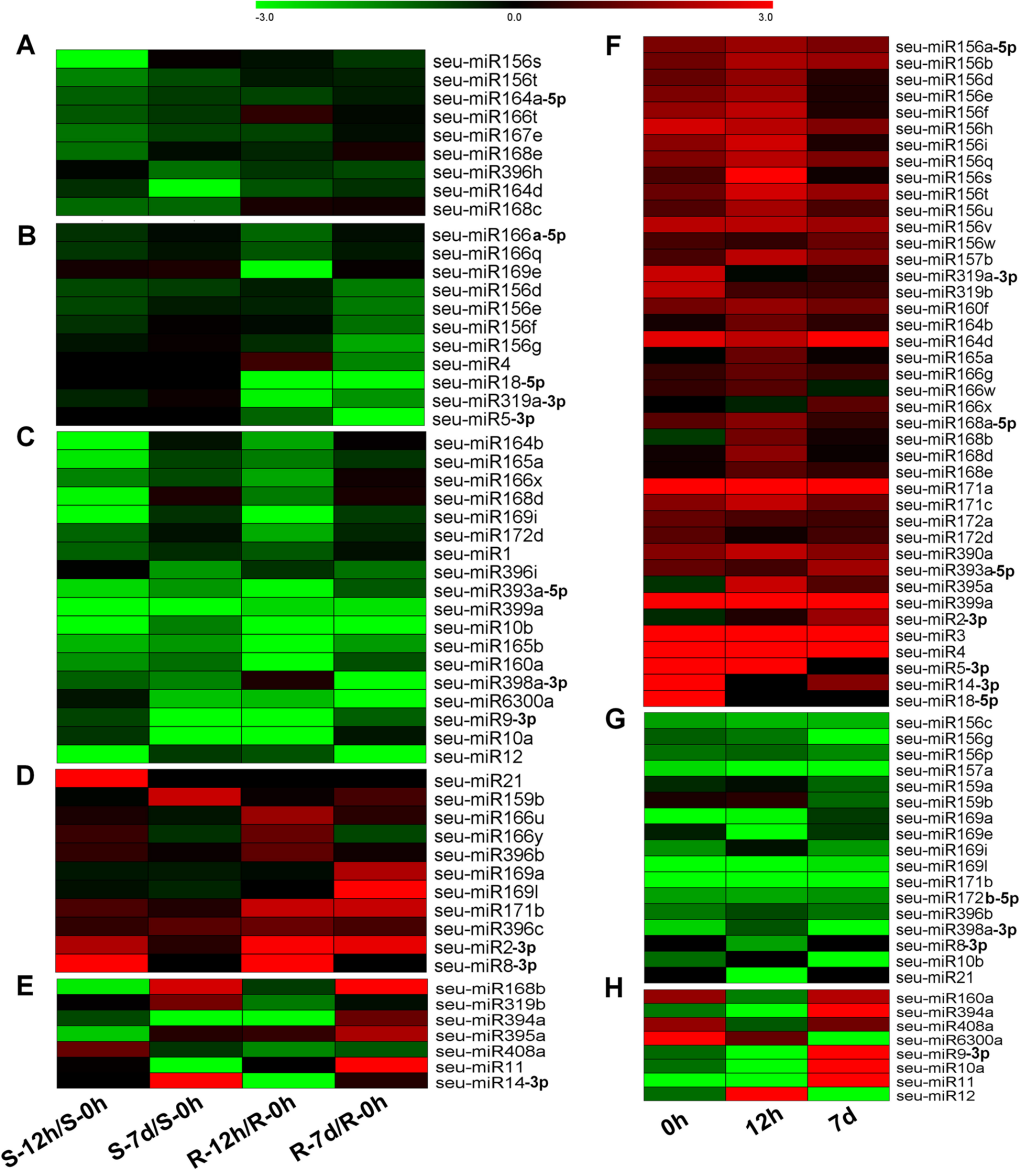
**Differentially expressed** ***S. europaea*** **miRNAs after salt treatment or between the shoots and roots. A** to **E**, Heat map of differentially expressed *S. europaea* miRNAs after salt treatment**.** These miRNAs were divided into five categories based on their expression patterns. **(A)** miRNAs down-regulated in salt-treated shoots. **(B)** miRNAs down-regulated in salt-treated roots. **(C)** miRNAs down-regulated in salt-treated shoots and roots. **(D)** miRNAs up-regulated by salt. **(E)** miRNAs dynamically regulated during salt treatment. Relative expression level was calculated using Log_2_(RPM_Salt_/RPM_salt-0 h_). **F** to **H**, Heat map of differentially expressed *S. europaea* miRNAs between the shoots and roots. **(F)** miRNAs expressed higher in the roots than those in the shoots. **(G)** miRNAs expressed higher in the shoots than those in in the roots. **(H)** miRNAs distributed dynamically between the shoots and roots during salt treatment. Relative expression level was calculated with Log_2_(RPM_root_/RPM_shoot_). S-0 h, S-12 h, and S-7 d represent the shoots treated with 200 mM NaCl for 0 h, 12 h, and 7 d, respectively. R-0 h, R-12 h, and R-7 d denote the roots treated with 200 mM NaCl for 0 h, 12 h, and 7 d, respectively.

To detect the effect of salinity on *S. europaea* miRNA expression, we performed a differential expression analysis between the libraries treated and non-treated with salt. All miRNAs with more than one normalized reads were analyzed by calculating fold changes and *P* value. miRNAs with *P* values lower than 0.05 and fold changes higher than 2 were considered significantly altered. A total of 43 conserved miRNAs (belonging to 19 families) and 13 novel miRNAs (belonging to 12 families) significantly changed in response to salt treatment in *S. europaea* (Additional file 9, sheet 1). These miRNAs were divided into five categories based on their expression patterns (Figure 3A to E).

The first category contained miRNAs with expression levels that were down-regulated in salt-treated *S. europaea* shoots. Specifically, miR156s/t, miR164a-5p, miR166t, miR167e, and miR168e were significantly suppressed in S-12 h, whereas miR396h and miR164d were suppressed in S-7 d. Additionally, miR168c was down-regulated in S-12 h and S-7 d (Figure 3A).

As shown in Figure 3B, the second category comprised miRNAs exclusively down-regulated in salt-treated *S. europaea* roots. The expression of miR166a-5p, miR166q, and miR169e was down-regulated in R-12 h, whereas that of miR156d/e/f/g and miR4 was down-regulated in R-7 d. miR18-5p, miR319a-3p, and miR5-3p were suppressed in R-12 h and R-7 d (Figure 3B).

miRNAs in the third category were down-regulated in the shoots and roots after salt treatment. miR164b, miR165a, miR166x, miR168d, miR169i, miR172d, and miR1 changed only after 12 h of salt treatment. By contrast, miR396i was repressed after 7 d of salt treatment. Furthermore, the expression of miR393a-5p, miR399a, miR10b, miR165b, miR160a, miR398a-3p, miR6300a, miR9-3p, miR10a, and miR12 was down-regulated in the shoots and roots treated with salt for 12 h and 7 d (Figure 3C).

The fourth group contained 11 up-regulated miRNAs. The expression of miR21 was up-regulated in S-12 h, and miR159b was up-regulated in S-7 d. Moreover, miR166u/y and miR396b were induced in R-12 h, whereas miR169a and miR169l were induced in R-7 d. The expression of miR171b, miR396c, miR2-3p, and miR8-3p was up-regulated by salt in the shoots and roots (Figure 3D).

miRNAs in the last group were dynamically regulated in response to salt stress. For example, miR394a was suppressed in the roots after 12 h of salt treatment and then induced in the roots but suppressed in the shoots after 7 d of salt treatment. The expression of miR168b, miR319b, miR395a, miR408a, miR11, and miR14-3p also demonstrated similar pattern of dynamic changes in the shoots and roots during salt treatment (Figure 3E).

We also compared the expression of miRNAs between the shoots and roots. We detected 66 differentially expressed miRNAs from 19 conserved and 12 novel miRNA families (Figure 3F to H; Additional file 9, sheet 2). The expression of more than half (42, 64%) of these miRNAs was higher in the roots (Figure 3F), whereas 17 miRNAs were higher in the shoots (Figure 3G). In addition, seven miRNAs were dynamically distributed between the shoots and roots during salt treatment (Figure 3H).

**Figure 4:**
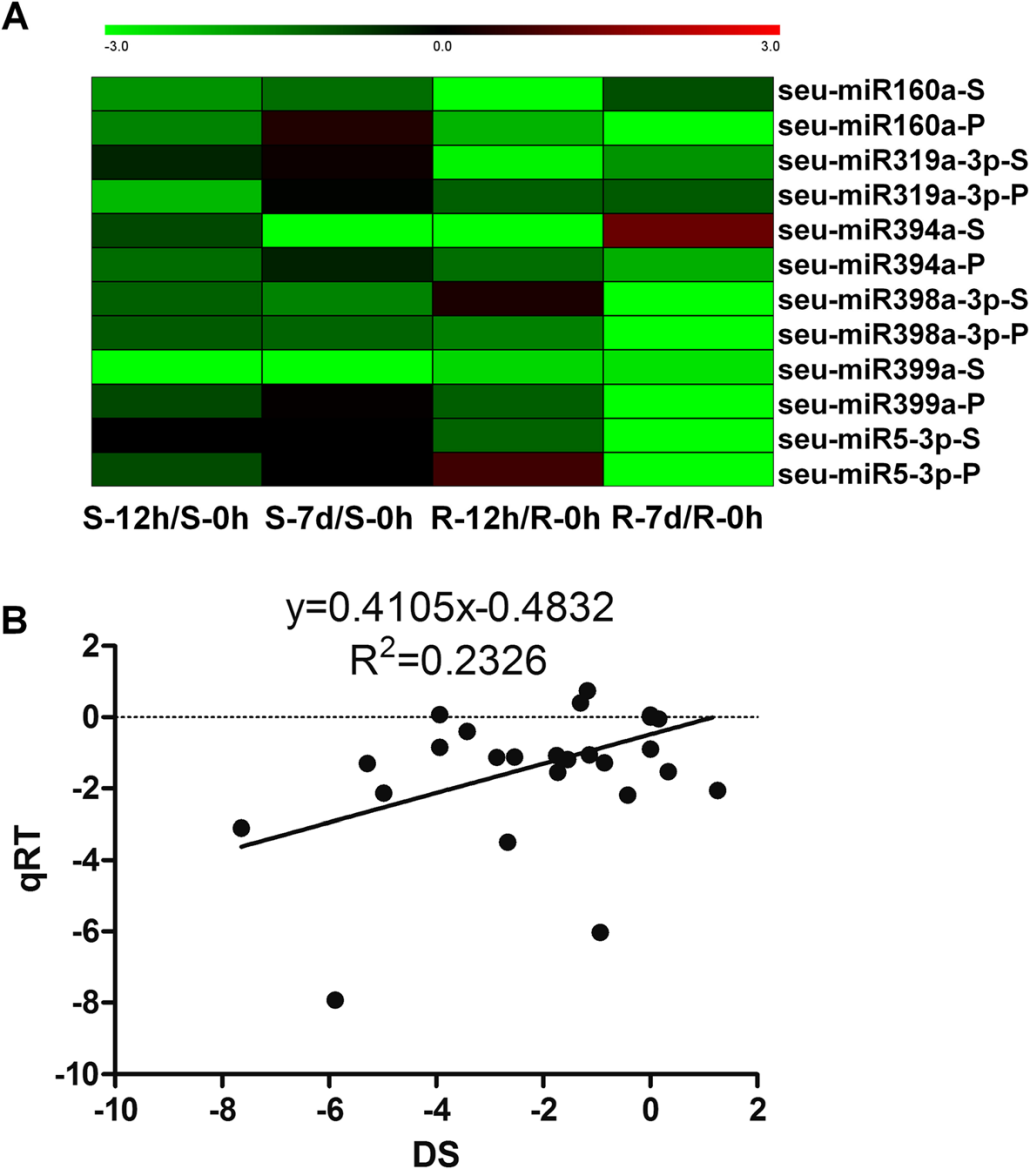
**Validation of expression profiles of miRNAs. (A)** Heat map of sequencing (S) and stem-loop qRT-PCR data (P). Relative expression level was calculated using Log_2_(Salt/Salt-0 h), and qRT-PCR data were averaged using the results from three technical repeats to represent three independent experiments. **(B)** Scatterplot of miRNA expression showing the correlation between deep sequencing (DS) and qRT-PCR (qRT) results. S-0 h, S-12 h, and S-7 d represent the shoots treated with 200 mM NaCl for 0 h, 12 h, and 7 d, respectively. R-0 h, R-12 h, and R-7 d denote the roots treated with 200 mM NaCl for 0 h, 12 h, and 7 d, respectively.

To confirm the expression of identified miRNAs and detect their dynamic responses to salt stress, we selected five conserved miRNAs (miR160a, miR319a-3p, miR394a, miR398a-3p, and miR399a) and one candidate novel miRNA (miR5-3p); we analyzed these miRNAs by using stem-loop qRT-PCR, which is a specific, sensitive, accurate, and reliable method to measure individual miRNAs [34]. Almost all tested miRNAs treated with salt showed similar tendency in deep sequencing and qRT-PCR data compared with the samples not treated with salt (Figure 4A). Furthermore, these miRNAs exhibited positive correlation between the two methods (*R*^2^ = 0.2326, *P* < 0.01), indicating the reliability of the high-throughput data (Figure 4B).

### Target gene prediction of conserved and novel miRNAs

**Figure 5:**
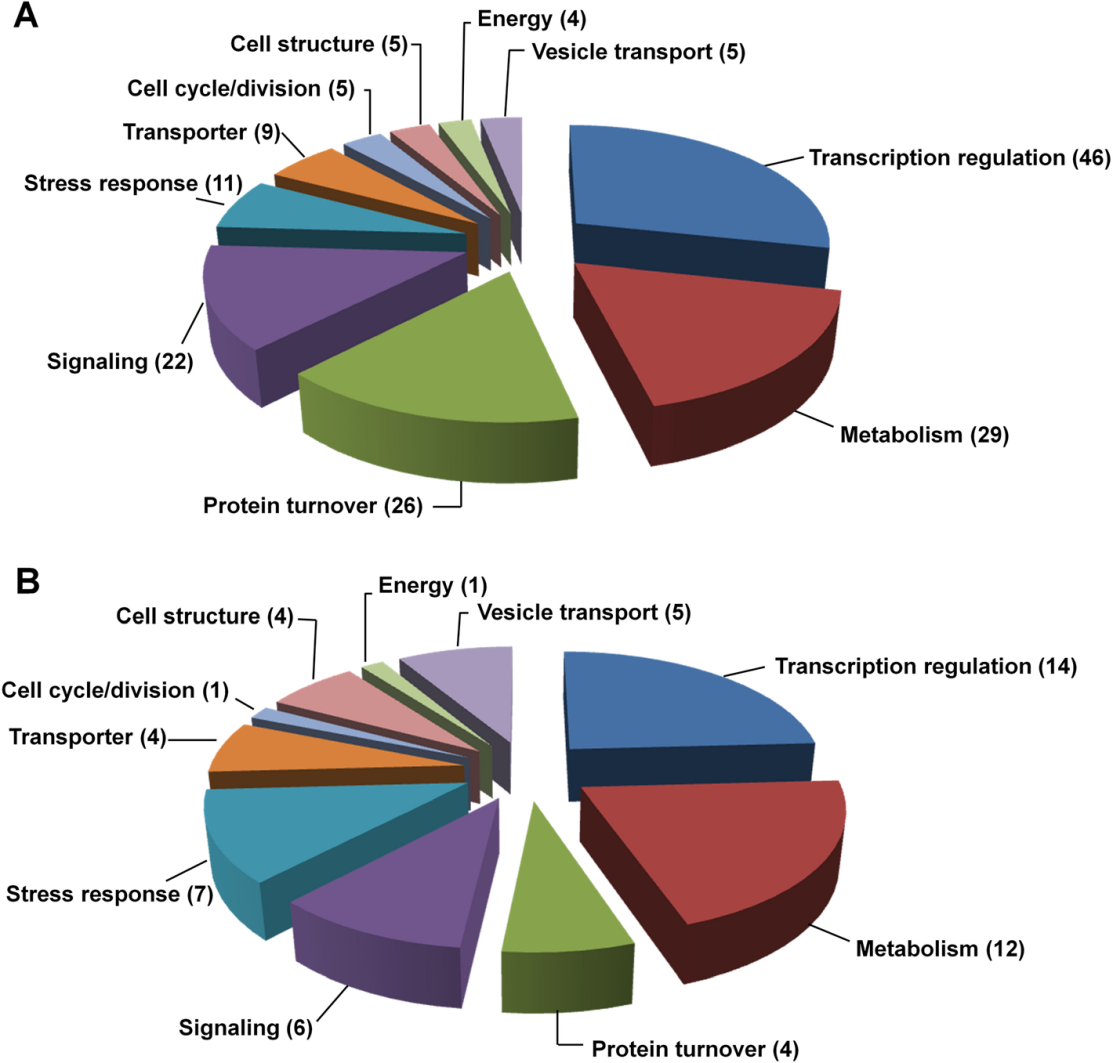
**Functional classification of the target genes of conserved and novel miRNAs in** ***S. europaea*** **.** Only the annotated target genes are shown. **(A)** Annotated target genes of conserved miRNAs. **(B)** Annotated target genes of novel miRNAs. The numbers of target genes are shown in the bracket.

To elucidate the functions of conserved and novel miRNAs of *S. europaea*, we predicted putative targets by using web-based psRNATarget program with default settings (http://plantgrn.noble.org/psRNATarget/?function=3) [35]. A total of 57,151 unigenes from *S. europaea* mRNA transcriptome database were used as a custom target database, whereas 210 conserved and 31 novel mature miRNAs were used as a custom miRNA database. A total of 306 *S. europaea* unigene sequences were predicted as putative targets of 41 conserved miRNA families (Additional file 10, sheet 1). Sixteen unigenes (5.2%) were homologous to the previously confirmed or predicted targets of the same miRNA families in *A. thaliana* and/or *O. sativa* (Table 3). Four miRNA families (seu-miR156, seu-miR160, seu-miR396, and seu-miR397) contained two conserved targets, whereas seven families (seu-miR159, seu-miR164, seu-miR169, seu-miR171, seu-miR394, seu-miR399, and seu-miR403) presented only one conserved target. Most of these conserved targets (10 out of 16) encoded essential transcription factors, and the remaining targets included ABC transporter, F-box protein, proteins involved in sRNA biogenesis and function, and two laccases functioning in lignin biosynthesis. In addition, 290 putative targets of conserved miRNAs were not conserved in other plant species. Among these targets, 144 (47.1%) targets exhibited no functional annotation. The annotated 162 genes participated in a broad spectrum of plant development and physiological processes; these genes were classified into 10 categories based on their molecular and biological functions (Figure 5A). Genes involved in transcriptional regulation (46, 15%; including 16 transcription factors) comprised the major category, followed by unigenes involved in metabolism (29, 9.5%), protein turnover (26, 8.5%), and signaling (22, 7.2%). In addition, many of the target genes identified were directly or indirectly involved in stress responses (11, 3.6%). The remaining categories included transporters (9, 2.9%), genes involved in cell cycle (5, 1.6%), cell structure (5, 1.6%), energy metabolism (4, 1.3%), and vesicle transport (5, 1.6%). Similarly, 195 unigene sequences were predicted to be targets of 29 novel miRNAs (Figure 5B). Most of these sequences (137, 70.3%) have not been functionally annotated (Additional file 10, sheet 2). The functions of the remaining annotated genes were versatile, and genes involved in transcription regulation (14, 7.2%) and metabolism (12, 6.15%) accounted for the two largest proportions. The remaining categories contained comparable genes.

**Table 3:** **Conserved miRNA targets and their putative functions**

**miRNA family**	**Target genes**	**Target gene function**	**Conserved with** ^**a**^	
			**ath**	**osa**
seu-miR156	Unigene3854	SBP-domain protein	+	+
	Unigene48452	SBP-domain protein	+	+
seu-miR159/319	Unigene43114	TCP transcription factor	+	+
seu-miR160	Unigene15333	Auxin response factor	+	+
	Unigene36580	Auxin response factor	+	+
seu-miR162	Unigene15364	Argonaute and Dicer protein		+
seu-miR164	Unigene18186	NAC transcription factor	+	+
seu-miR169	Unigene51126	NF-YA	+	+
seu-miR171	Unigene31557	Scarecrow-like protein 15	+	+
seu-miR394	Unigene56779	F-box protein	+	+
seu-miR396	Unigene14465	Growth-regulating factor	+	+
	Unigene6660	Growth-regulating factor	+	+
seu-miR397	Unigene16755	Laccase	+	+
	Unigene53694	Laccase	+	+
seu-miR399	Unigene7095	ABC transporter	+	+
seu-miR403	Unigene15373	AGO5	+	

^a^ ath and osa represent abbreviations for *A. thaliana* and *O. sativa*.

We were unable to predict the targets for 10 conserved and one novel miRNA families because of insufficient *S. europaea* mRNA sequences.

### Validation of miRNA-guided cleavage of mRNAs

**Figure 6:**
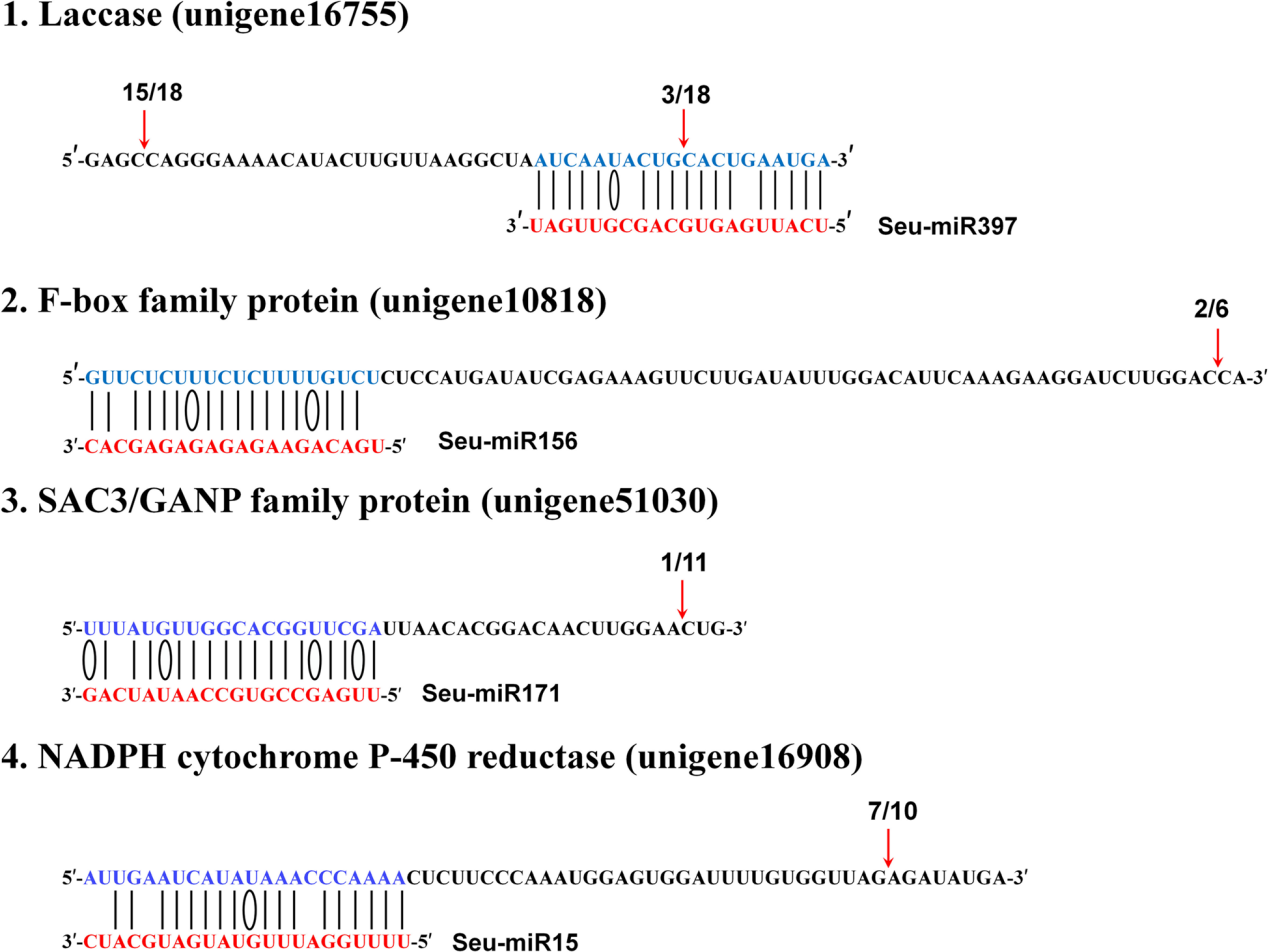
**Verification of miRNA-mediated target gene cleavage through 5′-RACE.** For each miRNA, the partial sequence of the target unigene is shown at the top (blue) and the miRNA sequence at the bottom (red). The perfectly complementary bases are connected by solid lines, G:U wobble pairings are represented by ellipse, and the cleavage sites are shown by the arrows. Numbers indicate the fraction of cloned PCR products that were terminated at the cleavage site.

To validate that miRNAs can regulate their target mRNA expression in *S. europaea*, we amplified the predicted target genes through rapid amplification of 5′ cDNA ends (5′-RACE). Four unigene sequences were verified to be targets of four *S. europaea* miRNAs (Figure 6). Unigene16755, unigene10818, unigene51030, and unigene16908 were confirmed to be targets of seu-miR397, seu-miR156, seu-miR171, and seu-miR15, respectively. Sequencing of the miR397-cleaved 5′ product of unigene16755 revealed a precise slice between the 10th and 11th nucleotide of seu-miR397 from the 5′-end. A shorter or longer cleaved sequence was observed for three putative targets, including unigene10818, unigene51030, and unigene16908, after 5′-RACE analysis. This finding could be attributed to secondary siRNA in the 21-nt register with the cleavage site for miRNAs as previously reported [36]. Unigene16755, unigene10818, unigene51030, and unigene16908 encoded the proteins homologous to laccase, F-box family protein, SAC3/GANP family protein, and NADPH cytochrome P-450 reductase, respectively.

## Discussion

### The salt-responsive miRNAs in *S. europaea*

In our previous studies, 200–400 mM NaCl was found to be necessary for optimal growth of *S. europaea* [26,28], which significantly promotes shoot growth and increases fresh weight, water content, and sodium element content of the aerial parts of the plant [24]. The aim of this study is to investigate the roles of miRNAs with regard to the salt tolerance of *S. europaea*. Usually, 150–300 mM NaCl was used to identify plant salt-responsive miRNAs while 200 mM NaCl treatment has been reported in maize [17], *P. euphratica* [16], *P. tomentosa* [14], *C. intermedia* [8], and *T. salsuginea* [9]. In this study, 200 mM NaCl was selected for salt treatment in order to better compare salt-responsive miRNAs between *S. europaea* and other plants. Several sets of conserved and novel miRNAs in *S. europaea* were differentially expressed in response to salt. More than half of the significantly changed miRNAs (38 out of 56) were down-regulated under salinity conditions (Figure 3, Additional file 9), which is consistent with the result in *T. salsuginea* [9] but in contrast to that in Arabidopsis [37]. The expressed sequence tag analyses revealed 90% to 95% identities between Arabidopsis and its related halophyte Thellungiella [38,39]. Previous studies have shown that the coding sequences, such as SOS1, of many essential components of plant salt tolerance are highly conserved between Thellungiella and Arabidopsis, whereas the promoter region and transcription regulation of these genes differ [40]. As miRNAs serve important functions in the regulation of gene expression, the overall up- or down-regulation trends of miRNAs under salinity in *S. europaea* and Arabidopsis may represent the different salt-responsive mechanisms in halophytes and glycophytes.

### Roles of miRNAs in salt tolerance of *S. europaea*

**Figure 7:**
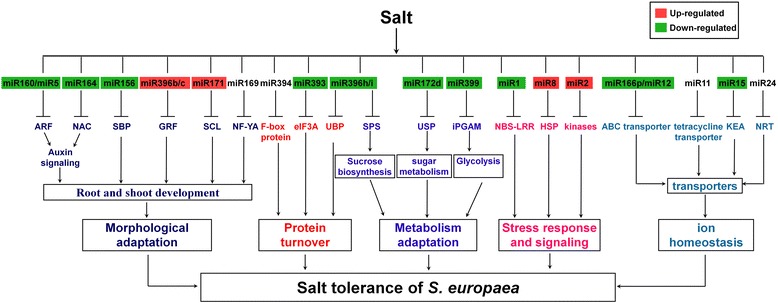
**Potential regulatory network of salt-responsive miRNAs in** ***S. europaea*** **.** Up- and down-regulated miRNAs are highlighted in red and green, respectively. MiR394 and miR11 were dynamically regulated, whereas the members of miR169 differentially changed. In addition, miR24 was only detected in *S. europaea* shoots treated with salt for 7 d.

A potential regulatory network of salt-responsive miRNAs in *S. europaea* is proposed based on the characteristics of their targets (Figure 7, Additional file 11). First, some of these miRNAs target transcription factors involved in the regulation of gene expression and signal transduction, and thus, probably function in salt stress response. Arabidopsis miR156 and its target SQUAMOSA promoter-binding protein-like (SPL) proteins are involved in phase changes, leaf trichome development, male fertility, embryonic patterning, anthocyanin biosynthesis, and plant responses to salt stress [41-45]. miR156 is up-regulated by salt in Arabidopsis [38] and *C. intermedia* but down-regulated in *T. salsuginea* and maize roots [8,9,17]. In the present study, seu-miR156s/t and seu-miR156d/e/f/g were suppressed in S-12 h and R-7 d, respectively (Figure 3A and B). Two SPL proteins were predicted to be targets of seu-miR156 (Additional file 10), implying that miR156 could play important roles in the root development of halophytes under salt conditions by regulating SPL. Previous studies have shown that miR160 can target auxin responsive factors (ARFs) [46,47], and miR164 negatively regulates the expression of NAC (NAM, ATAF1/2, and CUC2) transcription factors [48,49]; both of these miRNAs are involved in auxin signaling. Under salinity conditions, miR160 is induced in *T. salsuginea* but repressed in *O. sativa*, whereas miR164 is down-regulated in *T. salsuginea* and *Z. mays* [17,19,37]. In the present research, we predicted that seu-miR160 targeted two ARF genes, seu-miR5 targeted one ARF gene, and seu-miR164 targeted one NAC transcription factor (Additional file 10). All of these three miRNAs were down-regulated in *S. europaea* after salt treatment (Figure 3A and C), indicating that the release of miRNA-mediated repression of auxin signaling by salt may represent an important mechanism in *S. europaea.* miR169 is up-regulated by salt in Arabidopsis and rice but down-regulated in *T. salsuginea* [9,37,50]. The target gene of miR169 encodes nuclear factor Y subunit A (NF-YA), which is involved in root development, flowering time, nitrogen-starvation responses, and plant responses to drought and salt stresses [50-53]. In *S. europaea*, seu-miR169e/i was strongly down-regulated, whereas seu-miR169a/l was up-regulated after salt treatment (Figure 3B to D). NF-YA, which is the predicted target of seu-miR169, was up-regulated in R-3 h but repressed in R-3 d [27]. This finding confirms that miR169 is strictly regulated by salt and may contribute to the fine-tuning of NF-YA, a critical positive regulator of salt stress tolerance in *S. europaea*. The other three conserved miRNAs, namely, seu-miR171b and seu-miR396b/c, showed different trends in response to salt treatment between *S. europaea* and *T. salsuginea*; all of these miRNAs were induced by salinity in *S. europaea*. Two conserved scarecrow-like (SCL) and two growth response factor (GRF) proteins were predicted to be their targets. Gene regulation under salt stress is mediated by multiple transcriptional cascades, in which a transcription factor gene is induced to activate or repress downstream targets important for salt resistance. These *S. europaea* miRNAs may define several of these cascades, in which they act as crucial molecules at an upstream mode in the transcriptional regulatory networks of salt stress signal transduction; as a result, the morphological adaptation of *S. europaea* can be achieved under salt conditions*.*

Second, some salt-responsive miRNAs target genes involved in protein turnover processes. Seu-miR396h/I, which targets ubiquitin-specific protease (UBPs) that functions in protein degradation, was suppressed during long-term salt treatment of *S. europaea*. Previous studies have shown that UBP16 is required for salt tolerance in Arabidopsis by modulating Na^+^/H^+^ antiporter activity and serine hydroxymethyltransferase1 (SHM1) stability and activity [54]. The suppression of seu-miR396h/i by long-term salt treatment may result in increased expression of UBP; this increased expression serves pivotal functions in salt tolerance of *S. europaea*. In Arabidopsis, miR393, which targets F-box proteins and a basic-helix-loop-helix family protein, is strongly up-regulated by 300 mM NaCl [13]. However, in the present study, the expression of seu-miR393a was down-regulated in the roots and shoots treated with salt for 12 h and 7 d (Figure 3C); this finding is consistent with *T. salsuginea* [9] and *P. tomentosa* [14]. Analysis on transgenic rice and Arabidopsis plants that overexpressed *OsmiR393* found that they are more sensitive to salinity and alkaline stresses [55]. Considering our results and those of previous studies, we speculate that miR393 is a negative regulator of plant salt tolerance.

Third, some other salt-regulated miRNAs target genes encoding metabolic enzymes. One target of seu-miR396h/i encodes sucrose phosphate synthase (SPS), which plays a major role in sucrose biosynthesis. SPS is activated in leaf tissues under osmotic stress in darkness, and thus may function to facilitate sucrose formation for osmoregulation [56]. Moreover, one gene encoding 2, 3-biphosphoglycerate-independent phosphoglycerate mutase (iPGAM) was the target of seu-miR399a (Additional file 10). As a key enzyme involved in glycolysis, iPGAM catalyzes the reversible interconversion of 3-phophoglycerate to 2-phosphoglycerate. In the leaves of the facultative halophyte *Mesembryanthemum crystallinum* (ice plant), iPGAM is induced under saline and drought conditions [57]. In addition, iPGAM also exhibits critical functions in stomatal movement, vegetative growth, and pollen production in Arabidopsis [58]. The regulation of iPGAM by seu-miR399 may contribute to the maintenance of efficient carbon flux through glycolysis/gluconeogenesis under salt conditions in *S. europaea*.

Fourth, specific targets of salt-responsive *S. europaea* miRNAs are related to direct responses to stresses and the downstream signaling processes. These miRNAs include seu-miR1, which could target glyoxal oxidase, Rca6, and nucleotide binding site–leucine-rich repeat (NBS-LRR)-type resistance protein; these proteins are involved in stress and/or disease response. Seu-miR2 could also target leucine-rich repeat family protein and phosphoinositide 3-kinase, which are implicated in the signaling pathway.

Finally, many transporters or ion channels are predicted to be targets of specifically conserved and *S. europaea*-specific miRNAs; these miRNAs may play key roles in ion homeostasis or accumulation of compatible molecules under salt conditions. A potassium efflux antiporter was the candidate target of seu-miR15, which was only detected in control samples and R-7 d. The genes encoding putative K^+^ efflux transporter (KEA) from higher plants were first classified in *A. thaliana*. Thus far, plant KEA function has not been reported despite the importance of its homologs in *E. coli* for bacterial survival during exposure to toxic metabolites [59]. Arabidopsis KEA2 (*AtKEA2*) is highly expressed in the leaves, stems, and flowers, but not in the roots. *AtKEA2* functions as K^+^/H^+^ antiporter and can modulate monovalent cations and pH homeostasis in plant chloroplast or plastids [60]. Therefore, seu-miR15-targeting K^+^ antiporter may play important roles in maintenance of Na^+^ and K^+^ homeostasis in *S. europaea*, which is essential for plant survival under saline conditions. A nitrate transporter was predicted to be the target of seu-miR24, which was only detected in S-7 d. Nitrate is an essential nutrient required for plant growth and also acts as a signal that regulates plant development. Nitrate uptake and its distribution at the whole plant level depend on the combined activity of nitrate transporters; a sodium/nitrate cotransport system has been reported in some halotolerant cyanobacteria [61] and in the marine angiosperm *Zostera marina* L. [62], in which nitrate uptake is stimulated by salt stress. We speculated that specific *S. europaea* miRNAs, such as seu-miR24, are involved in regulation of nitrate transport and distribution under saline conditions, which is important for plant development under salt conditions.

### Potential application of *S. europaea* miRNAs to breed stress-tolerant plants and biofuel crops suitable for marginal lands

Biomass yield can be increased by manipulating miRNAs that target transcription factors and other regulators that control plant growth and development. For example, the overexpression of rice *miR156b* [63] and maize *miR156* (*Corngrass 1*) [64] in switchgrass can increase plant biomass and starch content and improve its biofuel conversion rate. In addition, the overexpression of *miR156* in rice, maize, and Brachypodium results in morphological alterations, including increased number of branches, thus providing a favorable phenotype for improved biomass production [65,66]. In the present study, we identified many *S. europaea* miRNAs targeting transcription factors (Additional file 12), which could regulate plant growth and development. We surmise that manipulating these miRNAs, similar to miR156 transgenics, can be used to promote biomass production.

Plant tolerance to abiotic and biotic stresses can be significantly enhanced by regulating the expression of specific miRNAs. For example, the constitutive expression of *miR319* leads to enhanced salt and drought tolerance in creeping bentgrass [67] and enhanced cold tolerance in rice [68]. miR319 is up-regulated in Arabidopsis by high salinity [37] but repressed in *P. tomentosa* [14] and rice inflorescence [19]. In *S. europaea*, miR319a-3p was suppressed in R-12 h and R-7 d (Figure 3B) and the expression of miR319b was up-regulated in the shoots and down-regulated in the roots (Figure 3E). The dissimilar expression trends observed in miR319 in different species indicate that their targets may play varied roles in different regulatory pathways in response to salt stress. Moreover, several highly abundant 22 nt miRNAs target genes encoding NBS-LRR plant immune receptors and trigger the production of trans-acting siRNAs in legumes and Solanaceae [69-71]; these findings provide novel evidence to improve plant immunity to diseases. The differentially expressed *S. europaea* miRNAs identified in the present study (Figure 3, Additional file 9) may be applied to improve plant tolerance to salt stress. Several *S. europaea* miRNAs targeted NBS-LRR resistance proteins (Additional file 12), particularly seu-miR1, which exhibits 22 nt length and is abundant in the six libraries. We speculate that these miRNAs may be applied to breed disease-resistant plants.

The production of ethanol from lignocellulose is usually hindered by lignin, a major component of plant secondary cell wall; lignin must be removed via energy consuming and environmentally unfriendly processes. Engineering plants with low lignin content or with lignin that can be easily broken down through transgenic strategies will solve this problem to some extent. On the other hand, lignin and cellulose deposition are also regulated in a compensatory mechanism. Reduced cellulose synthesis can activate lignin formation and defense responses [72], and a 45% reduction of lignin in transgenic *P. tremuloides* Michx. is compensated by 15% increase in cellulose [73]. In plants, miR397, miR408, miR857, and miR828 are implicated in regulating lignin content [74-77]. miR397 is induced by salt in Arabidopsis; this miRNA can directly cleave LAC (laccase-like proteins) and CKB3 (a regulatory subunit of casein kinase) transcripts [13]. In the present study, seu-miR397a was suppressed by salinity in *S. europaea* roots (Additional file 1) but was excluded in the differentially expressed miRNAs because of its low raw reads. Two laccase genes were the predicted targets of seu-miR397a, and one of these genes (unigene16755) was validated through our 5′-RACE experiment (Figure 6). Laccases (EC.1.10.3.2) are multi-copper oxidoreductases that catalyze the last step of lignin biosynthesis [78,79] to polymerize monolignols into a lignin polymer; this step is vital for the integrity of plant cell walls, strength of stems, and resistance against pests and pathogens. We previously found that salt can promote the development of *S. europaea* xylem and significantly affect genes involved in cell wall metabolism and lignin biosynthesis pathways [27]; hence, the promotion of lignin biosynthesis, xylem development, and cell wall structure modification is important for salt tolerance in *S. europaea*. In this study, the results further demonstrated that the repression of miR397-guided cleavage of laccase may be essential to adjust *S. europaea* lignin content under saline conditions. This mechanism may be one of the strategies used by *S. europaea* to survive under high-salinity habitats. In addition to the possible implication in cell wall modification, the overexpression of *miR397* in rice improves rice yield by increasing grain size and promoting panicle branching [80], thus improving the biomass. Seu-miR397 may be used to develop additional approaches for engineering biofuel crops with low lignin and high cellulose contents; these crops are important because they can be easily deconstructed and fermented into biofuels.

## Conclusions

In this study, we reported the systematic analysis of miRNAs in the euhalophyte *S. europaea*. The results revealed that specific miRNAs were strictly regulated in *S. europaea* shoots and roots under salt conditions, and thus, may play important roles in salt tolerance by regulating downstream targets. This study provided data to the database of novel miRNAs and elucidated the molecular mechanisms of salt tolerance in *S. europaea*. The findings of this study may be used for further analysis and applications in breeding practices. miRNAs, including salt-responsive miRNAs, in *S. europaea* and those that target transcription factors, NBS-LRRs, and enzymes involved in lignin biosynthesis, may be genetically engineered to generate plants with high biomass and improved stress tolerance that are suitable for marginal lands. These miRNAs can also be used to regulate lignin biosynthesis, thus producing energy crops with low lignin content and high cellulose that are suitable for bio-ethanol production.

## Methods

### Plant material

*S. europaea* was grown under conditions according to our previous studies [27]. Thirty days later, the plants were salinized with 200 mM NaCl for 0 h, 12 h and 7 d. To make sure all materials collected having the same biological age and growth rhythm, they were treated separately with NaCl at different time points while harvested at the same time.

### Small RNA isolation and Illumina sequencing

Total RNAs of the six libraries were isolated from whole shoot and root tissues of *S. europaea* as described previously [81], except that in the final step, one volume of isopropyl alcohol was used to precipitate the RNA, instead of LiCl. After treated with RNase-free DNase I (Takara, Japan), the quality of total RNA was measured by agarose electrophoresis and the Agilent 2100 Bioanalyzer. All RNA samples were submitted to BGI (Shenzhen, China) for high-throughput sequencing using Illumina high-throughput sequencing platform.

### sRNA sequence processing

The raw data were processed with BGI sRNA analysis pipeline to filter out artifact sequences. Non-redundant sRNAs ranging from 18 to 30 nt were collected and stored in the Clean file, which were mapped to *S. europaea* mRNA transcriptome database using SOAP, according to its default settings (http://soap.genomics.org.cn). rRNA, tRNA, snoRNA and snRNA sequences were downloaded from NCBI and Rfam 9.0 (http://rfam.sanger.ac.uk/), while coordinates of genomic repeats were obtained from RepeatMasker (http://www.repeatmasker.org/PreMaskedGenomes.html). The perfectly aligned sRNA was annotated as rRNA/tRNA/snRNA/snoRNA, miRNA, repeat element, exon-sense, exon-antisense, intron-sense or intron-antisense and stored in separate files, based on the annotation of the sequence which it overlapped in *S. europaea* transcriptome. Matching sRNAs without annotation were stored in the un-annotated file.

### Identification of conserved and novel miRNAs

To identify conserved miRNAs, unique sRNAs from sRNA library and contigs from *S. europaea* mRNA transcriptome database were utilized in local BLASTn analysis (E value was set to 0.01 and mismatches were set to less than 3) against the mature and precursor sequences of miRNAs in miRBase version 20.0 (http://www.mirbase.org/) [30]. The unique sRNAs were aligned to *S. europaea* mRNA transcriptome database using MIREAP with default parameters (http://sourceforge.net/projects/mireap/). BLASTn searches against all nucleotide sequences in NCBI databases were performed to investigate whether these potential miRNA precursors were conserved in other plant species. Putative precursors homologous to known plant rRNAs, tRNAs or mRNAs were excluded. MFOLD was used to predict the secondary structures of the candidate miRNA precursor sequences, utilizing default parameters (http://mfold.rna.albany.edu/) [82]. Only the perfectly matched sRNA sequences and homologous sequences with precursor sequences were considered to be conserved miRNAs. Sequence with proper secondary hairpin structures and no homologous sequence in public databases was considered as putative novel miRNA precursor sequence. The miRNA precursor sequences should meet the following criteria: (1) forming an appropriate stem-loop structure, with mature miRNAs sitting in one arm; (2) mature miRNAs had less than 6 mismatches with the opposite miRNA sequences in the other arm; (3) the minimal folding free energy of the hairpin structure was less than or equal to −15 kcal/mol; (4) the MFEI values were more than 0.40 [36].

### Expression analysis of miRNAs under salt treatment

All miRNAs were normalized to transcript expression levels per million reads (RPM). If the raw read of one miRNA in a library was zero, the normalized RPM was adjusted to 0.01. Normalized miRNA reads with values less than 1 were excluded from the differential analysis. The remaining normalized reads were used to calculate the change in miRNA expression and *P*-value. Fold change = Log_2_ (salt treatment/control) or Log_2_ (root/shoot). A 2 × 2 contingency table was used to perform Pearson’s chi-squared test for significance of miRNA expression from two samples. miRNAs with *P*-value less than 0.05 and fold change greater than 1 or less than −1 were considered to be significantly altered [10].

### Cloning and sequencing of pre-miRNA sequences

Total RNA was isolated from 2-month-old *S. europaea* seedlings as described above. cDNAs were synthesized from 2 μg of purified total RNA in 25-μL reactions, containing 200 U M-MLV reverse transcriptase (TransGen, China) and 1 μg Oligo d(T), according to the manufacturer’s protocol. Thirty-five pairs of primers for *S. europaea* precursor sequences were designed (Additional file 13). PCR amplifications were carried out, using the following thermal cycling conditions: 94°C for 5 min, 35 cycles at 94°C for 30 s, 55°C or 60°C for 15 s and 72°C for 50 s. Amplification products were separated on a 2% agarose gel with ethidium bromide (EtBr) staining. Gel-purified PCR fragments were subcloned into T1-simple Vector (TransGen) and sequenced.

### Real-time quantitative RT-PCR

Total RNAs were isolated from the shoot and root tissue of *S. europaea* treated with 200 mM NaCl for 0 h, 12 h and 7 d as described above. The RT reaction mixture contained a 1 μg aliquot of total RNA and a mixture of 0.2 μL of each RT primers (10 μM) for all of the mature miRNAs and U6 snRNA, which was chosen as a reference controle (Additional file 13). The mixture was incubated at 80°C for 5 min, 60°C for 5 min and then frozen on ice for at least 5 min. The remaining reagents (2 × reaction mix, RT enzyme mix) were then added (TransGen). The reaction was continued at 16°C for 30 min, followd by 60 cycles of 30°C for 30 s, 42°C for 30 s, and 50°C for 1 s, ending with 85°C for 5 min. SYBR® Green Realtime PCR Master Mix (Toyobo, Japan) was used to detect miRNA expression by a Stratagene Mx3000p Detection System (La Jolla, CA, USA). Briefly, cDNAs were diluted 50 times and 1 μL diluted sample was used as template in a 10 μL PCR reaction, which contained 5 μL 2 × SYBR Green Realtime PCR Master Mix and 0.25 μM of a miRNA-specific forward primer and universal reverse primer. The quantitative PCR was conducted in triplicate for 90 s at 95°C, then 40 cycles of 15 s at 95°C and 10 s at 60°C [34]. For each PCR, dissociation curve analysis was carried out to discriminate specific products from primer dimers. The fold changes of miRNA in different samples were calculated byΔCt method as described.

### Target genes prediction

Web-based psRNATarget program was used to identify putative targets for conserved and novel miRNAs (http://plantgrn.noble.org/psRNATarget/?function=3). The custom plant transcript databases include 57151 unigene sequences from *S. europaea* mRNA transcriptome database [27]. Sequences with a penalizing score ≤3 were chosen as putative targets [31].

### 5′-RACE of miRNA cleavage

Total RNA (1 μg) from equally mixed 6 RNA poles was used to synthesize 5′- RACE-ready cDNAs with N-15 random primer mix and BD Smart RACE cDNA Amplification Kit (Clontech, CA, USA) according to the manufacturer’s instruction. The first round of PCR involved 10×UPM, outer gene-specific primers and PCR Polymerase (TransGen). The product was diluted 50 times and then used as template for the second round of PCR, which involved NUP and outer/inner gene-specific primers. Amplicons were separated from the gel, then cloned into T1-simple vector (TransGen) and sequenced. The outer and inner gene-specific primers were listed in Additional file 13.

### Availability of supporting data

The data sets supporting the results of this article are available in the Gene Expression Omnibus repository under accession no GSE62521 (http://www.ncbi.nlm.nih.gov/geo/query/acc.cgi?acc=GSE62521) [83].

## Additional files

Additional file: 1. The conserved miRNAs identified in *S. europaea*. Additional file: 2. The length distribution of *S. europaea* conserved miRNAs. Additional file: 3. The precursor and primary sequences of *S. europaea* conserved miRNAs. Additional file: 4. The hairpin structures of *S. europaea* conserved miRNAs predicted by MFOLD. The mature miRNAs were highlighted in yellow. Additional file: 5. The novel miRNAs identified in *S. europaea*. Additional file: 6. The precursor and primary sequences of *S. europaea* novel miRNAs. Additional file: 7. The hairpin structures of *S. europaea* novel miRNAs predicted by MFOLD. The mature miRNAs were highlighted in yellow while miRNA star sequences were highlighted in green. Additional file: 8. The length distribution of *S. europaea* novel miRNAs. Additional file: 9. Differentially expressed miRNAs in *S. europaea*. Sheet 1: Differentially expressed miRNAs in *S. europaea* after salt treatment. Sheet 2: Differentially expressed miRNAs in *S. europaea* between shoots and roots. Additional file:10. The targets of *S. europaea* miRNAs. Sheet 1: The targets of *S. europaea* conserved miRNAs. Sheet 2: The targets of *S. europaea* novel miRNAs. Additional file: 11. The summary of salt-regulated miRNAs in *S. europaea*. Additional file: 12. The *S. europaea* miRNAs that have potential for improving plant stress tolerance and engineering bioenergy plants with improved properties as biofuel feedstock. Additional file: 13. The primers used in this study.

## Abbreviations

AGO: Argonaute
DCL: Dicer-like
MFEI: Minimal folding free energy index
miRNA: microRNA
NBS-LRR: Nucleotide binding site-leucine-rich repeat
pre-miRA: Precursor miRNA
pri-RNA: Primary miRNA
RACE: Rapid amplification of cDNA ends
RISC: RNA-induced silencing complex
RPM: Reads per million
sRNA: Small RNA
siRNA: Small interfering RNA
snRNA: Small nuclear RNA
snoRNA: Small nucleolar RNA
