# Supplementary figures and images for: High-throughput deep sequencing reveals that microRNAs play important roles in salt tolerance of euhalophyte Salicornia europaea
Source: BMC Plant Biol. 2015 Feb 26;15:63. doi: 10.1186/s12870-015-0451-3 (PMC4349674; doi:10.1186/s12870-015-0451-3)

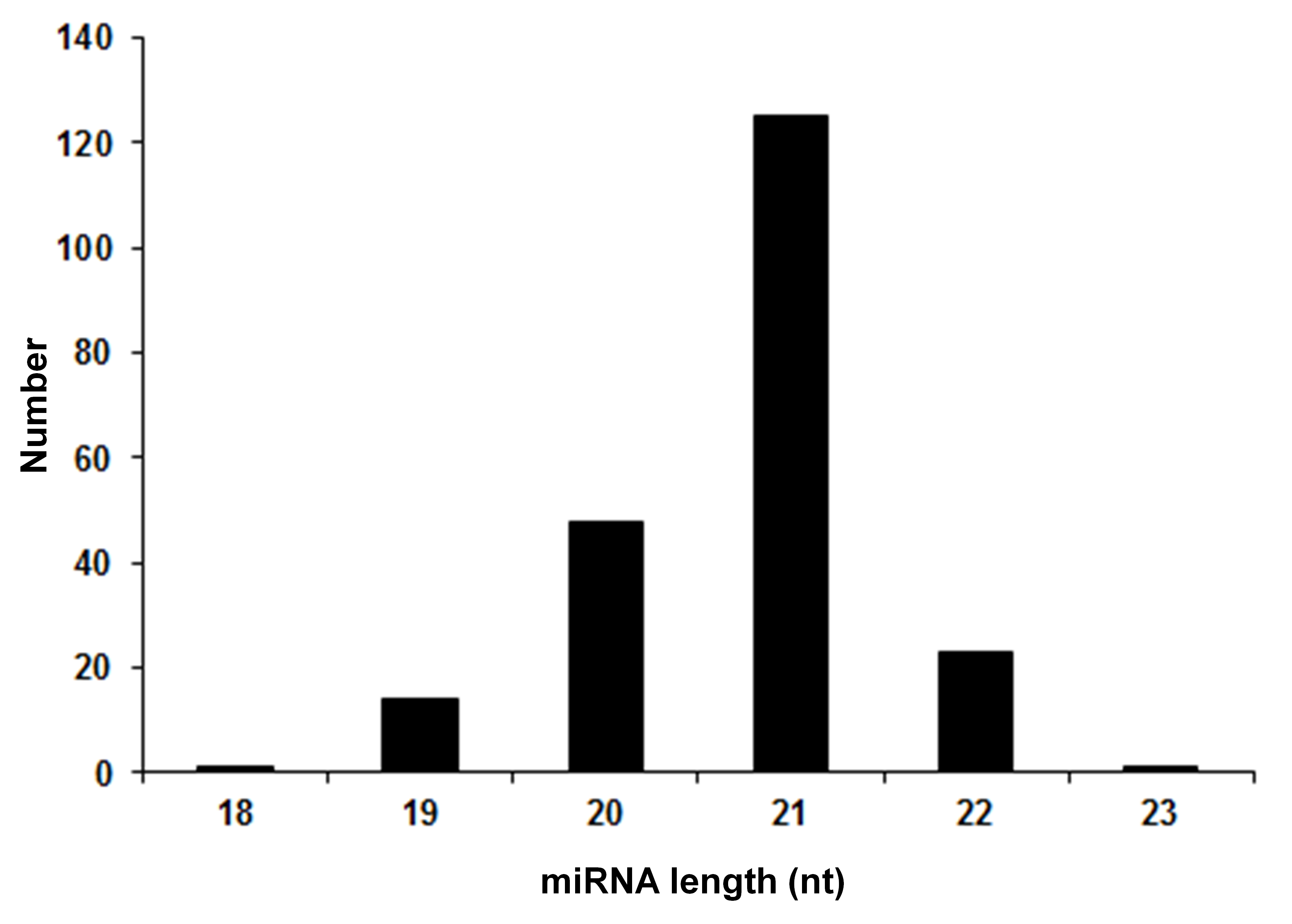

Supplement: Additional file: 2. — The length distribution of S. europaea conserved miRNAs. [file 12870_2015_451_MOESM2_ESM.tiff]

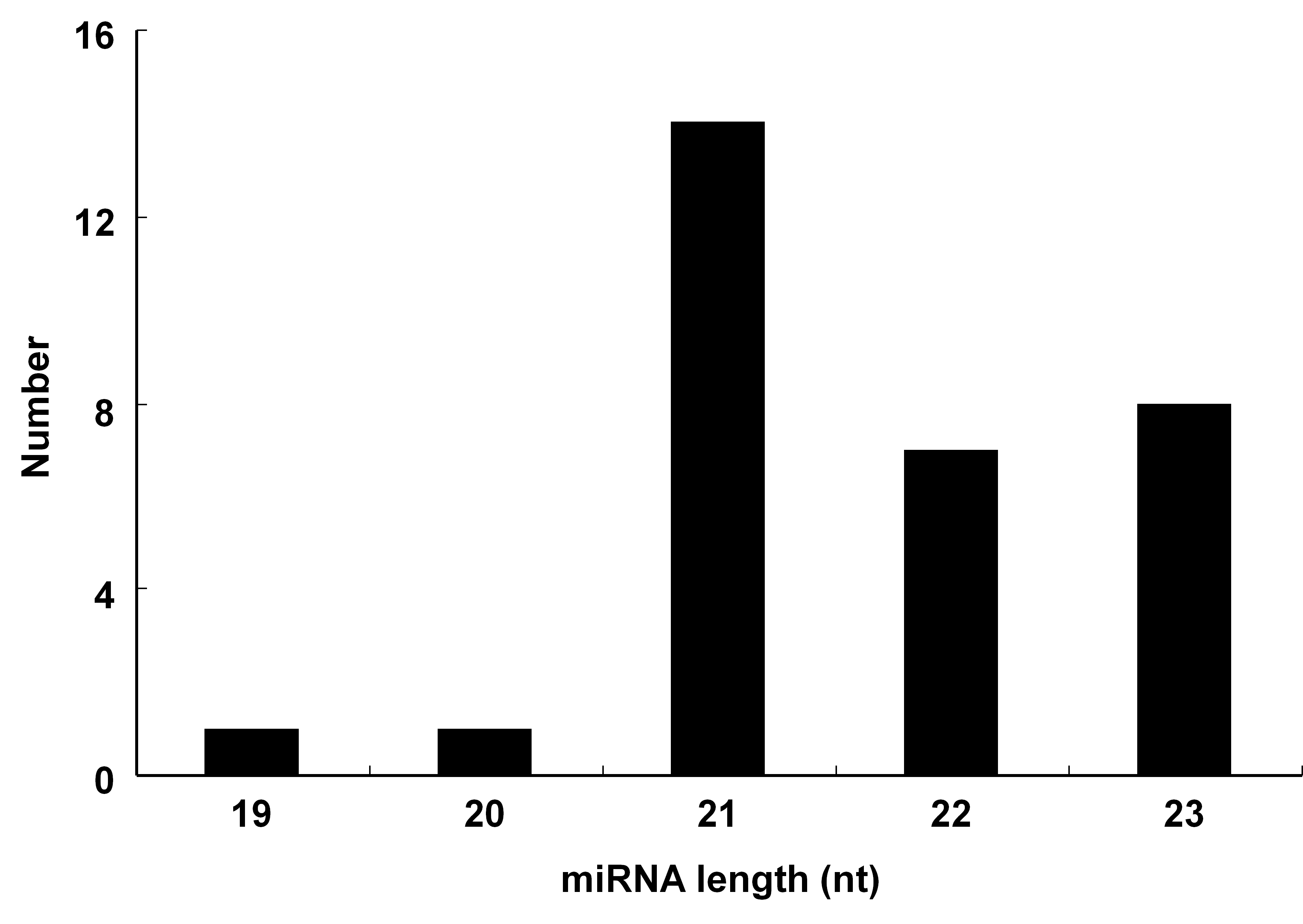

Supplement: Additional file: 8. — The length distribution of S. europaea novel miRNAs. [file 12870_2015_451_MOESM8_ESM.tiff]
