# Supplementary material for: High-throughput deep sequencing reveals that microRNAs play important roles in salt tolerance of euhalophyte Salicornia europaea
Source: BMC Plant Biol. 2015 Feb 26;15:63. doi: 10.1186/s12870-015-0451-3 (PMC4349674; doi:10.1186/s12870-015-0451-3)
Supplement: Additional file: 3. — The precursor and primary sequences of S. europaea conserved miRNAs. [file 12870_2015_451_MOESM3_ESM.doc]

**Additional file 3. The precursor and primary sequences of *S. europaea* conserved miRNAs.**

| **miRNA name** | **Sequence (5'-3')** | **Length (nt)** |
| --- | --- | --- |
| pre-miR156a | GAAGGGGCGGUGACAGAAGAGAGUGAGCACACAUGGCAAUUGUAUCGCACUAUGCUGUUCCUUUUUUGUGCUCACUGCUCUUUCUGUCAGCUUCCCUUAAU | 101 |
| pri-miR156a | UUUCUUCCCAGAAUGAAACAUAAUAAUUAUAAUCAGAACAAACAAAAAAAGGUAAGAAGGGGCGGUGACAGAAGAGAGUGAGCACACAUGGCAAUUGUAUCGCACUAUGCUGUUCCUUUUUUGUGCUCACUGCUCUUUCUGUCAGCUUCCCUUAAUCUUCAAUCCAACCUCCAUCAUCGACUACUUCCUAAUUUCUUGGGAUAAAAUAGUAAUUCCACUACUACUUAUUAGUUAUUACUUCUCUAUUUAAUUGUUUUUUUUAAUUUUUUCUUUCUUCCAAAUUCCAAUAAUUAAAAUUGAUUGCGCGACUUUUGAGGGAAC | 321 |
| pre-miR164a | GGUUAAGGUGUUGAGCUAGAUGGAGAAGCAGGGCACGUGCAUUACUAACCCGUGCAAUGUACAAAUAUCGACGCAUAUGCUUGCAAGCAUAUACGUCGGCUGAUAUAAAUAUGGAUACAAAUAUAGAUACCGAUACAGAUUUUGGUGUCCGAAUUCAGUCUUCUACCUAUCUUUUUAUAUCAAAUAAAUUGUUGCAAUGAGUUAGUGCUUUCAUGUGCCCAUCUUCCCCAUCAUGCCCACUUGACCUAGUUU | 252 |
| pri-miR164a | CUUUUAAUUUCUCUUGCUUUCUCCUUUAUCUUCCACUAUAAAAUUCAACUUCUCUCUAGCUAGCUAGUUUUUUUUUGUUUUUUUUUUUUUAAAUUAGUGAUAAAGGCUUGCCUAUGAUAUAAUGGUGAGAAGCAAACAACCAAUAAGGUUAAGGUGUUGAGCUAGAUGGAGAAGCAGGGCACGUGCAUUACUAACCCGUGCAAUGUACAAAUAUCGACGCAUAUGCUUGCAAGCAUAUACGUCGGCUGAUAUAAAUAUGGAUACAAAUAUAGAUACCGAUACAGAUUUUGGUGUCCGAAUUCAGUCUUCUACCUAUCUUUUUAUAUCAAAUAAAUUGUUGCAAUGAGUUAGUGCUUUCAUGUGCCCAUCUUCCCCAUCAUGCCCACUUGACCUAGUUUACAAUUCAAGCUAGCUUCAAUUCAUUAGCUCCUCAAUUGAUAGAUAUGGUUUUUUCCUUUUCAUUCAAAUCAUAUUGAUGUCUUCAAGCUUCAUUGAUUCUUAUUGGUUUCAGAUAAAGGAAGGGGUUGCAAGUAAUUAAUAAGGUUUGAACUCCGUAUCACAAGAGUAAAGGAUAGCUCAGCAGGUGGUUA | 590 |
| pre-miR166a | AGGAAGCAUAUGAGUUGAGGGGAAUGUUGUCUGGCUCGAGGACUAGAUUGUUUGGCAGUAGAUCUAUUUCCACCCCUUUUCCCGUCGUCGGACCAGGCUUCAUUCCCCCCAGUUUUUCCUGCUUCCCA | 128 |
| pri-miR166a | UCCCUACUCUUUUCUCUGUCCUUGUCUUUCCCCCACUCUCCCCCUCUUCUCCUUACUCUCUCUCUCCUCCCUCCUUGAGAUUAGCCGAUUUUCAUGGGGGUAUCCCUCUACAAAUAAAUAAAUAAAUAAAAAAAAAAAAAGAAAAAAAAAAACGCAGAAGAAAGGAAGCAUAUGAGUUGAGGGGAAUGUUGUCUGGCUCGAGGACUAGAUUGUUUGGCAGUAGAUCUAUUUCCACCCCUUUUCCCGUCGUCGGACCAGGCUUCAUUCCCCCCAGUUUUUCCUGCUUCCCAAACACAACCAAUUCAAGUUGAUAUGUGAUGAGAAAACUCAGUUACUGAAGAAGACCAGAGAAAACCAAAAAGGUCACAGUUGAUUUGAAAAGAUAAGACUUUACUACCUAGC | 402 |
| pre-miR168a | CUGAUUCGCUUGGUGCAGGUCGGGAACUGGUCUGUCUGAUUGAUUAAAUCUUCCUCCUUCGCCGCUGAAAUCGCUGUGUAUGGCGGCGAAGAUUGGUUUUUAAUUUUUCGCCGGUUCAUCAACCGUUGCAAAUGGCGGUGUAGCUGAUUUAUCUUUAAUUUAUUCGGUUGGUGGAUUUCCAUUUCCCG | 188 |
| pre-miR172b | UCAACAGCCGUUGUUUGCCGAUGCAGCACCAUCAAGAUUCACAUACCCUCCUAAAAAAAAAAAAAUUGUUGAAGGAGUUCAAGGAUUAGCAUAGGAUGUGAGAAUCUUGAUGAUGCUGCAGGGGCAAUAAAUGGCUAAU | 139 |
| pre-miR319a | AGAGAGCUUCCUUCAGCCCACUCAUGGAUGGAAUUAAGGGGGUUUGAAUUAUCUGCCGACUCAUUCAUUCAAACACUCAGUAGAACGAAACUUUUACAACGAGGUGCUACUGUGAUUGCGUGAAUGAUGCGGGAGAUAGUUUCAUCCUUCUCCUUCUGUGCUUGGACUGAAGGGAGCUCCCUUUCUCCAUCU | 192 |
| pri-miR319a | AGAGAGCUUCCUUCAGCCCACUCAUGGAUGGAAUUAAGGGGGUUUGAAUUAUCUGCCGACUCAUUCAUUCAAACACUCAGUAGAACGAAACUUUUACAACGAGGUGCUACUGUGAUUGCGUGAAUGAUGCGGGAGAUAGUUUCAUCCUUCUCCUUCUGUGCUUGGACUGAAGGGAGCUCCCUUUCUCCAUCUAUUUCCUUCAUUCUUGCGCACUUAAUAUGUCAAAUCGUUGCAGUUAAUAUGU | 244 |
| pre-miR393a | GUAGGAGGAAUCCAAAGGGAUCGCAUUGAUCCCAACUGACUAUCUAUCUAAGGCCUUGUUUGGAUCAUGCAAUCCCUUUGGAUUUAUCCUUC | 92 |
| pri-miR393a | AGCCAAACAAGAGAGAGAAACAAAGAGUGUUUAUUUUUAUUUUUUUUGUGGGGGGUUUGUUUAAUUUUACAGAAGCGCAGCAACUGUAGGAGGAAUCCAAAGGGAUCGCAUUGAUCCCAACUGACUAUCUAUCUAAGGCCUUGUUUGGAUCAUGCAAUCCCUUUGGAUUUAUCCUUCCGUAGCUUUAAUUUAUGGCUGUAUUAUUCCCAACUUGAGAAAACAGGGCUGCAACCAGGUCCAACCCAGUCACGGCAGCCUUGCCACCC | 266 |
| pre-miR398a | CGAAAUCCGACAGGAGCGACCUGAGACCACAUGGAUGAGGCAUCUGGAUUUUGGGAAGAAUGAUUGCUAUCCUGUGCUGUUCAUGUGUUCUCAGGUCGCCCCUGCUGGGCUUCC | 114 |
| pri-miR398a | UCACAUAUUCAAUCAUUUAAAAAAAAAAAAUUAAAUGAGCGAGUUAUUUUAGGUGACGAAAUCCGACAGGAGCGACCUGAGACCACAUGGAUGAGGCAUCUGGAUUUUGGGAAGAAUGAUUGCUAUCCUGUGCUGUUCAUGUGUUCUCAGGUCGCCCCUGCUGGGCUUCCUCCACCAUAUAAUUGAUAGGUCGAUUUGAAAUUAAGAUCCCAUCUCCAGAAAUCAGCCCAGAUAAACUGGUUCAGAUACUGCAAGGUAAAAAAUUCUUCAGAAGUAAAAGUAAGUAGCAUAAAUUUGACAAGACUAAUUAAUUAAUUAAUUUAAAACCAUAAACACCAACUUUCUGAAAGUAAUAAAAAUCAUCUACAUUUCAUAAGACUAAACAAAAUUGACUAAUACAAAAGCAUACUAGCAAAACUUUUCUUCUAGAAAAUGACACGAUAAUAUCGAAGCUAAACUAAGAUCUAACAGAUGAUAGCUUUUAUAGCAAAGGUUAGAAAGGAGGCAAGUUAAGUUCAAACCUCAAUCUUUGUUUUGGUCACUAAAAUGCUAAUGCGGGAUUUGAUAUCUUGUAGUUCUUGAUUUCCCACUUUUUGCCAAACCCCACCAAUUAAAAGUGAAGCUUUUAACCUGUCAGGUGUUUCAACAGAAUUUGAAUUCUCUGAAUCUUGUAUCUCUCUCAUUCUCUUAAUACCC | 696 |
| pre-miR399d | UAGACAUGAACCAAAAGGAGUCACUCAACUGAAACUUGAAAUACUAUUUUGCCAAAGGAGAUUUGCCCUAAAGAGCAAAAGUUUGAUUUGUAUAAUUAUUUUUUUUUGUAUAAAGUUAUGCUGAAAGAAUAGAGCUUCUUUAAGAGCACAAUUUAUAUUGCCAAAGGAGAU | 171 |
| pre-miR2911a | UCCCAGUCCCGAACCCGGCGGCUGUCGGUGGACUGCUCGAGCUGCUCCCGCGGCGAGAGCGGGGCGCCGCGUGCCGGCCGGGGGGCGGACUGGGA | 95 |
