# Supplementary material for: High-throughput deep sequencing reveals that microRNAs play important roles in salt tolerance of euhalophyte Salicornia europaea
Source: BMC Plant Biol. 2015 Feb 26;15:63. doi: 10.1186/s12870-015-0451-3 (PMC4349674; doi:10.1186/s12870-015-0451-3)
Supplement: Additional file: 4. — The hairpin structures of S. europaea conserved miRNAs predicted by MFOLD. The mature miRNAs were highlighted in yellow. [file 12870_2015_451_MOESM4_ESM.pdf]

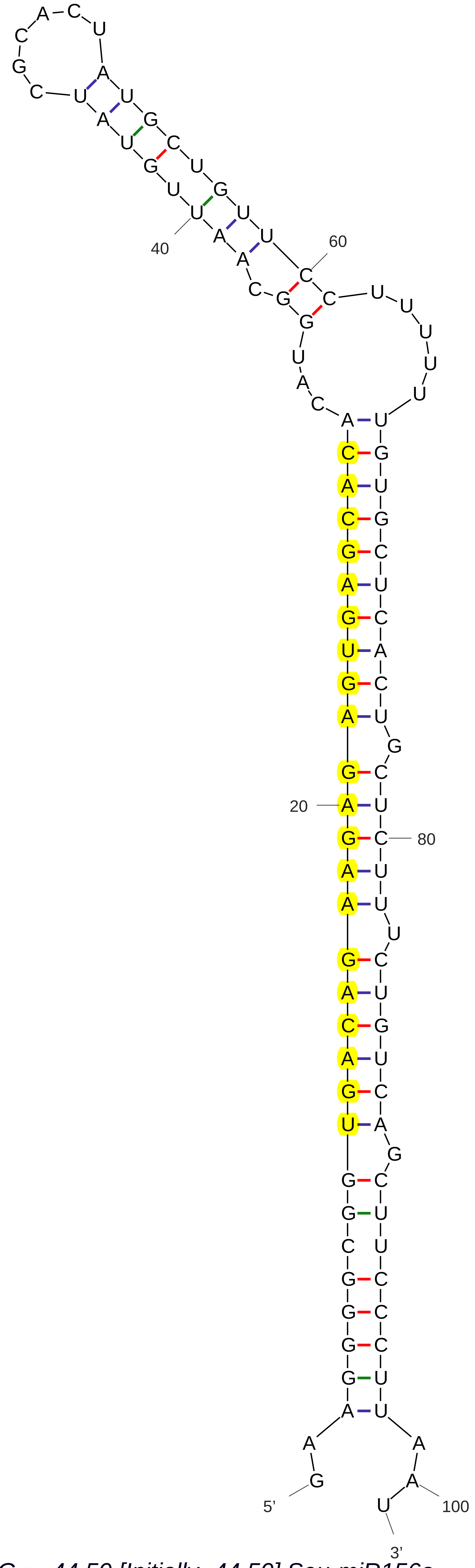

dG = -44.50 [Initially -44.50] Seu-miR156a

Output of sir_graph (©)
mfold_util 4.6

Created Thu Dec 27 21:33:27 2012

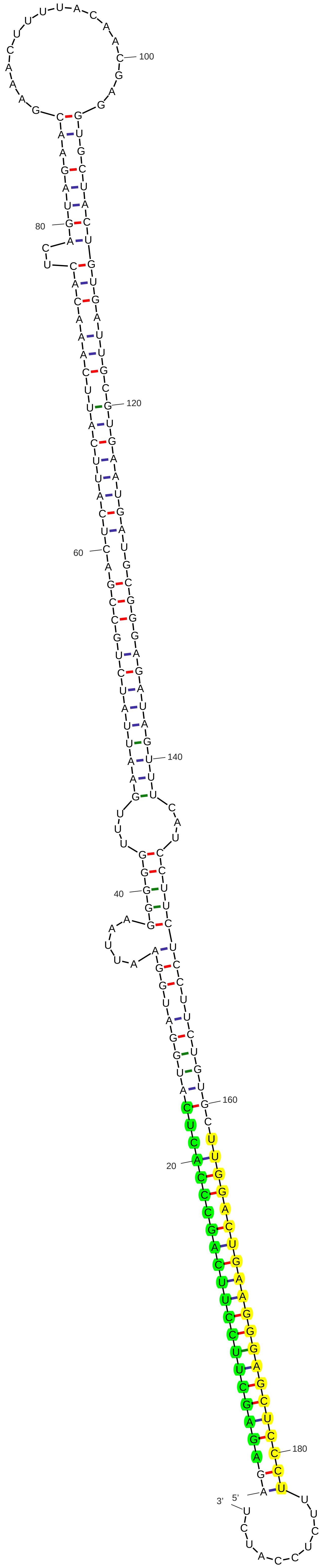

dG = -77.60 [Initially -77.60] Seu-miR319a

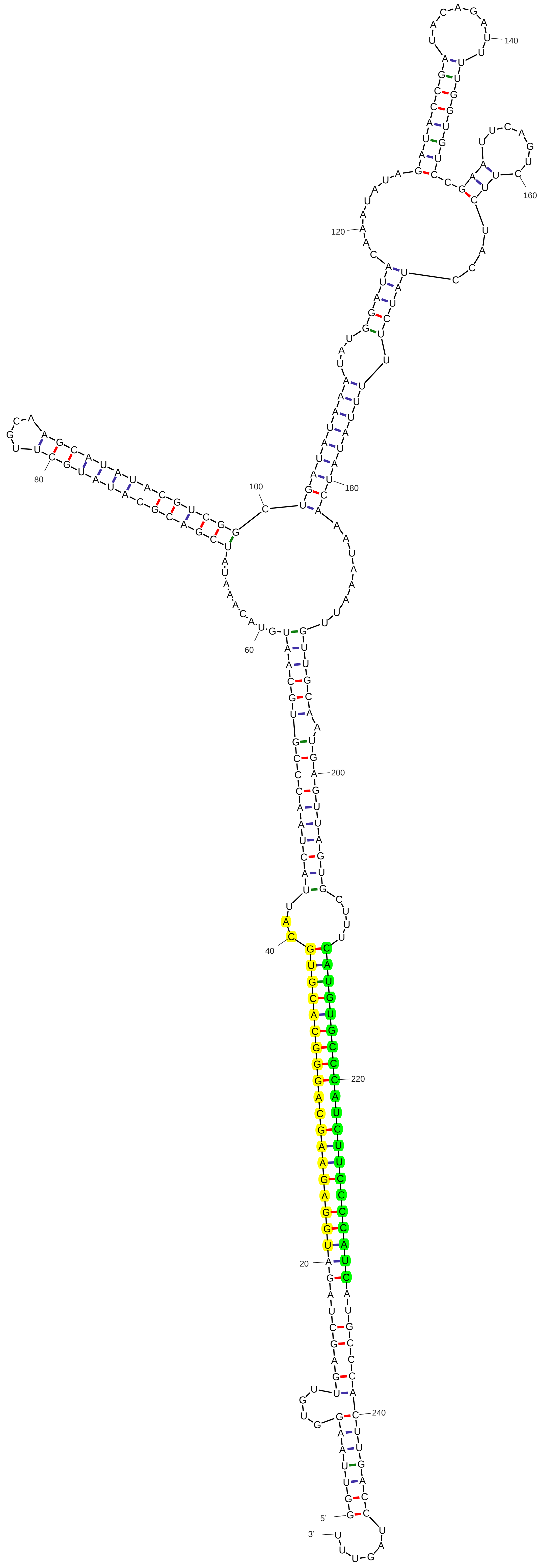

dG = -81.74 [Initially -87.80] Seu-miR164a

Output of sir_graph (©)
mfold_util 4.6

Created Thu Dec 27 22:24:37 2012

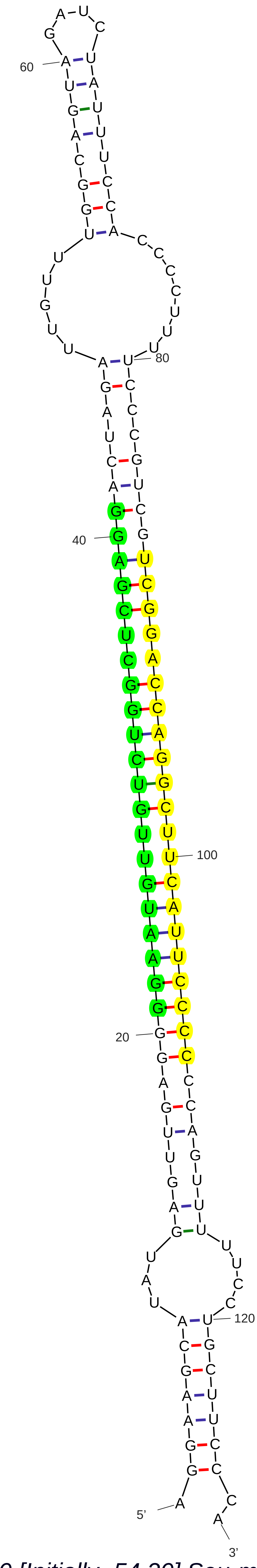

dG = -54.20 [Initially -54.20] Seu-miR166a

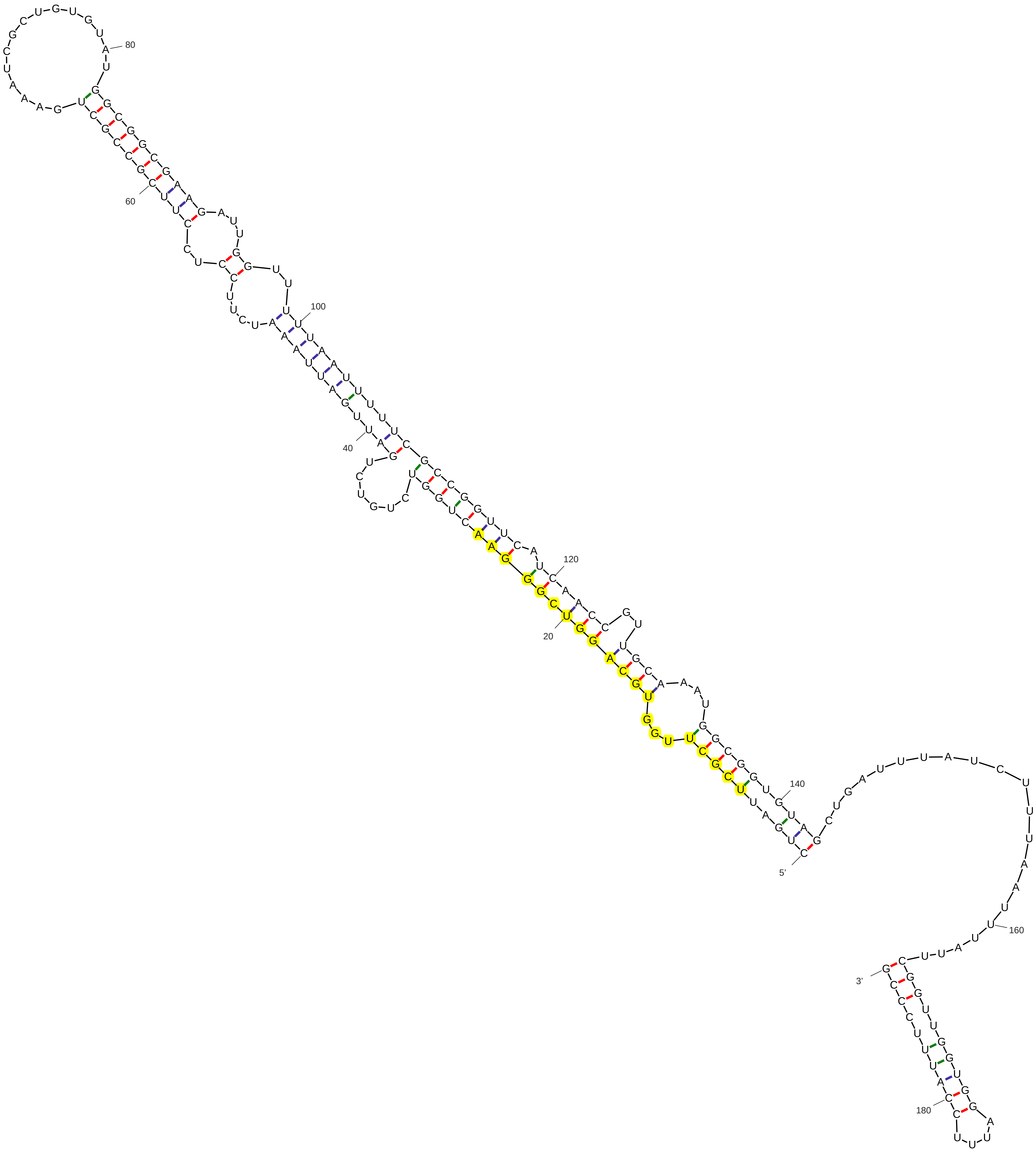

dG = -60.70 [Initially -60.70] Seu-miR168a

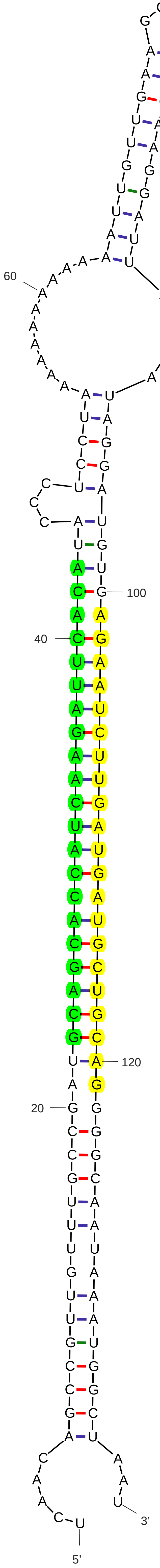

dG = -57.40 [Initially -57.70] Seu-miR172b

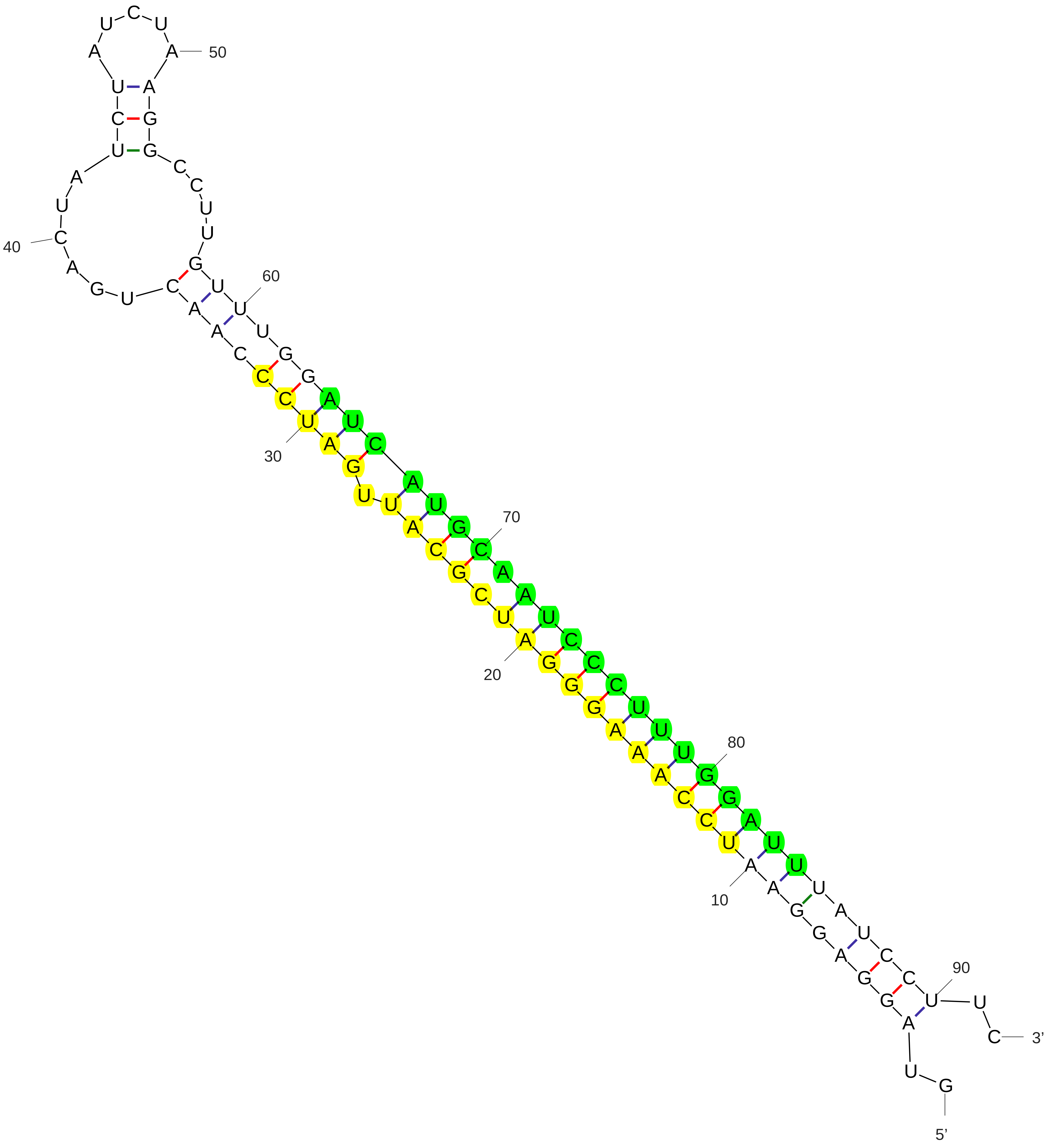

dG = -41.00 [Initially -41.00] Seu-miR393a

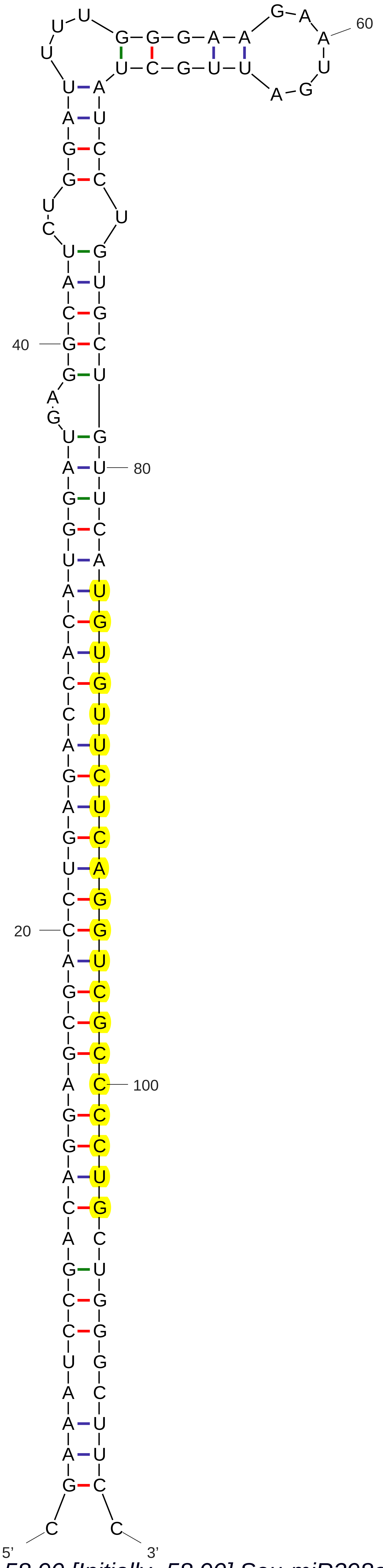

dG = -58.90 [Initially -58.90] Seu-miR398a

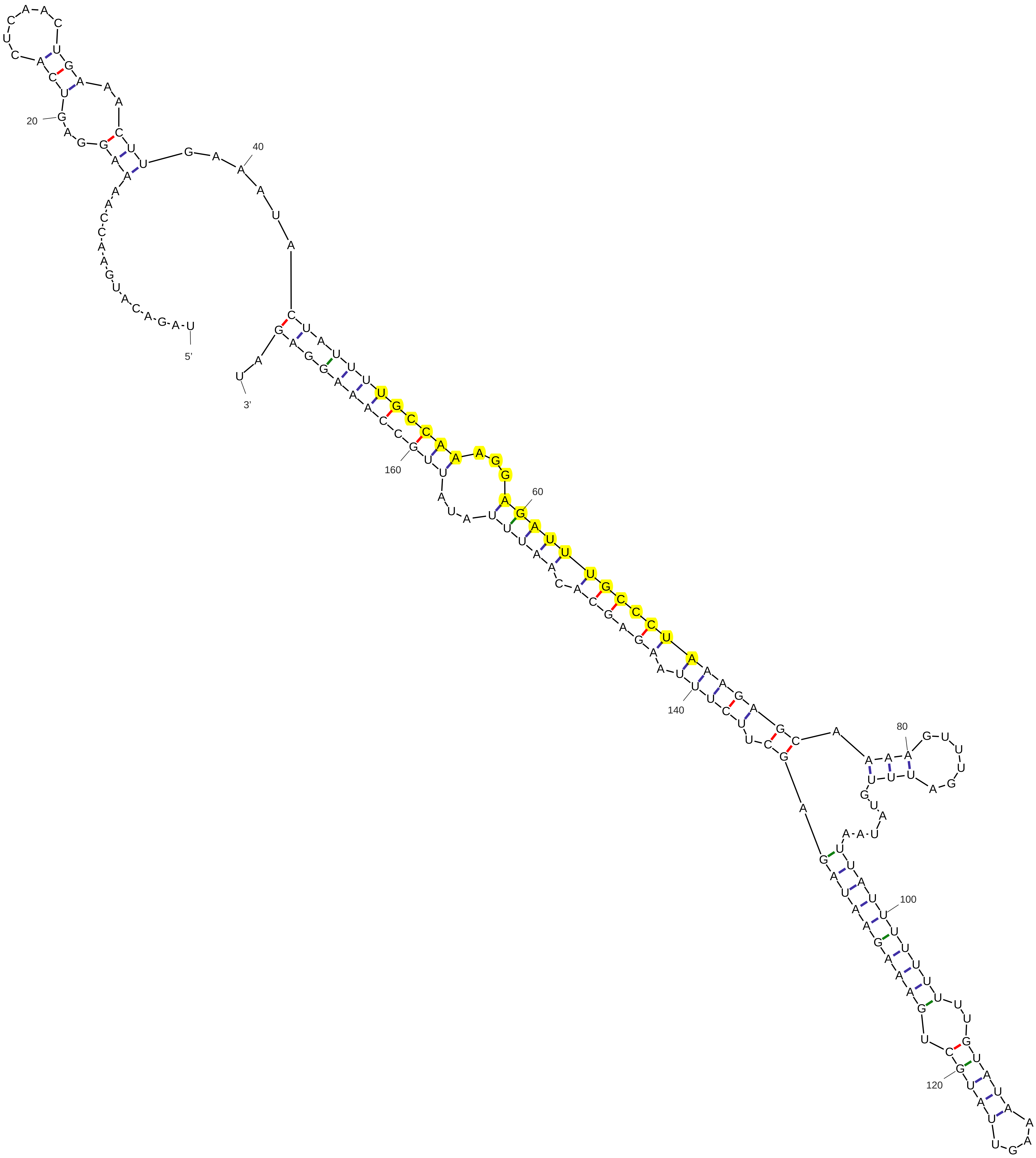

dG = -23.18 [Initially -25.50] Seu-miR399d

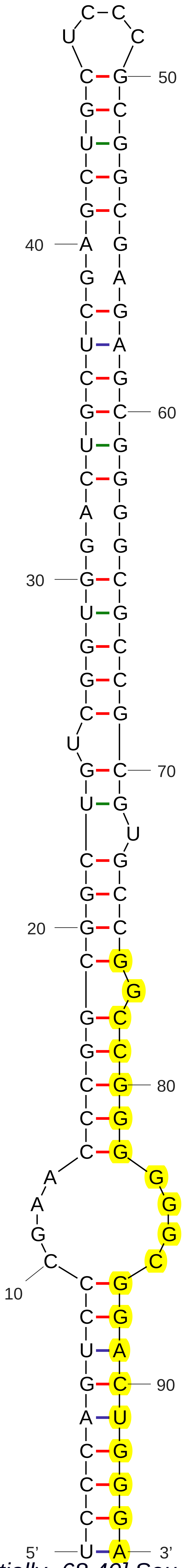

dG = -68.90 [Initially -68.40] Seu-miR2911a
