# Supplementary material for: High-throughput deep sequencing reveals that microRNAs play important roles in salt tolerance of euhalophyte Salicornia europaea
Source: BMC Plant Biol. 2015 Feb 26;15:63. doi: 10.1186/s12870-015-0451-3 (PMC4349674; doi:10.1186/s12870-015-0451-3)
Supplement: Additional file: 6. — The precursor and primary sequences of S. europaea novel miRNAs. [file 12870_2015_451_MOESM6_ESM.doc]

**Additional file 6. The precursor and primary sequences of *S. europaea* novel miRNAs.**

| **miRNA name** | **Sequence (5'-3')** | **Length (nt)** |
| --- | --- | --- |
| pre-miR1 | UUGUUGAAAAUCUCUUCAAAUGUUCUCGCUGGCUAUAGCUAGAUAUAGCAAGCGGAACAUUUGCACAAUUUUUAACA | 77 |
| pri-miR1  (unigene15614) | GAGAAGAAAACCCUAACUAUGUCAAGAGCGGGUAGAGAUUGUUGAAAAUCUCUUCAAAUGUUCUCGCUGGCUAUAGCUAGAUAUAGCAAGCGGAACAUUUGCACAAUUUUUAACAAUCUCUUUCCCGAUCUUGACUUCCGUUAGACGCUUUGUUGUUUGUAUGUUGUUUGAUUCACUUACUGCAGAUCUAAUAGGUAUCCUUUUUGCGUUUCUUUUUCAUAAUAUAAAAGUUUAGAUUCGAUCUCAAUUUUGCGGUGCUGGAUUUUAGAUUUGUAGUAGUUGGAGAAGGGAAGAGAACGAUAGUUGUGGCUUUCGUGUUUUUAAUUUUUUUUCCACUAAAGCUUAACGAUCUAAUCUUUUAUUGUGAUUUUGAUCUGAAAAAUUACUUCGAUUUUGAAGCUAUGCAGUAUGAAUACUACAGAUAAUGUUAUCAACUUGUAAAAAU | 445 |
| pre-miR2 | UCGAGGCUCUAGUGCUUCACCCUCACUAGCUACGGCCUACGCGUCCUACUGAACAUAACAAAGUAACCAGACCUAAAGGGUCUAAACCCCCCCAAGCUAGGAAAAAAGCAUCGUCACGAGGCUGUGGGUGGAGAGGUCUAGAUCUCG | 147 |
| pri-miR2  (unigene43588) | UUCACUUAAAUACAAGUGAGAACUUACCUGUAGAGGACAUGAAUUGGUUUGUCGGGAAACCUGUAGUCCAGAUGCACCAUUCACACUUUUAAAGCGCCUUUAACGCUACCGAAAUCAUUCCUCGAUCGAGGCUCUAGUGCUUCACCCUCACUAGCUACGGCCUACGCGUCCUACUGAACAUAACAAAGUAACCAGACCUAAAGGGUCUAAACCCCCCCAAGCUAGGAAAAAAGCAUCGUCACGAGGCUGUGGGUGGAGAGGUCUAGAUCUCGGGGUCAUCCUGGGGGGAAGGGUGUUGGAAGAAGUGACCUUCUGACACGUGGUAGUCAUACAUCUGCUCAGACUGAAUACGGAGACGGCUCAAGUCAGAACGCAUCUCCAUAAUGCUAGCGUUAAUGGACUGGAAAGCAGCAUUUUGCUGCUGGGAUAGGGCCUC | 436 |
| pre-miR3 | GUUUUCCCUAUUUUACAUCGUUUACUUUGGGUAAAAAAAACAAAAUGUAAAAAUUGGGUAAAACCUCAUUUUACACCCUUUUGAAAACAUUUUUUCCCCAAAUUUGGGAGUCAAAAGUAUUUUCCGUGCAAGUAACCAUUGGUAAAUUUCUAUGAAAAUGAGUUUUCCAUGGUAAAAUGUUUUUCGUGGAAAACC | 195 |
| pri-miR3  (unigene15970) | GCGUGGAAGAGAUAACUUGAUUCGAAACUGCUUGACAUGCACAAUGAAAGCCAGAAAAGGAAGUUAAUUAUCAAGCAUGAAUUGAGGCUCCGUACACUAGAGCGGAAAAUAUUUUCCUUAAAAACAGUUUUCCCUAUUUUACAUCGUUUACUUUGGGUAAAAAAAACAAAAUGUAAAAAUUGGGUAAAACCUCAUUUUACACCCUUUUGAAAACAUUUUUUCCCCAAAUUUGGGAGUCAAAAGUAUUUUCCGUGCAAGUAACCAUUGGUAAAUUUCUAUGAAAAUGAGUUUUCCAUGGUAAAAUGUUUUUCGUGGAAAACCAUUUUAUACCAAAAACGUUUUACACCCAAGUAUACGGAGCCUGAAUUCUAAAUAACUUAUUACUCUUAGCAUUUUAUAUAAAUAUGACAUAACAGCCAUUAUCCUUCAAUAUGCCAUGGCAGAAAGAAACAUCAAAGGUUUCACAUCAGUGGUCCAUAUGGUAUACCCCUACAAUAGUGAAGCGAUAGAUCACUUGAUAGAACCACAUGAUAA | 534 |
| pre-miR4 | CCGCUCCAUAAUAAUUGCAACACUUUGACUAAAUAUUUUUGGUCAAAGUGUUAGCAAUUAAUAUGGAGCGGA | 72 |
| pri-miR4  (unigene28393) | CCGCUCCAUAAUAAUUGCAACACUUUGACUAAAUAUUUUUGGUCAAAGUGUUAGCAAUUAAUAUGGAGCGGAGGAAGAAUAGAGUAUAGAUAGUAUUUGAUUUGGAAUUUGUAGGUUGUAUGUAUGUUGAUUGGGGUAUAGUGGUAAUUAUUAAGUAUAUUCGAUCAACGAACUUGUAUAAAUUUUGUAUGUUUUAAUUUAUUGAACAUCAUCAAUAUUACAUACUUUACGUUAAUAAUUAAAUGAUACACCAGC | 255 |
| pre-miR5 | CUCAAUAGUGCUUCCCAUUUCUGACCCUUCAGGAGGGCCUACUGAAGAGGAGGCCGAGAGCAUGUCGGGCGGCUCUUACCUUCCUGGCUACUCCGGGAAGGAGAAGCUGGACGUUGUCCGCGGGAUGCUCAAAGGAGUUCCCAAGGAGGUAGCCCACAACAUGAAGGGCUUUCAUGAUGGGCAUAUUAUGCCC | 193 |
| pri-miR5  (unigene26616) | UGUCAGGUAAAACUAUCUCAUCUUCACCUUCUCACCCAGACUCAAUAGUGCUUCCCAUUUCUGACCCUUCAGGAGGGCCUACUGAAGAGGAGGCCGAGAGCAUGUCGGGCGGCUCUUACCUUCCUGGCUACUCCGGGAAGGAGAAGCUGGACGUUGUCCGCGGGAUGCUCAAAGGAGUUCCCAAGGAGGUAGCCCACAACAUGAAGGGCUUUCAUGAUGGGCAUAUUAUGCCCAUCUUGUACAAU | 245 |
| pre-miR6 | UGGAUGCUACUAGUUCUGCCUUUAUACAUGAACAGUUUCCCCUGCCUAUAUGUUUUGCCUCCUUCUCUGGUUUGUUGGGCAAUGAUGUAGGCCGGCCUGGUUUCUU | 106 |
| pri-miR6  (unigene41537) | GGCCUCCUUUGUUCCUUUGGUGGGAUGUUGGAUGCUACUAGUUCUGCCUUUAUACAUGAACAGUUUCCCCUGCCUAUAUGUUUUGCCUCCUUCUCUGGUUUGUUGGGCAAUGAUGUAGGCCGGCCUGGUUUCUUAUUGUGGUUAUCAUCCCCUAGUUGCUAUUGGAAUGGGUUCGAUGGUGGGAUUUUGCGGAAUUUUCUUUCGCCUUUUGUCCUGCUUUCUCCACCAGGAUGGCUAACGUACUUCCCAGUUUGGCAGCAGGGUUUGUUAACCCCAUGUAGAGCUAGCAUCUUUCCACCUGAGCCCCUAUCUACCUUCCUCUUACACUUUCCCUGAUAUCUUUGCUAUUUUUAAUUAUUGGUUCUUUUGCCUAUGCUCCUUUCCUUUUUUUUUUU | 395 |
| pre-miR7 | CUGUCGUAGAUGGUAGAACAUUCUAGAGUAGAAAAUUCUAGAUUAGUAUAAUCUAGCGUGGAAUGAUCUAGAUUAGAAGAACCUACCUAUAUUAGUAAAGGAGAUGAUACAAUAAUCAUCAUCGAUACUCUAGGAUGUUCAUGUAUUAAUACUAGGAUUC | 160 |
| pri-miR7  (unigene6308) | AGAUUUAGUAAGUUUUUACUCAUUCUGUAAGUUAAUUGGUUUAUUUAGGCCAUGUUCUUUAGAACUGAUAUGAUCUGAUGUGAACCUAUCUGAUUAGGUGGGGUAUUAUUUUGUGAGAGUAUAAUAUUAAAACAAAGCUGAACUGAUCUGAUCUGAUUACUAUAGUUUUAAGUCCAAAAAACAAGGCUUACUGUGCCACGGAAAUUGAAUGUGCGUCGAUAAAUGCGUGUUACAUCUAUCAUCACAAUAAACAACCAAAACUUCAUAAUAAUGUUACCAAAAUUUAUCAAUAAUCCUGUUUGGUUAGAUGUAAGAGGCAGUCUAUGAUUCGACAAUGAAUUGAAGAUGCAAGACUAAAAAAUUAAUACAGUAGUUUACAAACUGGAUCUCAGCGAUCGAUGUUUUUCCAGUUAAAAUAAUUUAUCUUCCUGUUUGUUGGAACUGUUAGGACCUAGGAAAAUUAGAGCACGAGUAGAAGAAAAUUGAUAUUGAGAAAUAAAAUAGAUAAAAAAUGAUGGAAAAAUAGGAAAAAUAACACACAAUAUAACAUGGGAAAAACCCUGGCCUGGCCUCAAAUGAAGUAAUAAACAACCCAUAAACAACGAACACUUAAUUAUCCAAUAUAUGAAAAGGGUAUGAGAGUACAACUCCGAGGGUACAAGAGUAUGGUAUAACUCUCCAAUAACAUAUUGGAUCAAAUAUACAUAGAGAGGUAACAAUCUCCCUAAGGAGUUGGUAAUCUCACAUACAAGUGAAAUAAUAAAAAUGAAUACUCUCUAUGAAUAUAUAAGUAUGUUUGUUUUUCACUCCAUUUAACAAGGGAAAAAAGAGGCGUAUUUAUAAUACUACAAGAGGUAGGCUGUCGUAGAUGGUAGAACAUUCUAGAGUAGAAAAUUCUAGAUUAGUAUAAUCUAGCGUGGAAUGAUCUAGAUUAGAAGAACCUACCUAUAUUAGUAAAGGAGAUGAUACAAUAAUCAUCAUCGAUACUCUAGGAUGUUCAUGUAUUAAUACUAGGAUUCAUAAACCUUGAAGAACAAGGGUCUUGUAGCCCAAGAGUUAACUUGUAGUAGUAAUUUAAUUUGUGACAUUUACCCAACAGGAACAGAUUUACUUGUCUGUUUGUGACUUCAAGAUCGCAAGUUUGAUUCUCACCAGCUACAAGCCUACAACUGAUUUUUUGACCUUCAAUUAUCCUCAAAUUCUUAAGCAUAUGAUUUAAAAUUAAGUCAAGUGACCUCCAGUUGUCGUACGUACUGUUAAAAACUUGAAAAUCCACUCAUAUGCGAAGUAUUGUUUUGUAGUAAAGGCCAU | 1311 |
| pre-miR8 | UCUGAUGUGUUAUUGAUAGAGUAUAUAACAUAUUCCUGGGCUUCAACCAAAAGCUUAAGUUGAUAAUUAAGGCCCGAGAAUAUGUUAUAUACUCUAUCAGUUAUGUGAG | 109 |
| pri-miR8  (unigene15749) | CAUAAUCAUCACCUUGCACAUGAUCUAGGAAUAUUAUUAUGCUAAAAUCCUCUGAUGUGUUAUUGAUAGAGUAUAUAACAUAUUCCUGGGCUUCAACCAAAAGCUUAAGUUGAUAAUUAAGGCCCGAGAAUAUGUUAUAUACUCUAUCAGUUAUGUGAGUGUACAUUGCAUAUACAUACAUUAUUAGUCAGAGCUAGAGGCCUAAAGCUCAUCAGUAGUGUGAAGUGUGAUCCCCUUAAAAUAAAGCAUAAAAAAACCAAGAAAGAUCAGAGAAAUUGAGGAAAAUAAUGGGAAUUUGCUUUAGUAGUAGUAGUAAUAAAGUGAAAAAACAUGCUUGGGAGAAUGAUCCCAAAUGGGUUAUACAAAAAAUGAAGGUGCUUCAACAGGAGAUCAAUGUGGUGUUGAAGCAGAGAGAGGAGGAGGGUGAAGUGUACGAGCGAGAAUUGAUGGAGUUUGCGUGUAGG | 464 |
| pre-miR9 | GUUGGGCUGGACCUGGUCCGCUGGGCUGGUCCUGGUCAUCUGGCUGGACCAGGUCUAUAGUGGAGCAUGGACCCUGU | 77 |
| pri-miR9  (unigene10241) | GUUGGGCUGGACCUGGUCCGCUGGGCUGGUCCUGGUCAUCUGGCUGGACCAGGUCUAUAGUGGAGCAUGGACCCUGUGGGGCCAUGUUAUAUGUAUUGGGCUUAUCUCACAUGUGGUCCUGGUNNNNCCUGGUCCUGGAGUGGGCCUUCCUGUGGUUGUUGCCCUCUUGGUGGAUUUAGGAUUUAGUUGGGCUUAAAUAAUCGUGGGCUGGCCCAAUAGUAGUGGGUGUAUUUUACCCCAUCAAUUUGCCCCCAAGUCCAUAAAUUAUGUGG | 272 |
| pre-miR10a | AGCACUUCCUAAAUUGAUUUUGGCACUCCUCACCUUCUCACAUGAAUAGUAAGGGAGAGGAGUACCAAUAUCAAUUUUAGAAGUGCC | 87 |
| pri-miR10a  (unigene40569) | UUUUAAUUAUUAUUCUUAAAUAUUUUUUUUCCCAAUUUUUGUUAGACUAAUUAUUUAAUAAUAAGGGUAGAGAAAAGAAGAAAAUUCCGCAAGAAUUAUUCUUGUAAAUUUGUAUAUGUAGAUUUAUGCUUGGUUGAUGAAUUGUAUGAAAAUACAAGGUGCCACAAGGAGGGAAAUGAUUUUAGCACUUCCUAAAUUGAUUUUGGCACUCCUCACCUUCUCACAUGAAUAGUAAGGGAGAGGAGUACCAAUAUCAAUUUUAGAAGUGCCAAAAUCAUCACCCAGCAAGAAUGGGUAUUGAGUGCGUGCGUGCUACAAAAUCAAAUGAUGAAGCUGGCAAACCAAAAACACCCCUAGACUGAGUGCUUUAAGGAUGGG | 378 |
| pre-miR10b | GGCACUUCCAAAAUUGAUUUUGGCACUCCUCCCCUCUCACAUGAAUAGCAAGGGGGAGGAGUGCCAAAAUCUAUUUCGAAAGAGUUA | 87 |
| pri-miR10b  (unigene45958) | ACUCAUCCUUAUUGGUGCUUCUCCUAGGUAUGUACUAUAUGUCUUCAUUUUUUCUUAAAUAAAUGUUAAGUUGAACUCGACCUUACACAAACUGUCUGUCACUGGCUAGCACCUGAUAACAUUCUCCUUACAUAUAUAGAGUGUAAUUUAACAUUCACCUUACUGAGAUAACGGGUUAUCCUCAUGUACUAUGUCUUCAUUUUUCUUAUGUUUUAAAAUCGUAAGUUGGAUUGGGCCUUACAUAGACUGUCUCUCACUGACUAGCACAUGAUAGAGACAAAGGGUUAGGGCGAUGAUUUUGGCACUUCCAAAAUUGAUUUUGGCACUCCUCCCCUCUCACAUGAAUAGCAAGGGGGAGGAGUGCCAAAAUCUAUUUCGAAAGAGUUAUCCUCAUGUACUAUGUCUUCUUGUAUGUAUGAUAAUGUGUAAUUGUAGUAUGAUCUUAUCUGUUUUCAACUAUCCUGAUGAAAGAUUCCUCAAUUCUUGGCAGUUUG | 494 |
| pre-miR11 | CAUGUGGUGGUUCUGGUGCUGGUUGUUCGAUUUCCUCAACAACAACAGCCGGGGGCCCCUGAAA | 64 |
| pri-miR11  (unigene18170) | UUUGCUGCUAGGAUAGGGCCUCGAAGGCGGCCGCAAAGUCAAAUUGCGGCUGGGGUGGGAUGGGUUGUGCAUGUGGUGGUUCUGGUGCUGGUUGUUCGAUUUCCUCAACAACAACAGCCGGGGGCCCCUGAAAUAAAUCUGCGUCACGCUGGGCUACGGGUUCCGCGGGGAUUAUGUAUGGGCUAUCCCGAUCAAACCUGGUUA | 204 |
| pre-miR12 | GAAUAUGCAUGGGGGCAGCGACGUUGGCAUACUUGUAUCACGAGCUUGGGUUAGCCAGCAGAAAGAAGUGUAAAAAGUUGGCAGGUUGCUUGACGUUGUUGCAGGCAUGGAUAUAUGAGUAC | 122 |
| pri-miR12 (unigene20787) | GAAUAUGCAUGGGGGCAGCGACGUUGGCAUACUUGUAUCACGAGCUUGGGUUAGCCAGCAGAAAGAAGUGUAAAAAGUUGGCAGGUUGCUUGACGUUGUUGCAGGCAUGGAUAUAUGAGUACUUCCCAGCAUUCAGAGCGCACCGUGGGGAGAAUACCCUGUGCGAUGGUGAGCCUCGGUGUCGUAAAUGGAUUGUUCGUCAGGAGUCUAAACG | 214 |
| pre-miR13 | UACCCGUGCCCCGGUACCGGGGCCACGUCUGCCUAAGCUAUUCAACGUGCACAGCCCCCUAUGAAGGCCCAGCACUGGCCCCGGUGUCGGGGUGACACGGCCCCGGUGCCGGGGUGGGUGC | 121 |
| pri-miR13  (unigene187) | UUUCCAACGGUGGGUCCUUUGCGCGAUUCUGAUUUGAAACGAGGAAGUUAUGCGCAUUCGAAGUUAGGGCUUCAUCCUGUCCAGCACUCACCCCGGUACGGGGUACCCGUGCCCCGGUACCGGGGCCACGUCUGCCUAAGCUAUUCAACGUGCACAGCCCCCUAUGAAGGCCCAGCACUGGCCCCGGUGUCGGGGUGACACGGCCCCGGUGCCGGGGUGGGUGCUGACCAUGCC | 234 |
| pre-miR14 | GAAUUAAGGGGGUUUGAAUUAUCUGCCGACUCAUUCAUUCAAACACUCAGUAGAACGAAACUUUUACAACGAGGUGCUACUGUGAUUGCGUGAAUGAUGCGGGAGAUAGUUUCAUCCUUCUCCUUCUGU | 129 |
| pri-miR14  (unigene26408) | AGAGAGCUUCCUUCAGCCCACUCAUGGAUGGAAUUAAGGGGGUUUGAAUUAUCUGCCGACUCAUUCAUUCAAACACUCAGUAGAACGAAACUUUUACAACGAGGUGCUACUGUGAUUGCGUGAAUGAUGCGGGAGAUAGUUUCAUCCUUCUCCUUCUGUGCUUGGACUGAAGGGAGCUCCCUUUCUCCAUCUAUUUCCUUCAUUCUUGCGCACUUAAUAUGUCAAAUCGUUGCAGUUAAUAUGU | 244 |
| pre-miR15 | GCCGUUGACAUUUUGGAUUUGUAUGAUGCAUCCACAAUUAGAUUUGAGUUUUCAUUUCUUAGGUUCAGGGUGUGAAUAUA | 80 |
| pri-miR15  (unigene30974) | CAGGCAUAUCAUAGUCUAUAGUUGGUUUAUUGAUUGACAAAGGUACACAAAUUGAUUGACAAAAAAUUUAGGCUCUUAAGUGUUAUGAUAGAACAUAAGGAAAUGGAUUUAUGAGAAUAUUAGAUAUAAAGAACCAUAAUGUAGCUAGUUUUGGAUGUGAUUCUAUAUUUUUUUGGUUGGGUGCAGCCGUUGACAUUUUGGAUUUGUAUGAUGCAUCCACAAUUAGAUUUGAGUUUUCAUUUCUUAGGUUCAGGGUGUGAAUAUAGUCUAG | 271 |
| pre-miR16 | UUAUGCGACCCGUUUUACCGAUCGUUUGGUUGGGUGUGUCCUUAGUCGCCCCAGAGAAGCCAUACCCUUAAGAGGUUGAGUCGAAAGACAAAGGAAGUAGAAAAGGCUGGGGCAAAUAUCUAAAUCAUUAAAAUGCAUCAAAACCAAAUUUAUCCUUAAAGUGUGUCAUUCUG | 173 |
| pri-miR16  (unigene15518) | CUGUGGUGAUGACAGAAUUUGCGCCUAUUUGUAGUGGAUAAAACUGGUACAGAUACAGAUUCAGAGGAUCUUGGAGUUUUCUAUGCAAAUAAUAGGGGCGGGACCCUUAUGCGACCCGUUUUACCGAUCGUUUGGUUGGGUGUGUCCUUAGUCGCCCCAGAGAAGCCAUACCCUUAAGAGGUUGAGUCGAAAGACAAAGGAAGUAGAAAAGGCUGGGGCAAAUAUCUAAAUCAUUAAAAUGCAUCAAAACCAAAUUUAUCCUUAAAGUGUGUCAUUCUGAUGGCAACUAUCUAAGGAAAAAAAUUGAAUCAGAUUGCAAAUAUCUAAGGAAAUUUGAAUCAGAUGGCAAGUAUCUAUCAGAUGGCAAAAAACAUUGGGUUUUGAUUUAUUUCUCCCGAAAAGUGUAUGCAAGUAUCUAACGAAAUUGGGUUUUGCCAUCAGAUAGUUGCCAAUUUUGCCAAUUCAAAUUUCCUUAAAUAGUUUUUGAAUCAGAUAGU | 497 |
| pre-miR17 | UAUGUUGUGUUGGUGUUCGAUCCAGGUGGAAUGAGGCGGUGUUCCUACUACGAUGUUGAUAUACGAGUAUCAAUUGAUUGCUAGCUAUAGACUUGAGGGCAAGUCUUUUUGAAGGAGGGAAGUAUGUUAUGGGAGUUUUACUAAGGGUAGUCAAGAGUUAGUUUAAUUUGUUACUAGCUUAAUAAGAUUUGCCUAUAUAUAGCUCAAUUAAAGAUAUGUAAUGGGCAUGAUAAGGAUUUGGAUGAAUAUGAGGAUGAAUUA | 261 |
| pri-miR17  (unigene8872) | CUAAGUUGUUGCUAUCAUGUGUACCACAUUUCAUGGAAAAGUCAAUACCAAAACAACAUUGUUAGGAAACAAGAAAAGGGAUUGCCGGAGUACAAACUAAAAUUUUAAUUCAAAUAAGGCCUCCCUUGCACUCUUUCGAUGAUAUCACAAACUUCAUAUGGUCGGAUUGUUCUACUAUGUGGUUGGAUGUUUAUGUUGUGUUGGUGUUCGAUCCAGGUGGAAUGAGGCGGUGUUCCUACUACGAUGUUGAUAUACGAGUAUCAAUUGAUUGCUAGCUAUAGACUUGAGGGCAAGUCUUUUUGAAGGAGGGAAGUAUGUUAUGGGAGUUUUACUAAGGGUAGUCAAGAGUUAGUUUAAUUUGUUACUAGCUUAAUAAGAUUUGCCUAUAUAUAGCUCAAUUAAAGAUAUGUAAUGGGCAUGAUAAGGAUUUGGAUGAAUAUGAGGAUGAAUUA | 452 |
| pre-miR18 | ACUCCCUCGGUAACAUAUUAAUGGCAACGAUUUGACAUUAAUCGUUGCCAUUAAUAUGUUACGGGAGGGAG | 71 |
| pri-miR18  (unigene9510) | AGGGACUGGCAUAAAUCCUACGCGCUUCUGUAAUUACAUUCAUAUUACAUUUGGUUAGCAACAUAGUUGGUGUAUAGUACUCCCUCCCGUAACAUAUUAAUGGCAACGAUUAAUGUCAAAUCGUUGCCAUUAAUAUGUUACCGAGGGAGUAUGUUUUGUAGCGAUACAGAUAAAGAAGAUGUUACUUUUUAUGCAUUAGAUGUAUAUUAUACAACGGAUUAAAAUUUGCCCUAAAACUCAUAUAUAAG | 248 |
| pre-miR19 | CCGCCCUAUCGGGUUGGCCAACCCGAUCGGGUCAGCUCCCACCCGACCGGUCGGGUCACCCAACCCGAUCGGGUUGA | 77 |
| pri-miR19 (unigene16665) | GAUCGGGUCAAGUGUCAUGUGACCGGGUGAAGUCACCAGAUCGGGUGCGAAGCAUCAUGUAGAAGAAUUGGAGUUGUCCCGCCCUAUCGGGUUGGCCAACCCGAUCGGGUCAGCUCCCACCCGACCGGUCGGGUCACCCAACCCGAUCGGGUUGACGAGUGAUUUUUUUUUGCCAGUUUCUCUUCUUCUCUUGGACGGUUAUAUAUUAACCACUACUUCUCCAAUAAACCCUAAUACAUUCAUACACACUUUCACCCUAUUGUUUUCACUUCAUUGUAGAGAGAGUUUUUUGAGUGAUUUACACAAAUUGUUCAUCUUUCUCGUUGUGCUAAUUUAGCCCCUUUGAGGAAUUCUUGUAUACUCUUUCUCCAUUAUAGUGAAAUCUUUGCUCGUCGCCGUUGGUGGGGUUUUUUCCCGUUUUGGGUUUCCCCACGUUAAAUUCUUGUGUGUUCUUUAUUUUAUUGUUUAUUUAUUCUUGCCAAAUUUAUCUGUUUCCGCUGCGAUUGUAGACAUUAAUCCCUAACAGGUAUUGUAAAUGGAAUUAUGACUCAUCGCUGCUAUUUAACUCCAUAAUUGAUGUUAUUGCUUCAAUUUUUCUAUUUAUUUGCUCAAUGUCGUUAUUGUUUGAUGGAUCCUAUUUAGGAUGAAUCCUAAUUCAGAGUUUGUCGUUGUUGUAGUCUGAUAGGAUCCUAAUUAGGAUUCAUCCUUAUUNNNNNCAAUUAACAACGAUGAACUUUUUUUAUUGAAGUUAGCUGGUUAACUUCAAUUUAUUUAUUUUGAUUUUCUUGAAUUUGAUAACAUUUAGUAUUAUUUUUC | 816 |
| pre-miR20 | CAACUAAAAUUGGAAAUUGUUGCUAGUAAUUCAUUACAGAGGGAGUAGUUUUUUGAUCUGUACAAGACAUAGUGGCAGACAACGUUCUGAGUUAUUUAU | 99 |
| pri-miR20  (unigene1776) | AAAGUAAGAGAGAGAAAAGUAAGAGAAGAAAAAGUAAAUGAGGUAUUAUGUUGUAUGAUGAUAAAAGUAAGUCAGAAAAGUAAGUGGUGUAUUGUGUUGUUUNNNNNNAAAAAAAUAAAUAAAAUGAAAUUAGAAAAAAUUGUUGCAAGUAUUUUGUUACAACUAAAAUUGGAAAUUGUUGCUAGUAAUUCAUUACAGAGGGAGUAGUUUUUUGAUCUGUACAAGACAUAGUGGCAGACAACGUUCUGAGUUAUUUAUGUCGUUUGUUAUAAUUUGGAAAGUAUUGAAUAGAAUAAUAUAAAAAGGCAACUUGAUUUGGAAAUAAUAGUAAGGGC | 335 |
| pre-miR21 | ACGUUGUGAUGACCGGCAUGACCCAAUUGUUGAUCACAUGCACAAUAUAUCUAUUUUGUGUUUUCGGAUUAAUUCCUAAUUAAUUCAUAUACCUUGGGAUGUUUUGAAUUUGGAUCAUCUUCAUUUACUACUUCCGUUCCAAAAAAAUGCAACAAUUGUCAUGACACGGACUCCUAUGCAU | 181 |
| pri-miR21  (unigene31871) | CUUUUAAGUAUUUAGGCUAAAAUUGAACUGUUGACCUGAGAUUUAGCCUGAUUUAAGGACAGGUAACACCCAACGUUGUGAUGACCGGCAUGACCCAAUUGUUGAUCACAUGCACAAUAUAUCUAUUUUGUGUUUUCGGAUUAAUUCCUAAUUAAUUCAUAUACCUUGGGAUGUUUUGAAUUUGGAUCAUCUUCAUUUACUACUUCCGUUCCAAAAAAAUGCAACAAUUGUCAUGACACGGACUCCUAUGCAUUUUUUUGUUGUGUGUAAUUAGAAAA | 278 |
| pre-miR22 | ACAUUUAUGAUAAGCAUUUAGAACAUUCAUAUUUUAGCUCGUAUUUGAAUAUGAAUGUUCAAAAUGUUUAACAUAAAUGUU | 81 |
| pri-miR22 (unigene47986) | AUUGUGUUAUGCAUUUAGAACAUUUAUGAUAAGCAUUUAGAACAUUCAUAUUUUAGCUCGUAUUUGAAUAUGAAUGUUCAAAAUGUUUAACAUAAAUGUUUAUAAAAAUUAUAUUUGGAAGACAUGUGUAUGAUGAUCACCAUGUACCUGGUUGCACAAAGAAAUUUUCAAGUAAAAAGAGAGUUUUUGGGUAUUUCAAAUCUCCCAGUAAAAUUGGAUGUGAUGCUACUACAUCUGUCCUUCACAAAACAUAAUGACAAUGUUUUUGCUGCUCCAAGAACGCUGAAGCGUGGUUCCUUGUUGAUGACAAUAUACAUCGGUGUCCUGAUGCUGAUCGCUUAGAGCCAGAGGAGUAAACGAGAACCAAGUGUAUAUCGCCUCUUUCGAAAAUACAAGUAUAUGAAAAGCUUACUGUUAUACUUACAUCACUAAUAAUCCAAUGCAGCUGUGUUAUCUUGGAACUUAUAUAAAUGUAACCAUCAAGUUUAAUGCAGCCAUACUUGUAGAUGUACCCUAAUAUAGUGACUGAGGAUGAUUGAUUGCUUGGUGUAAUAUGAAAUAAAU | 564 |
| pre-miR23 | AUAAUGUUGGGUCGAGUGACAAGAAAAGUAAAGCAAGUUGCUCUUGUUUUGACUCAAUAGCCACUUGAAAUGGCAUUUUCCCUUCACGAUUCUUUAUUUUCCAUGGAGUGAAGUAAUUAGUUGCCUCAUUAGAGAUCUCACUGGCUGAUGAUGUUACUUCUUUUUGGUACUCACGUACAACAAUUAUCCCUAUCUCUUCAUUACUAUUUCUUGCUGCAAUGUGUAAUGGAGUGUCACCCCUUUCCUUUUCAGUCGUCUGACAAACUAAAUUC | 272 |
| pri-miR23  (unigene285) | AUAAUGUUGGGUCGAGUGACAAGAAAAGUAAAGCAAGUUGCUCUUGUUUUGACUCAAUAGCCACUUGAAAUGGCAUUUUCCCUUCACGAUUCUUUAUUUUCCAUGGAGUGAAGUAAUUAGUUGCCUCAUUAGAGAUCUCACUGGCUGAUGAUGUUACUUCUUUUUGGUACUCACGUACAACAAUUAUCCCUAUCUCUUCAUUACUAUUUCUUGCUGCAAUGUGUAAUGGAGUGUCACCCCUUUCCUUUUCAGUCGUCUGACAAACUAAAUUCUUGUAUAUUUUUGUCUUUUCUUCAGGAAAUUCAUCAAGCAACUCUAAUACCCAAAGUGAAUCCAUUUCCAGCCAUUUGUGAAGCACAUUACAUCCAUUGUCAUCCACACAAUCGAUCAGCUCUGGGUGCUUCGACA | 408 |
| pre-miR24 | UUUGAAAUACUACCUCUUGUCCUUAUUAUGCGCAACAAAGGGGUUUAUUUUUGUGAAGGAGAAUAUAUUCCUUUGUUGCACAUAAUAAGGACGGUAGUAUUGUAUU | 106 |
| pri-miR24  (unigene40905) | AUUUUUUUUACUAAAUUUAUUCAUUUACUUGCUUAAAUCUCUUCCUAGAUAUGUAUGUGUGACAAAGACGAAUGUUUUGACAAAUAUGAAGAGUAGGAAGUAAUUGUAUUUUUAAGAGAAAUUGGUCAUAAUAGAAACACACAUGUAGCAAUGAUAAUGGAACAUAUCUUGCAAACACUAGCUAAUAUAAAGUAAUUUGAAAUACUACCUCUUGUCCUUAUUAUGCGCAACAAAGGGGUUUAUUUUUGUGAAGGAGAAUAUAUUCCUUUGUUGCACAUAAUAAGGACGGUAGUAUUGUAUUCAACCUCUACCAUUAAUCUACUAAACUAAACGACAUUAUAAUUACUAGUUUCGUUACUUAUGAUCAAUAUCAUUUGUUAUAU | 383 |
| pre-miR25 | AAUUAUGAUUUAGUAUUUGUUAGUUAGUGGGUCAUGUUUUCACCUUCUCUGUAUUAAUUUUGUGCUUGUAUUGUCUCUAUAAAAACUUCCUAUUUAUUAAAUUAAUACAAUAAAAGGUUAAAUCAUCUUUUUCCUUUAAUAAAACUAUCACCGACCCAGAAUUAAUUAUGUAUCUAAAUUUGGAUC | 186 |
| pri-miR25 (unigene41357) | CUUAACAAGAGCCCAAUUAACAAAGGUUGCACGAACAAUCAUAGCUAGCUACAACAAUUUAGUUCCGAUUGGAACUUAGCCUGAAUCCACAAGUAAAAAAAUCCAAUUCAACUUUUAAAUUGUAAAAUCGACAUCUAACUACUGCCUGAGUGUUGAGUUGAGUUAGUGGGUAAUGGGUCAAAUUAUGAUUUAGUAUUUGUUAGUUAGUGGGUCAUGUUUUCACCUUCUCUGUAUUAAUUUUGUGCUUGUAUUGUCUCUAUAAAAACUUCCUAUUUAUUAAAUUAAUACAAUAAAAGGUUAAAUCAUCUUUUUCCUUUAAUAAAACUAUCACCGACCCAGAAUUAAUUAUGUAUCUAAAUUUGGAUCCGAGUUCGACCCAUAAGACUUUAUU | 391 |
| pre-miR26 | UAGAAUUUCAUUUGAAAUUCUGGAAUUGAGACAAUUAUAAUGUUUGGAUGUCCAAGAAUUUCAAAUGAUGAAUUUGCCCAAAUUCUAC | 88 |
| pri-miR26 (unigene43266) | UAGAAUUUCAUUUGAAAUUCUGGAAUUGAGACAAUUAUAAUGUUUGGAUGUCCAAGAAUUUCAAAUGAUGAAUUUGCCCAAAUUCUACAACUAACAAGUAAUAAGUAAUGAAGGUAAGGAUUUCAAAUGACAAGGUAAACCAUGUCAUUUGAAAUACUUAAAUUCUAUAAUUCAAAUGAUGUAUUUGAAAUGAAAUCUAUUUUCCCAAACACAACCUAAUAUUAUUUAAUUGAUCUGUUAGUAGUGGGUUGUCAACCAGGUGGCCGAGUUGAUGUAGCAAUGUCCUGUUUUUUCGCUAUUCACAAGCAAUGAUGACGUGAUGUUCCUGAGAUUUUGUGUAGGAAAAUACUCCGUAUAUGAGUAUUUCAAGACUAGCUACUAAUUAAGGUUAUUUUUUGUGGCGUAACAAAAGAAUAAUUUAGGAUUAAG | 429 |
| pre-miR27 | ACUUAUUUGCAUGUGUUUGGGCGAAAAUAGGGUGUUUUGGAACAAAACAAGGUAUAUAGGUCAUUUUUGGCUAGAUUUUUGUUUUCGUUCGCAUGUUCCUACCG | 104 |
| pri-miR27 (unigene4541) | GGCAAGGGAUGGGCUCACAAGCUCGGCUUUAAUGGAGGAAAGACACCACAAAAAUCACGCCCAACUCACAGUAGAAGAAAUAGAGAUUUUAGAGAGAUGAAUCAGUAAAAAAAGAAUUGCCAUAGAGAAAGAAAACUAAAAUGACGGCUCUUUCUUUUUCUCUCUCUCUUUCUUUAAUGAAUCUAAAACCCUACAACAGUUCGUACCGAUAACGUGUUACCCUAAACCCAUAACGUUUCUAGAGUAUAAUUAGGGGUAUAAUGGUCAUCCACUAAUAUAUAUUAACAAUGUUAUGAAAGUGGGGAAAAGUGUAUCUCAUUUCAUUCAGUAAAGGAGUAUAUAUAGUAUUUACAACAAACCCUAA | 364 |
| pre-miR28 | UACCGUAUCCCACUGAUGAUGAGGGGAGACCGAUGUUGGCCCCGUUGGGCUCUGUUGAUGAUAGGGGGCGUCGAAC | 76 |
| pri-miR28 (unigene42178) | AUGGCUUUGACUCGACCUCGUUUUACAGUCUUAUUACUCAUGGUUUGGUAUGGCAUGGUCGGGUUAAGAGUUCGUCAUUACCCCACCCGGCACUUCGUUUGGUUCACCGGUUUCUUUCUUUGACCAUUUUUUGUCAAGGUGAACCAACGAACUUGUCGAGGUUUGAGCUGAAAUGUUUAUGGUCUUUGACUCCAUGUGGGUUGGGGUGCCCAGAUUGGGUAGGCAUUUUUGUGGAGAAUUCUUUGGAGUUGGGUAGGAAAGACCGGGGGAAGAUAAGUAUGGGGGGUAUGGUUACCAUUUUGGGGAGAAAUCUAGAGGUACCGUAUCCCACUGAUGAUGAGGGGAGACCGAUGUUGGCCCCGUUGGGCUCUGUUGAUGAUAGGGGGCGUCGAACAUCUUAUGUCUACU | 408 |
| pre-miR29 | GUCCACCAGCCGCUUUAGUUCUUCAGAGUUAUCUCGAGGGUCCUCCUCUGUAGGCGUCACCUUGAGUAUGGAAUGAUCUGGUUCAGGUUCAUGCAAAACUCCUGCUUCUUUUGUUGUAUUAGAGGUUUUAAAAGGUGAAUUCUGCUGAGACUCUAAAUUUAGGCCAAGAUGUGAAACUUGAGGAACAGGCAGGCAUAUAUCC | 202 |
| pri-miR29  (unigene50169) | UGGUCUUUCCUUUACUUAAACCCAUAUCUUUCAGAGUAUUCUCAAGCUGUUCAACAGAAAUCUUAACCAACUCGUUGUUAUAGAAGUCUCCAGCACAACCGUUGUGGAUGAUAACAACAGGAGGCCACAAUAUCAGAUCCUCCUUCAAAGUGUGAGCCUCAACAUCAGGCAAAGGUUUGCAAAUCCAUCGACCAUUAGAUGAAACAGUUGGAUCCCAUCCCAUCAAGACACAUAAAGCUCUAUGAAAUCCCAAAUGUGGAGCUCUAAACCCAACUUUCAGAGAUGUGAAUGCAUGUGUGGCAACAUCUUUUGUUCGGACAAACUCUUUAAAACUGUAACAUAUGAUGCACUUCAAUGUACCAGCAUUUCCUUGCUCCUUGAAUUUUUUCUGCCGGUCAAGAUUUUCAUUCAAGUGAAGCAUGCACUUUGAAAACCAGUGGUCCACCAGCCGCUUUAGUUCUUCAGAGUUAUCUCGAGGGUCCUCCUCUGUAGGCGUCACCUUGAGUAUGGAAUGAUCUGGUUCAGGUUCAUGCAAAACUCCUGCUUCUUUUGUUGUAUUAGAGGUUUUAAAAGGUGAAUUCUGCUGAGACUCUAAAUUUAGGCCAAGAUGUGAAACUUGAGGAACAGGCAGGCAUAUAUCCAUAUGAGGUGCUGCCUUAAGCCUC | 665 |
| pre-miR30 | ACCAAAAAUUUAAGCUGAUAGUUGUGGCCCACGAAUAUGUUAUAUACUCUAUCAGUUUUAGACUCUCUGUUGUACCAUUUUCUUACAUAUUUGUCACAGUGCUUCCAGUGAUUUAUAAGUCAAUUGUUAGGCUUAUUGCUAUCCAAUUAAUGUAAAUUUUACAUCAUUUGGUGGAGAUAUUAGCAUUGACUAGGAUGCGGGAUUGGAGAGAUAUAUCAAUCACGCAGAACAUAUGACACAUAGCUAUUGUUAGCAUUACAAUGUGGA | 267 |
| pri-miR30  (unigene50384) | UGGUCUUUCCUUUACUUAAACCCAUAUCUUUCAGAGUAUUCUCAAGCUGUUCAACAGAAAUCUUAACCAACUCGUUGUUAUAGAAGUCUCCAGCACAACCGUUGUGGAUGAUAACAACAGGAGGCCACAAUAUCAGAUCCUCCUUCAAAGUGUGAGCCUCAACAUCAGGCAAAGGUUUGCAAAUCCAUCGACCAUUAGAUGAAACAGUUGGAUCCCAUCCCAUCAAGACACAUAAAGCUCUAUGAAAUCCCAAAUGUGGAGCUCUAAACCCAACUUUCAGAGAUGUGAAUGCAUGUGUGGCAACAUCUUUUGUUCGGACAAACUCUUUAAAACUGUAACAUAUGAUGCACUUCAAUGUACCAGCAUUUCCUUGCUCCUUGAAUUUUUUCUGCCGGUCAAGAUUUUCAUUCAAGUGAAGCAUGCACUUUGAAAACCAGUGGUCCACCAGCCGCUUUAGUUCUUCAGAGUUAUCUCGAGGGUCCUCCUCUGUAGGCGUCACCUUGAGUAUGGAAUGAUCUGGUUCAGGUUCAUGCAAAACUCCUGCUUCUUUUGUUGUAUUAGAGGUUUUAAAAGGUGAAUUCUGCUGAGACUCUAAAUUUAGGCCAAGAUGUGAAACUUGAGGAACAGGCAGGCAUAUAUCCAUAUGAGGUGCUGCCUUAAGCCUC | 677 |
