# Supplementary material for: High-throughput deep sequencing reveals that microRNAs play important roles in salt tolerance of euhalophyte Salicornia europaea
Source: BMC Plant Biol. 2015 Feb 26;15:63. doi: 10.1186/s12870-015-0451-3 (PMC4349674; doi:10.1186/s12870-015-0451-3)
Supplement: Additional file: 7. — The hairpin structures of S. europaea novel miRNAs predicted by MFOLD. The mature miRNAs were highlighted in yellow while miRNA star sequences were highlighted in green. [file 12870_2015_451_MOESM7_ESM.pdf]

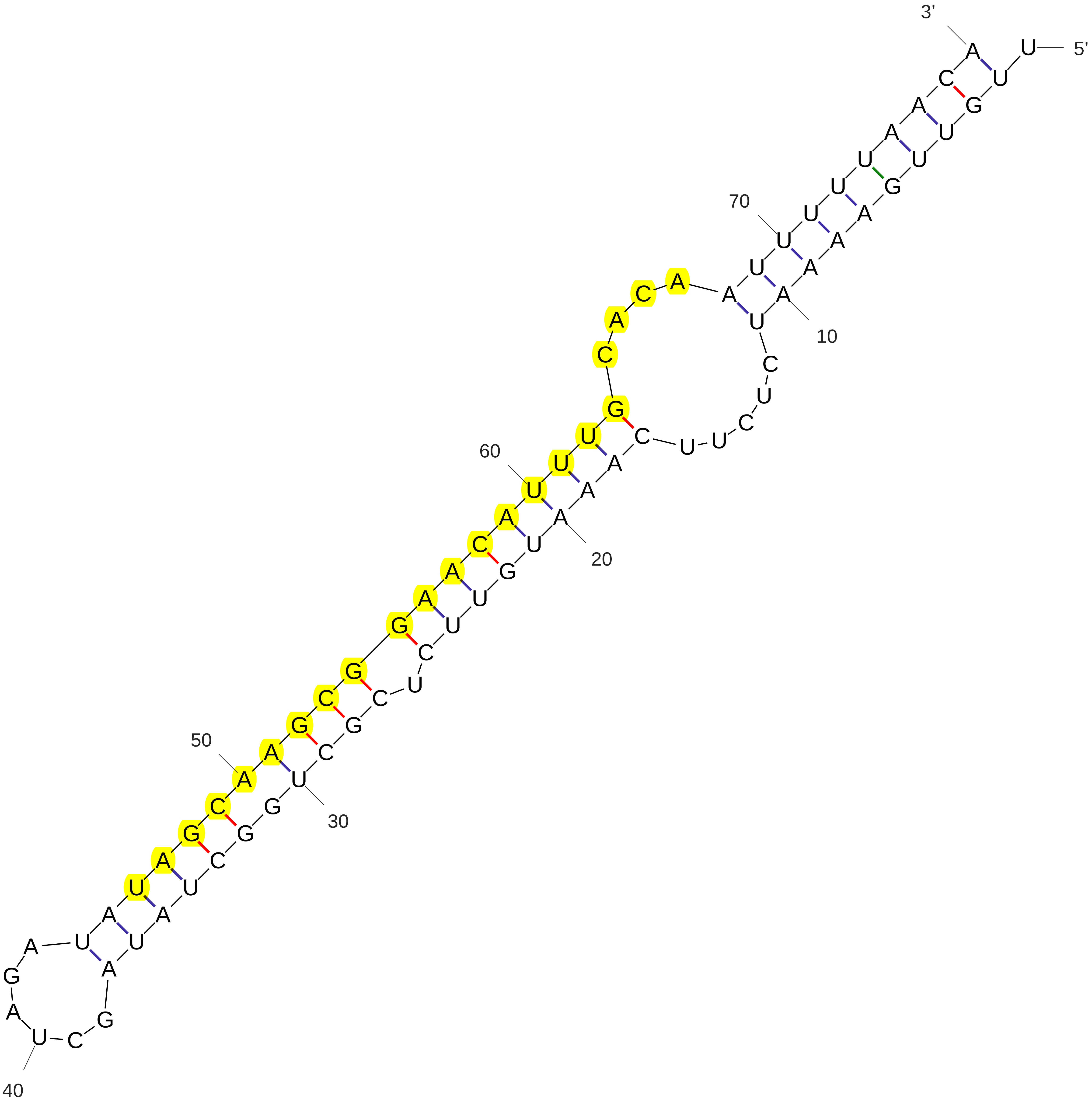

dG = -31.20 [Initially -31.20] Seu-miR1

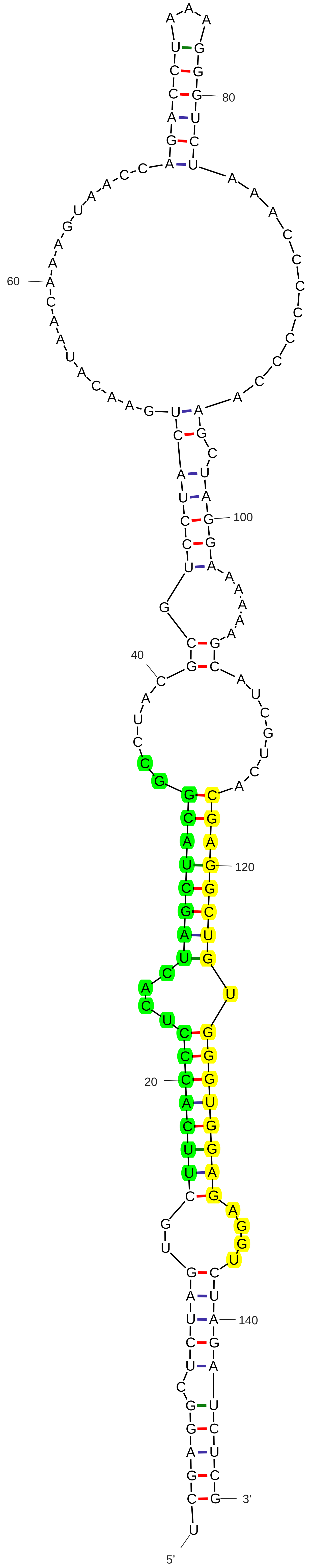

dG = -38.20 Seu-miR2

Output of sir_graph (©)
mfold_util 4.6

Created Mon Jan 13 02:26:50 2014

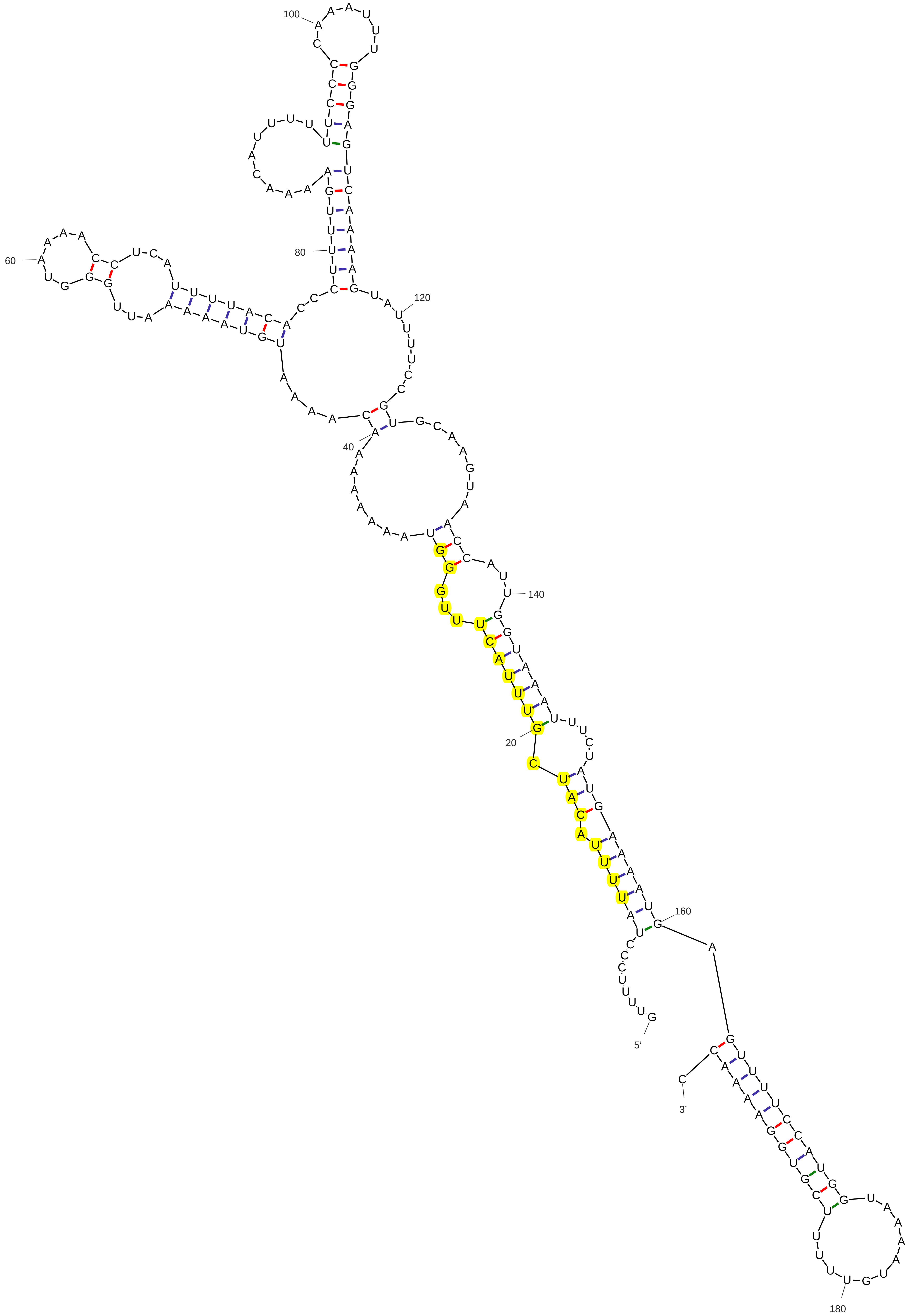

dG = -37.17 [Initially -40.20] Seu-miR3

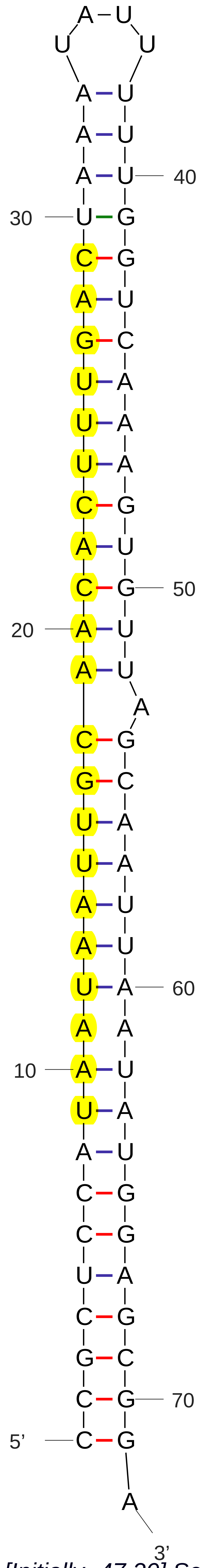

dG = -47.20 [Initially -47.20] Seu-miR4

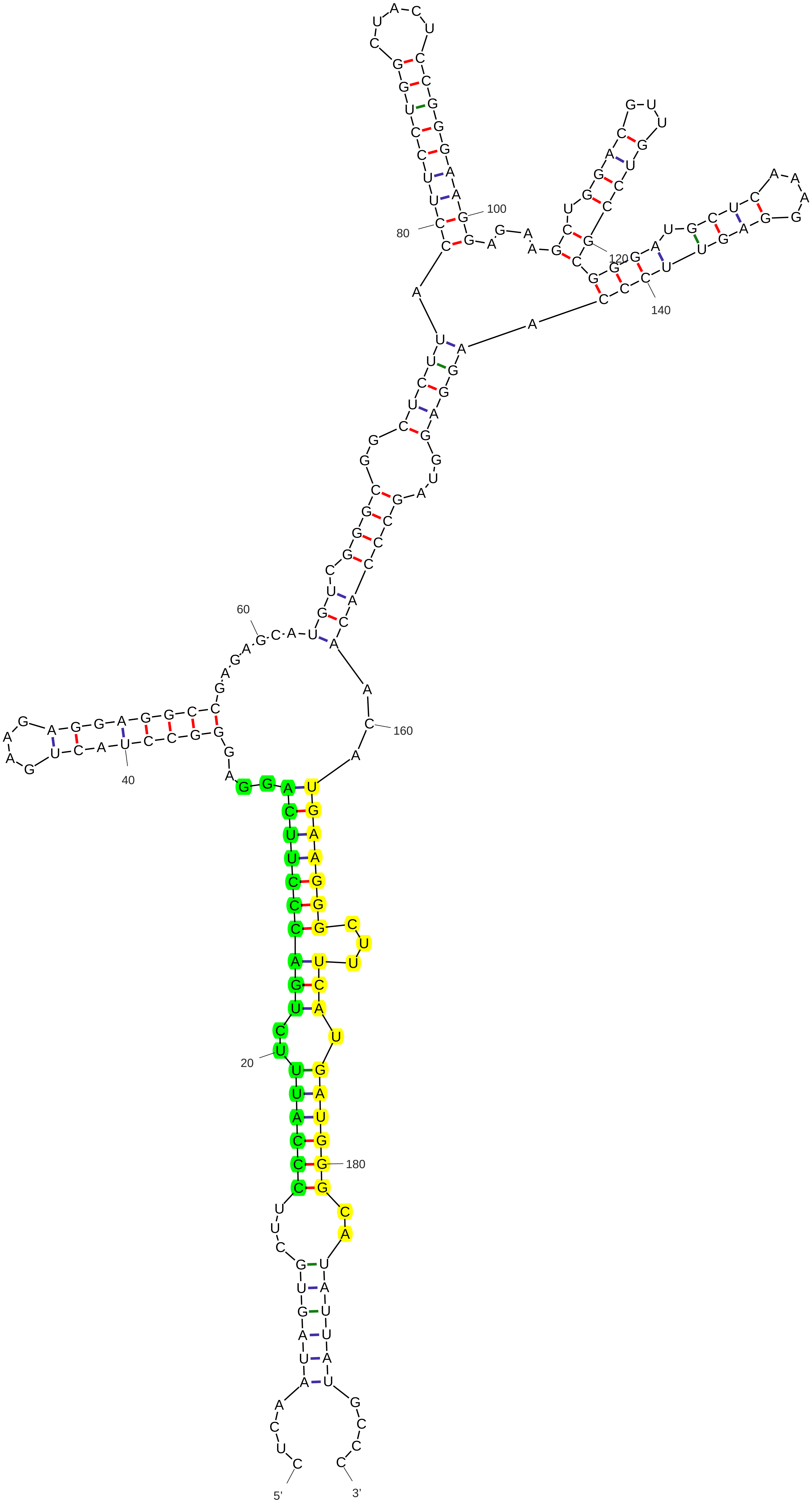

dG = -70.87 [Initially -75.50] Seu-miR5

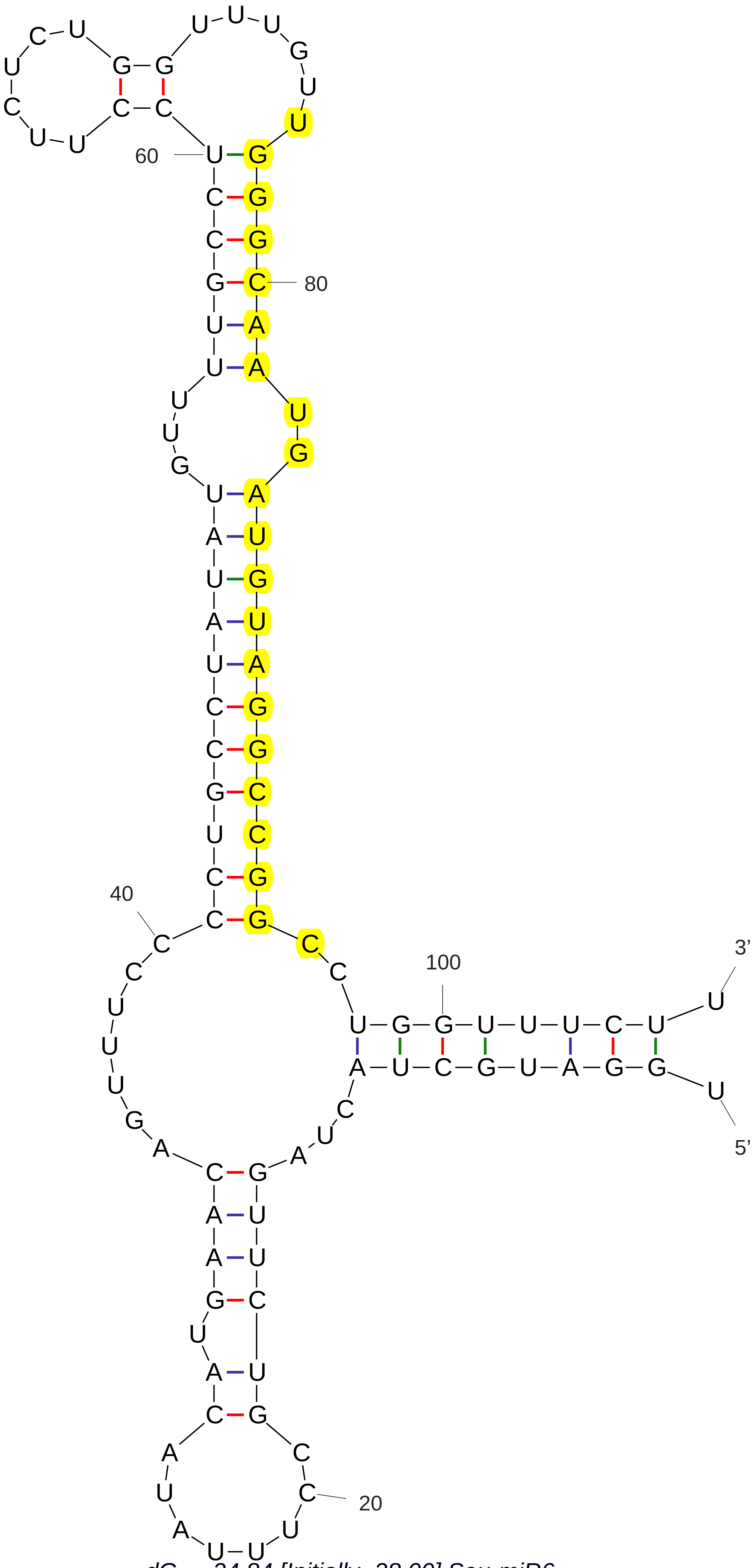

dG = -24.84 [Initially -28.00] Seu-miR6

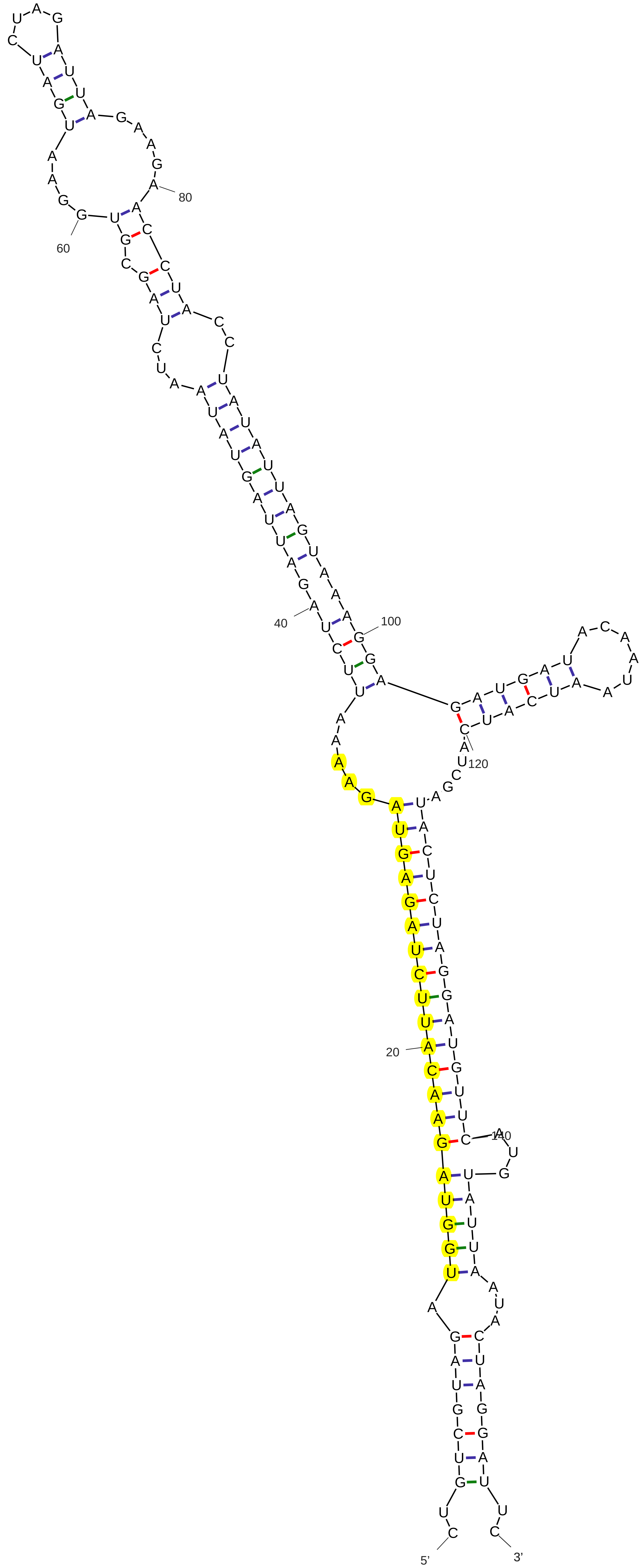

dG = -39.24 [Initially -42.30] Seu-miR7

Output of sir_graph (©)
mfold_util 4.6

Created Mon Jan 13 02:42:50 2014

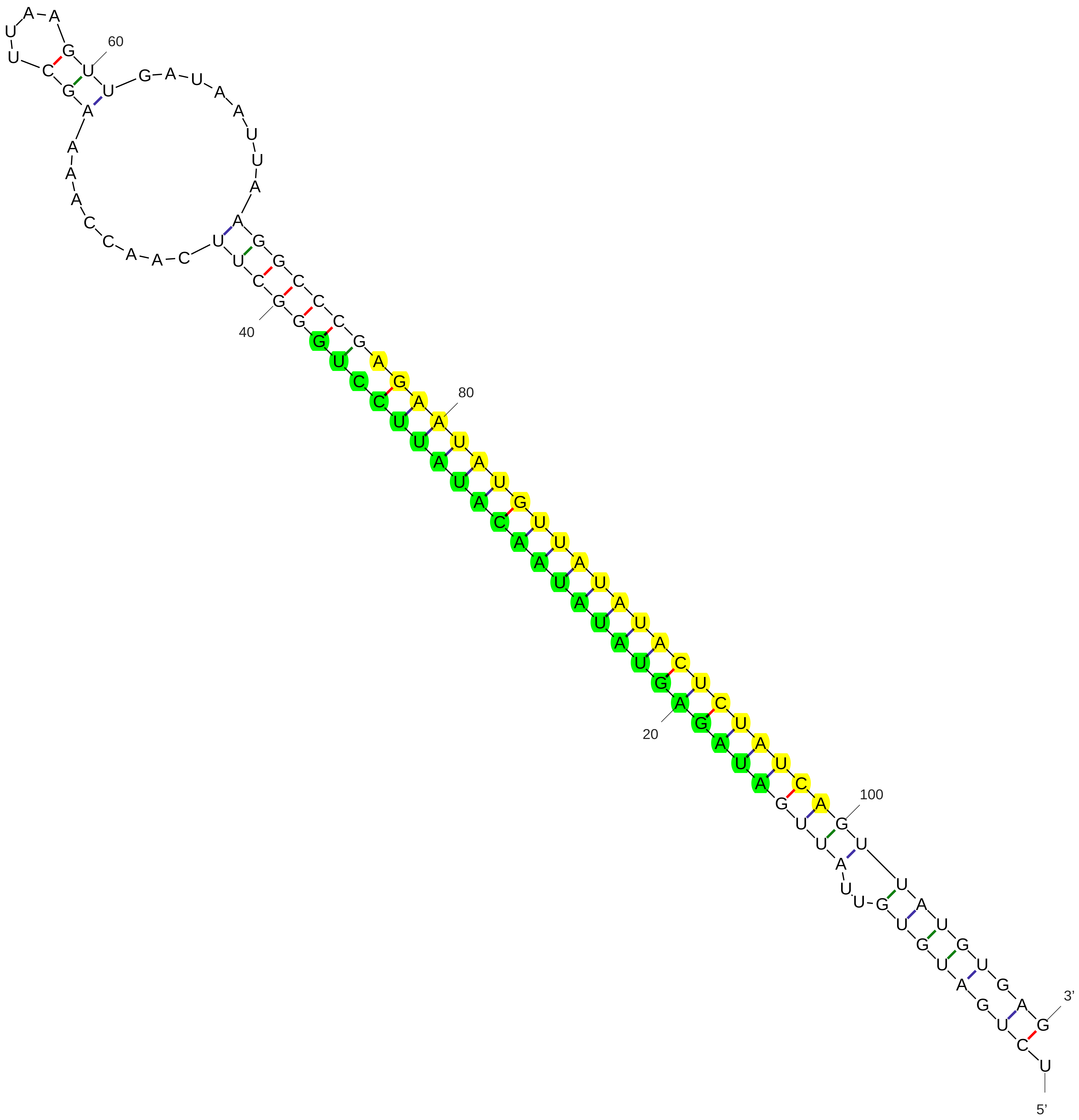

dG = -47.00 [Initially -47.00] Seu-miR8

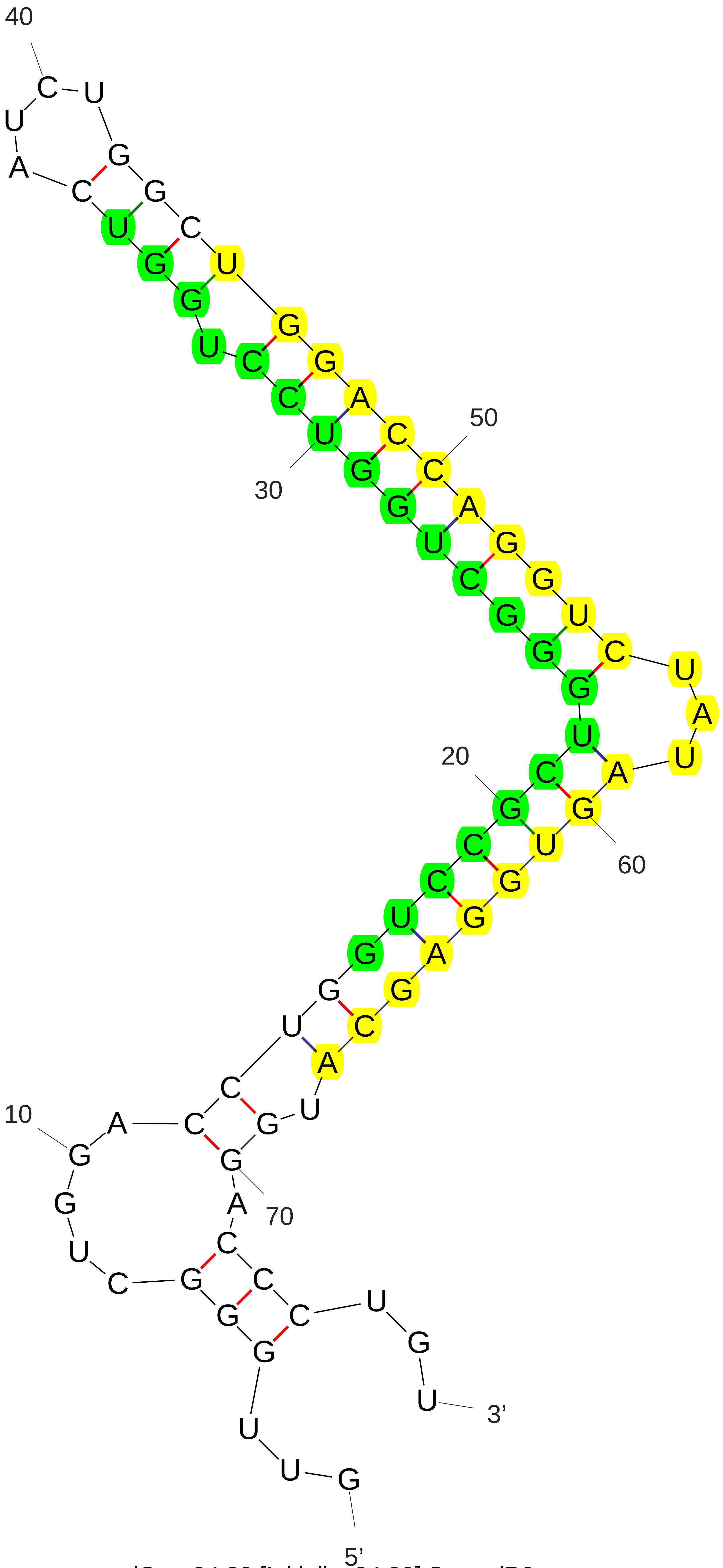

dG = -34.30 [Initially -34.30] Seu-miR9

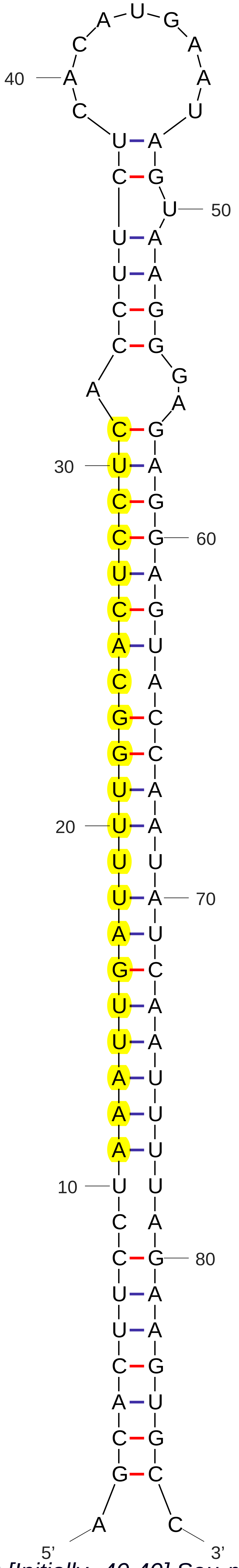

dG = -40.40 [Initially -40.40] Seu-miR10a

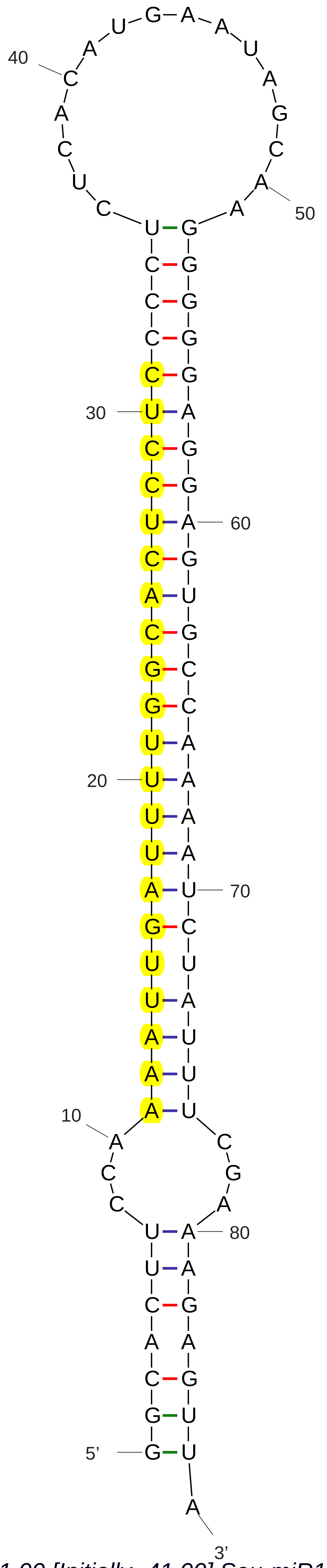

dG = -41.00 [Initially -41.00] Seu-miR10b

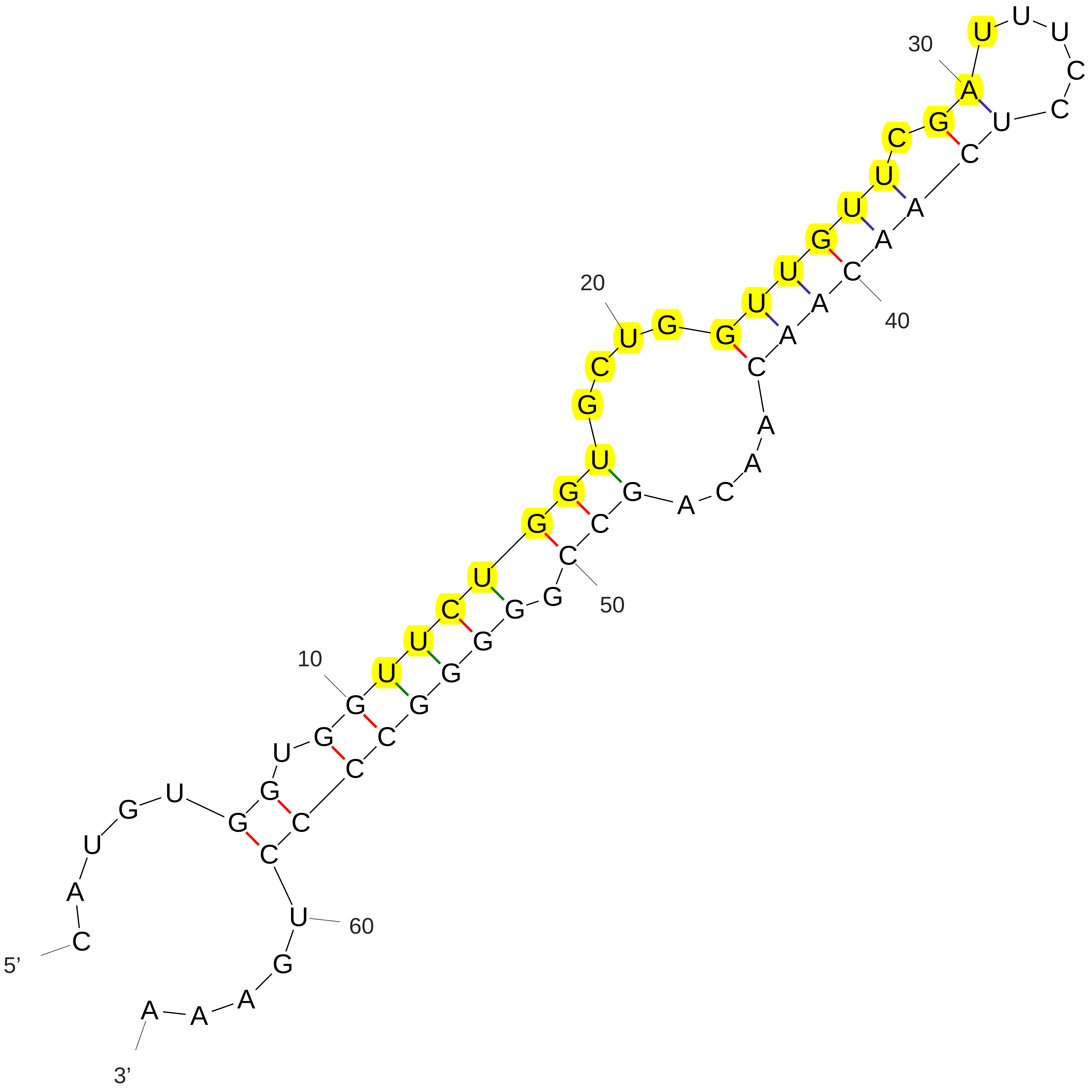

dG = -20.20 [Initially -20.20] Seu-miR11

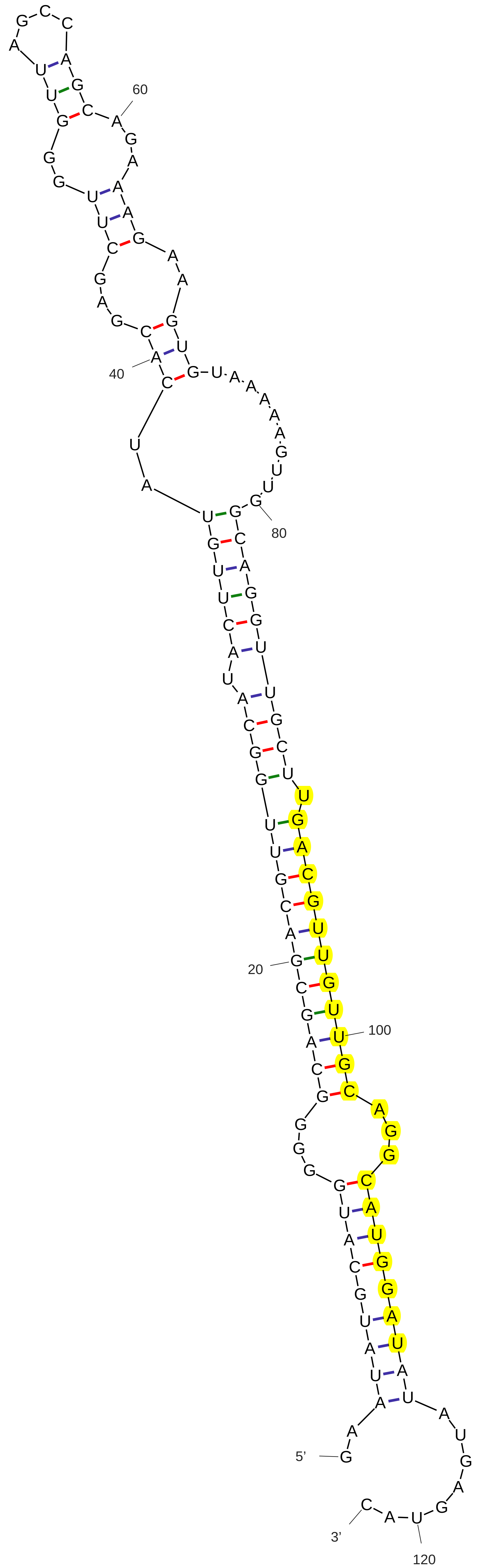

dG = -38.50 [Initially -38.50] Seu-miR12

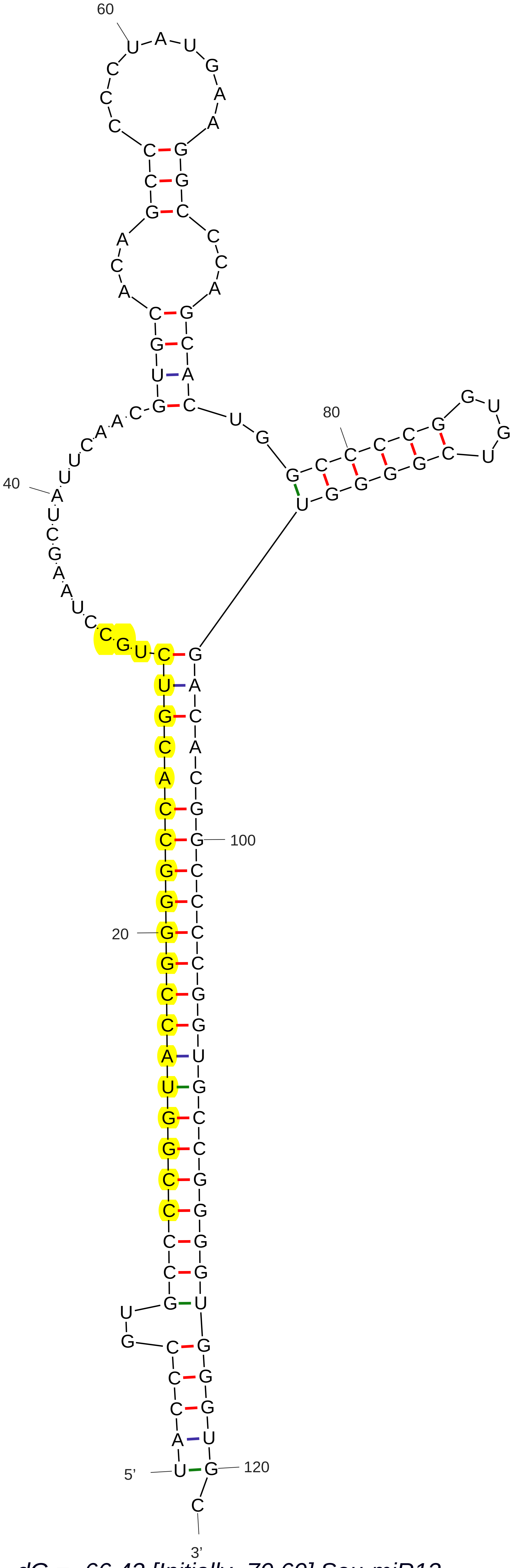

dG = -66.43 [Initially -70.60] Seu-miR13

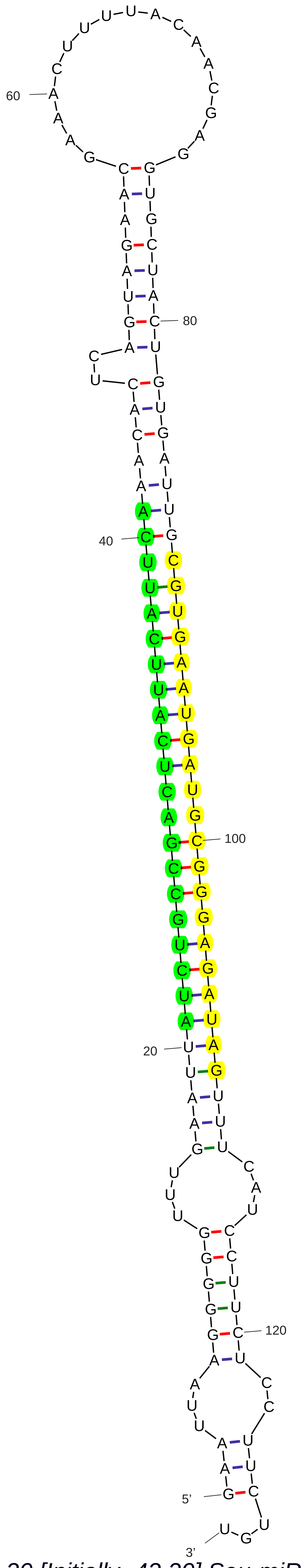

dG = -42.20 [Initially -42.20] Seu-miR14

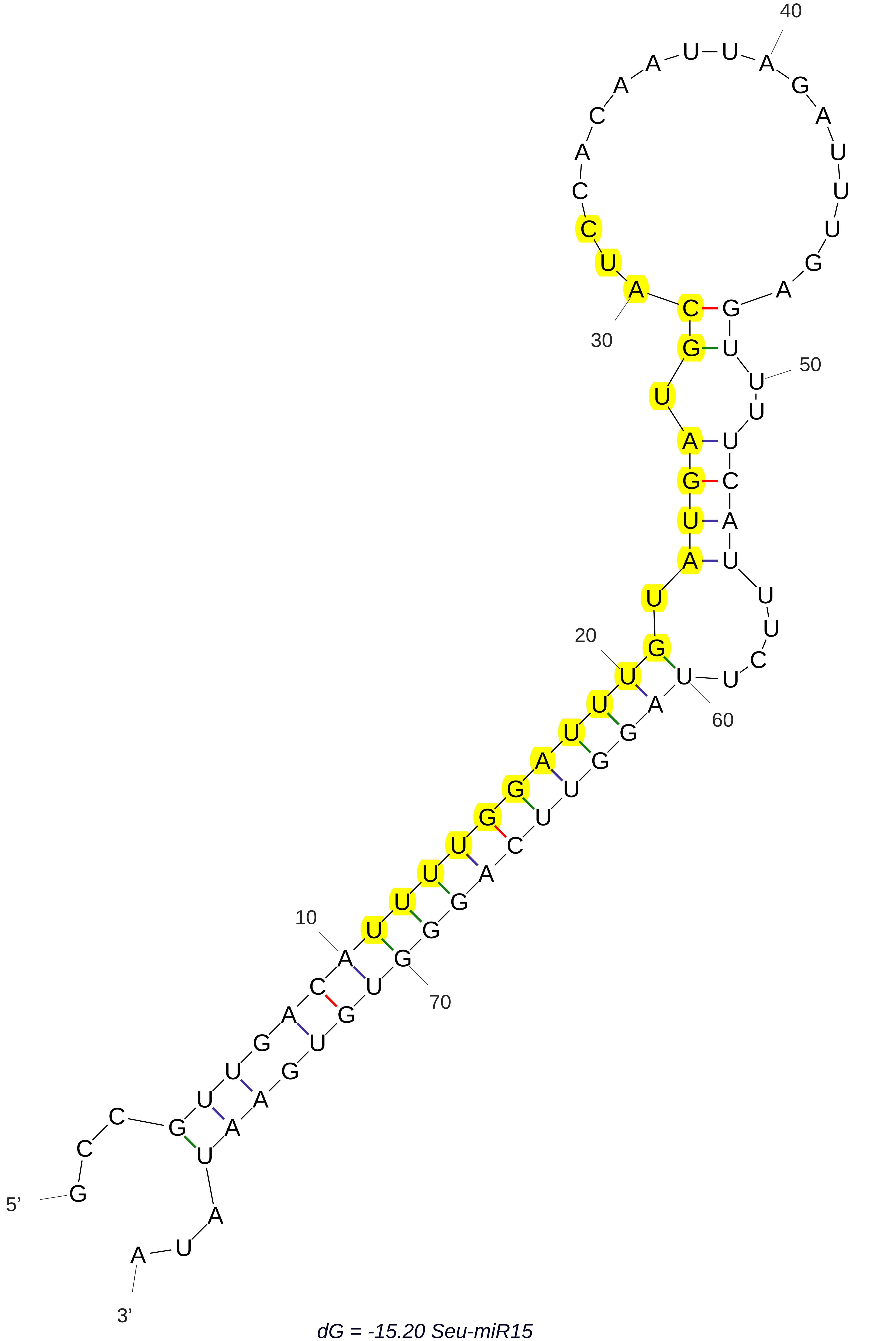

dG = -15.20 Seu-miR15

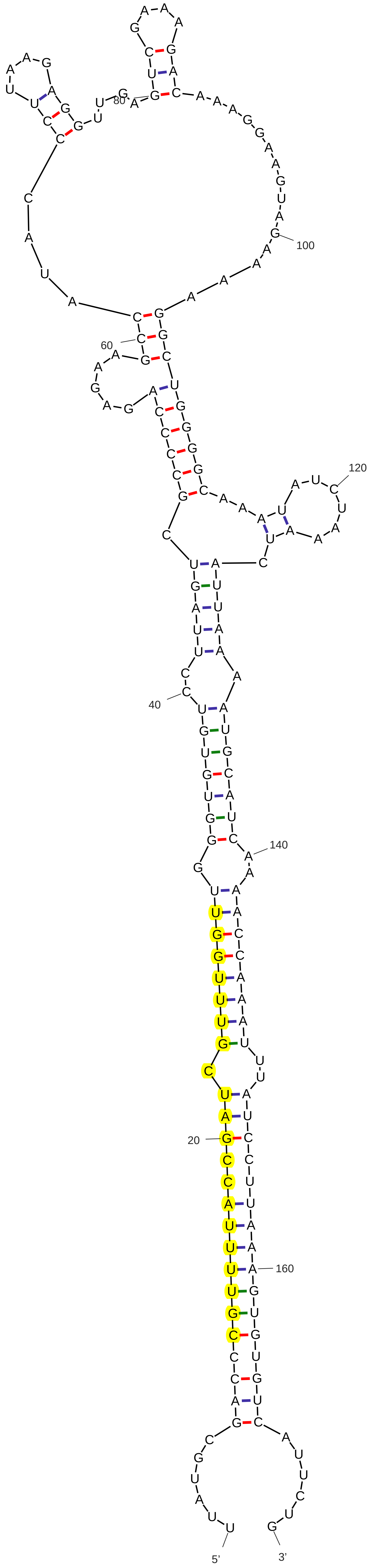

dG = -37.62 [Initially -42.00] Seu-miR16

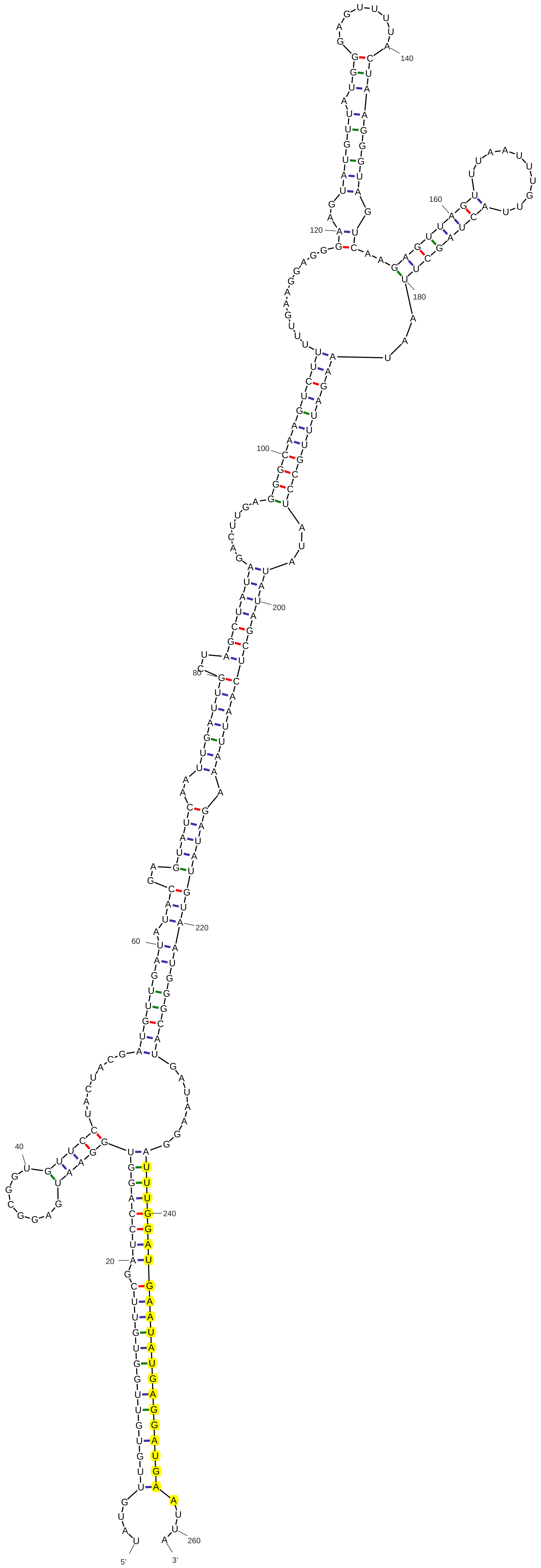

dG = -57.89 [Initially -63.70] Seu-miR17

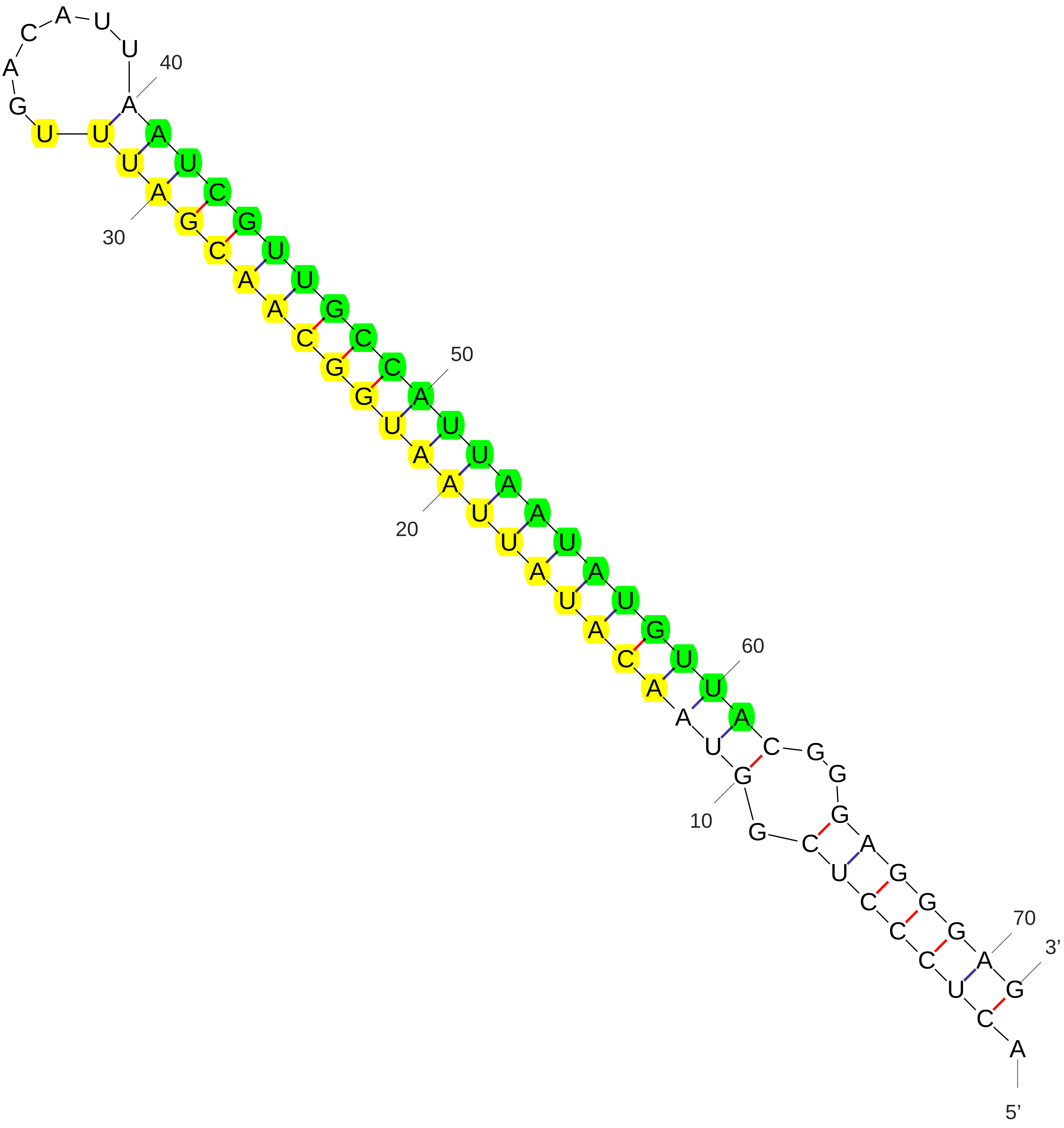

dG = -46.00 [Initially -46.00] Seu-miR18

Output of sir_graph (©)
mfold_util 4.6

Created Mon Jan 13 21:19:57 2014

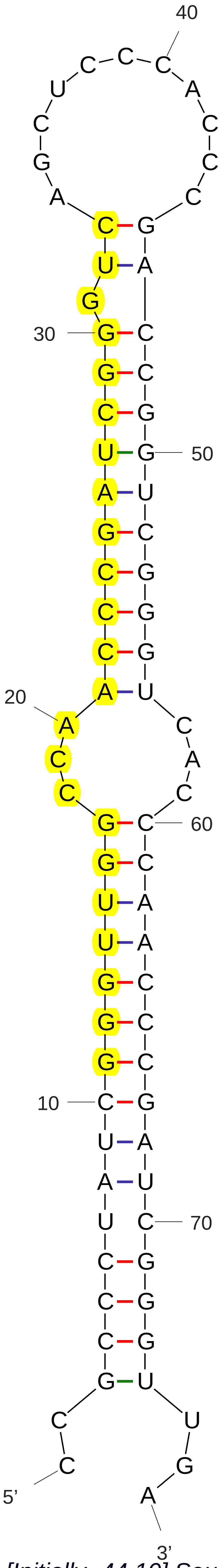

dG = -44.10 [Initially -44.10] Seu-miR19

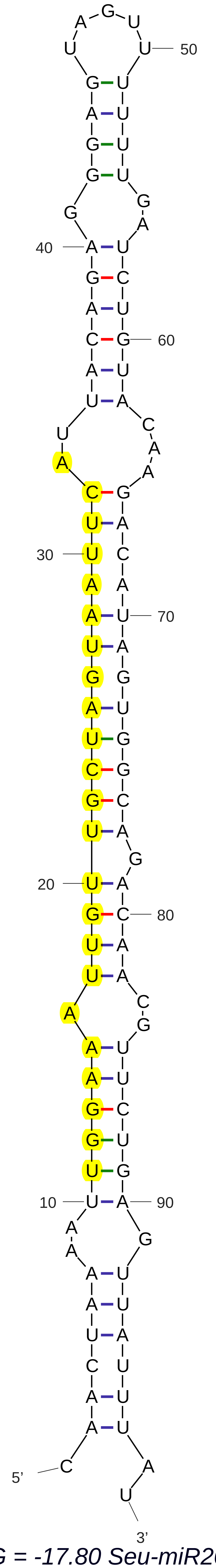

dG = -17.80 Seu-miR20

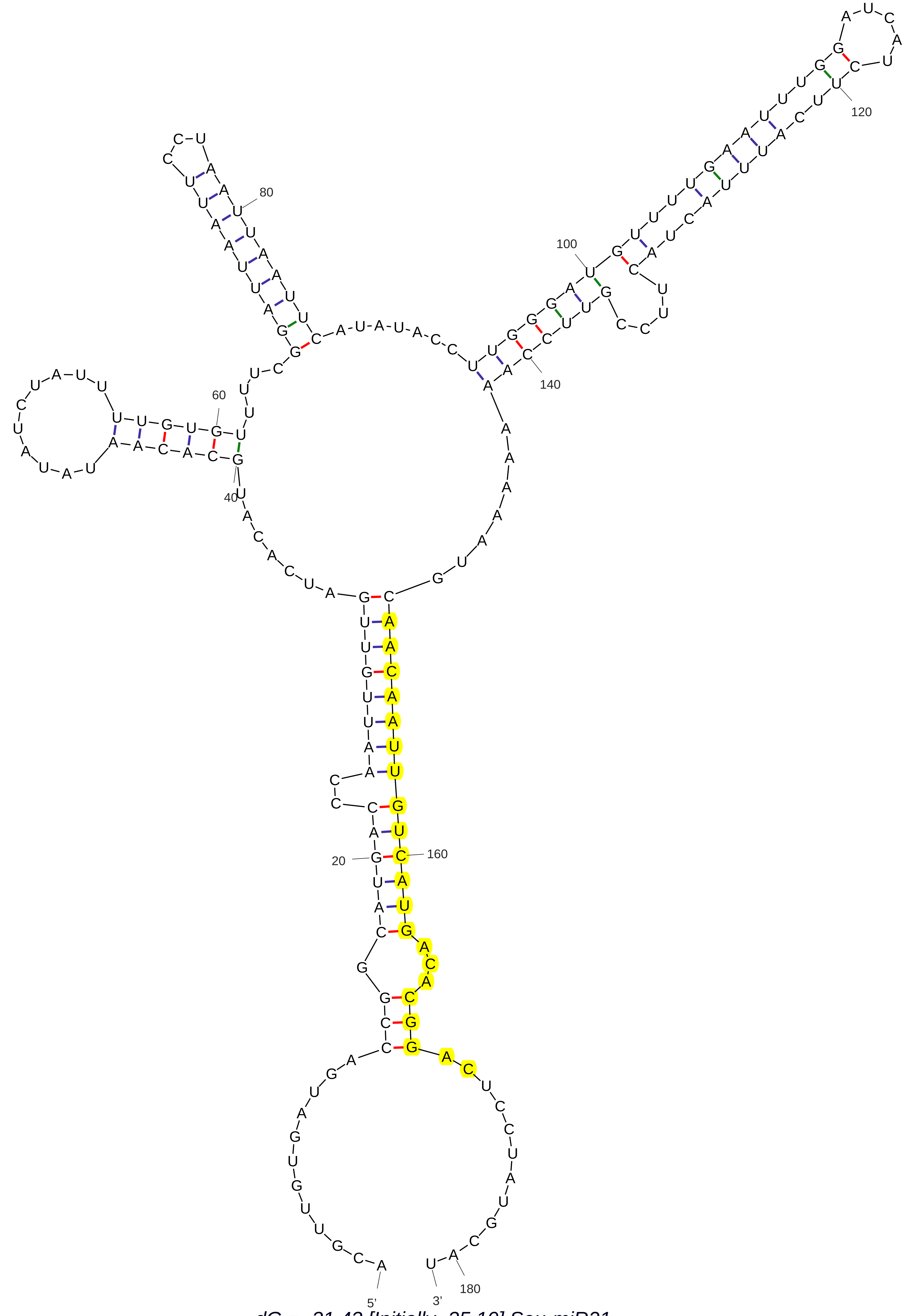

dG = -31.43 [Initially -35.10] Seu-miR21

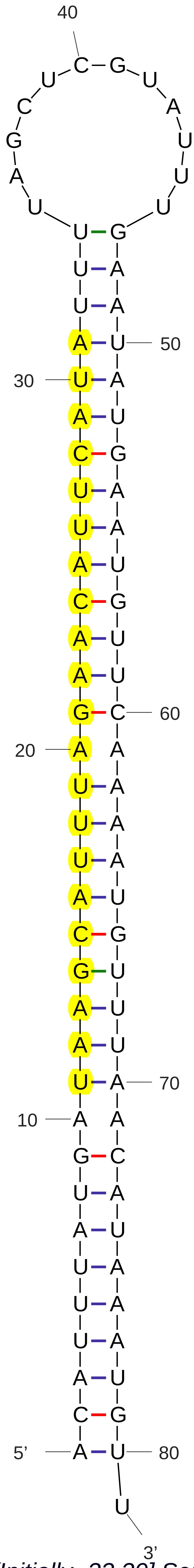

dG = -33.30 [Initially -33.30] Seu-miR22

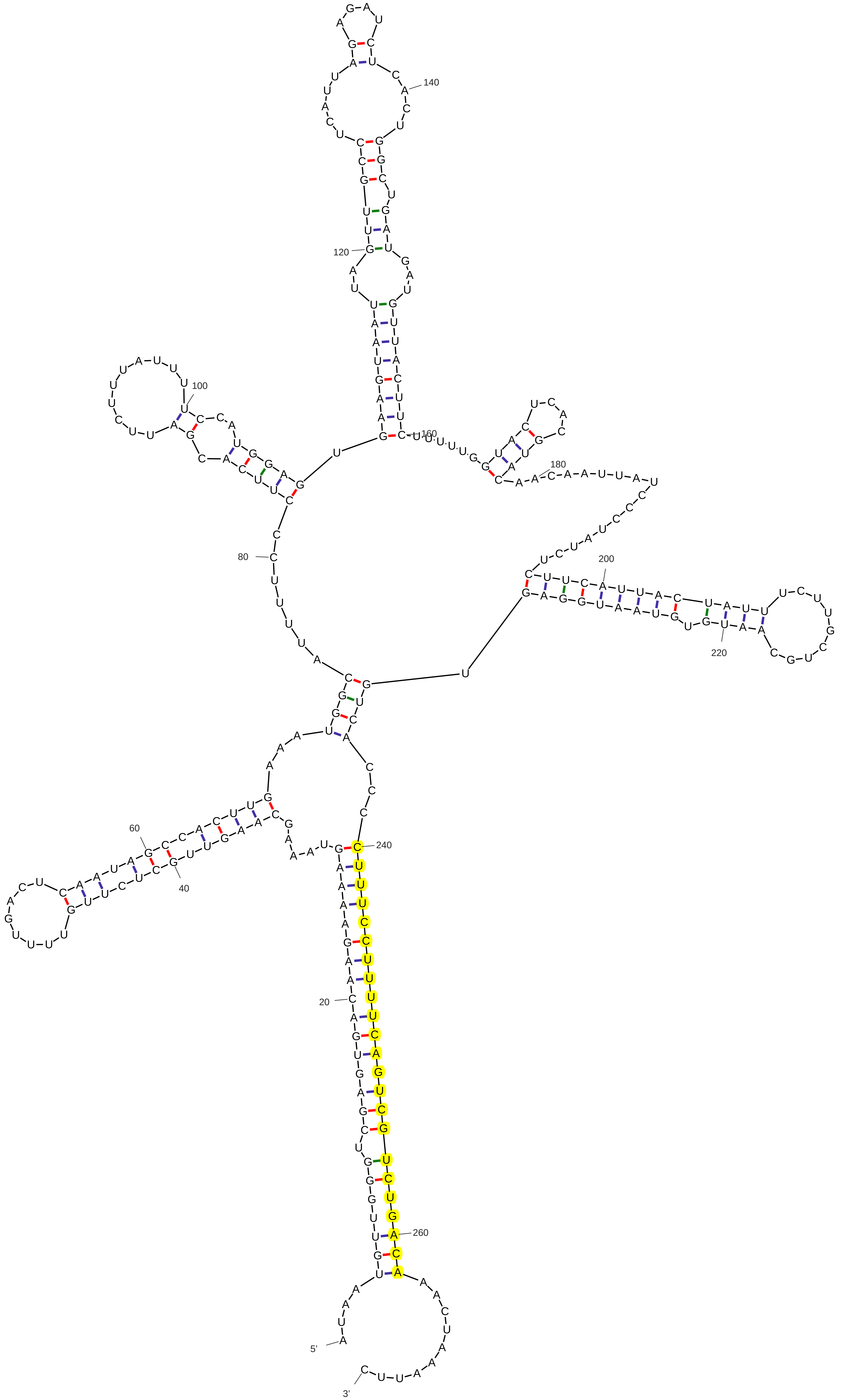

dG = -51.59 [Initially -58.10] Seu-miR23

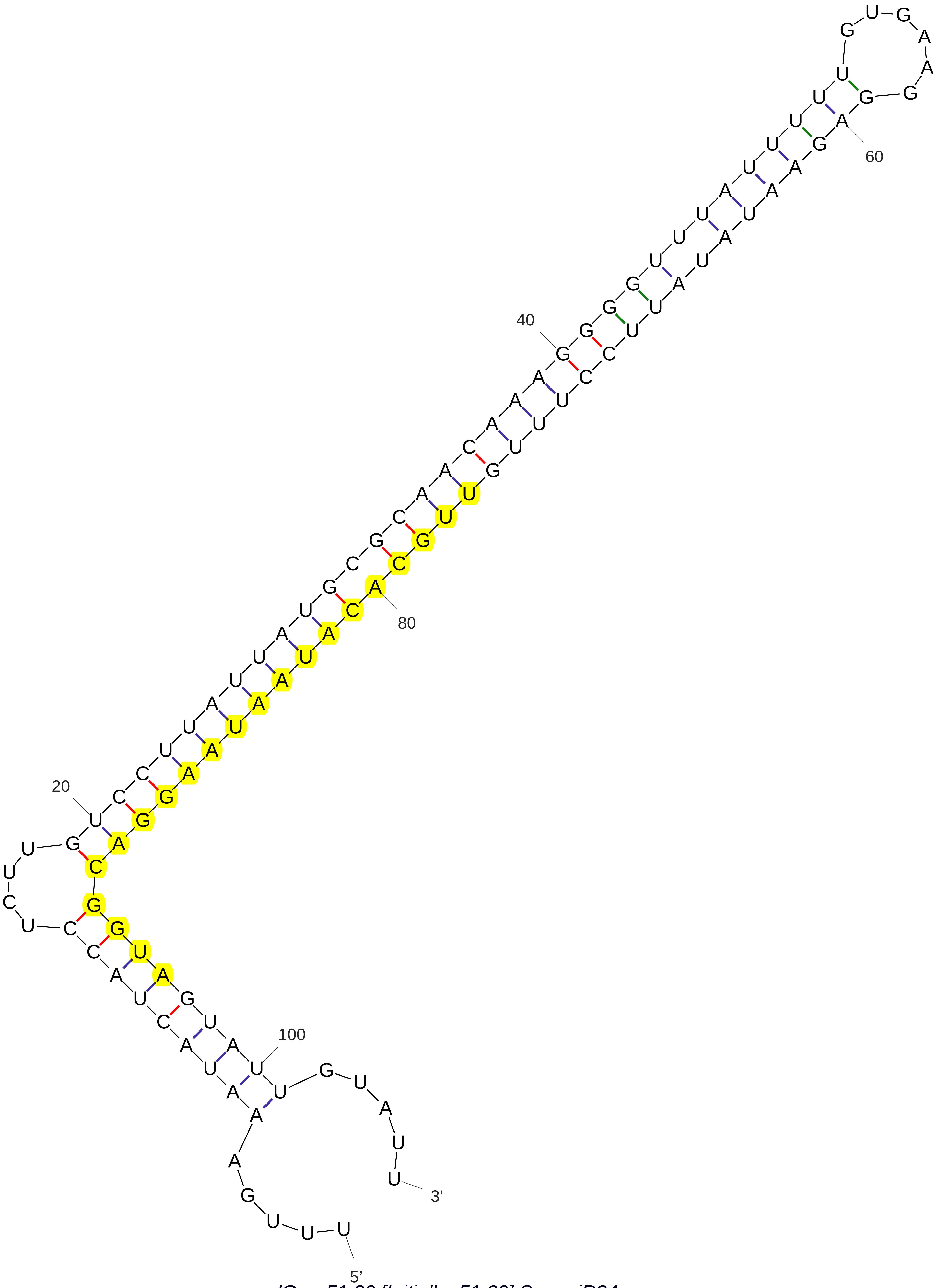

dG = -51.30 [Initially -51.60] Seu-miR24

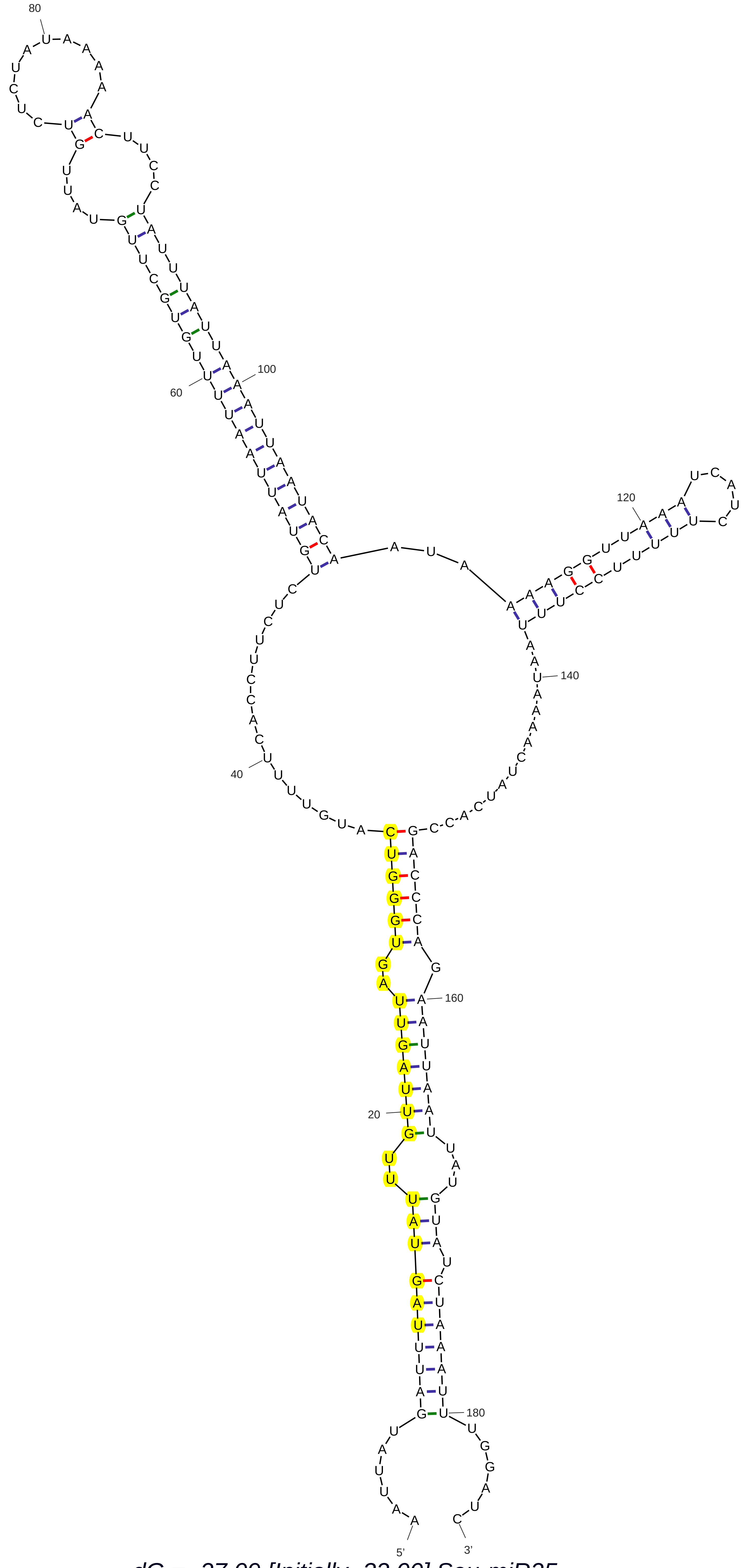

dG = -27.09 [Initially -32.00] Seu-miR25

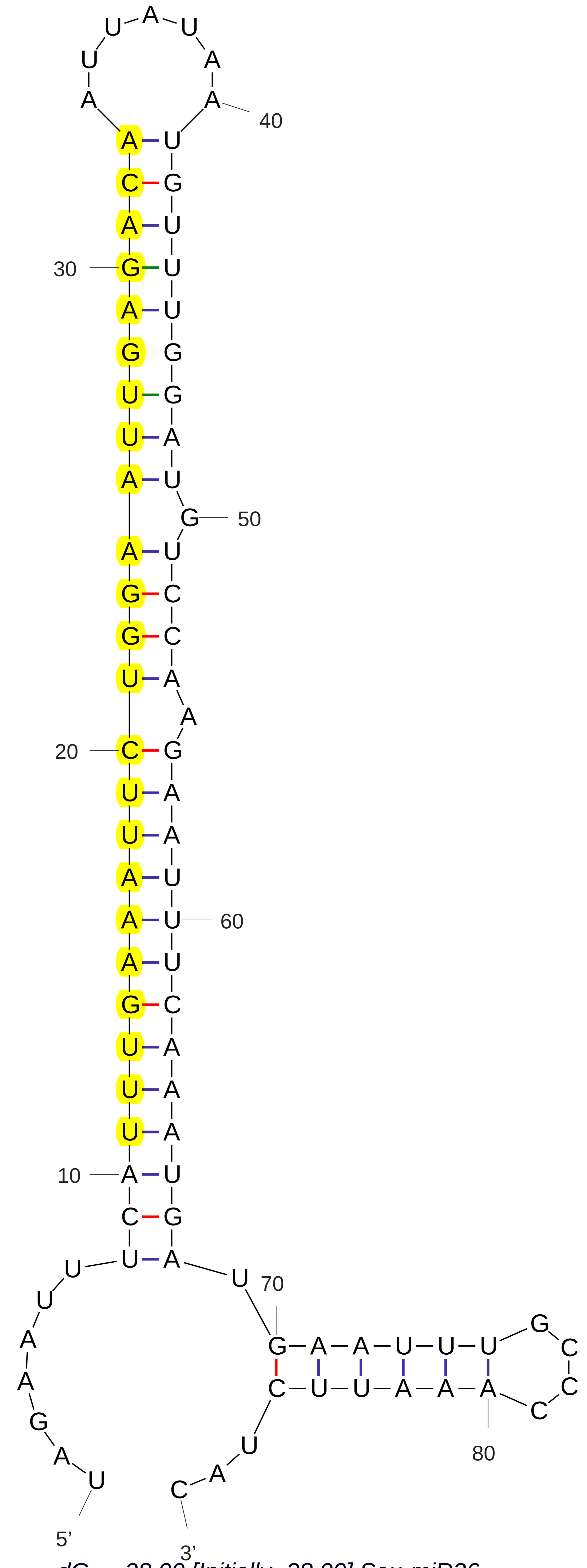

dG = -28.00 [Initially -28.00] Seu-miR26

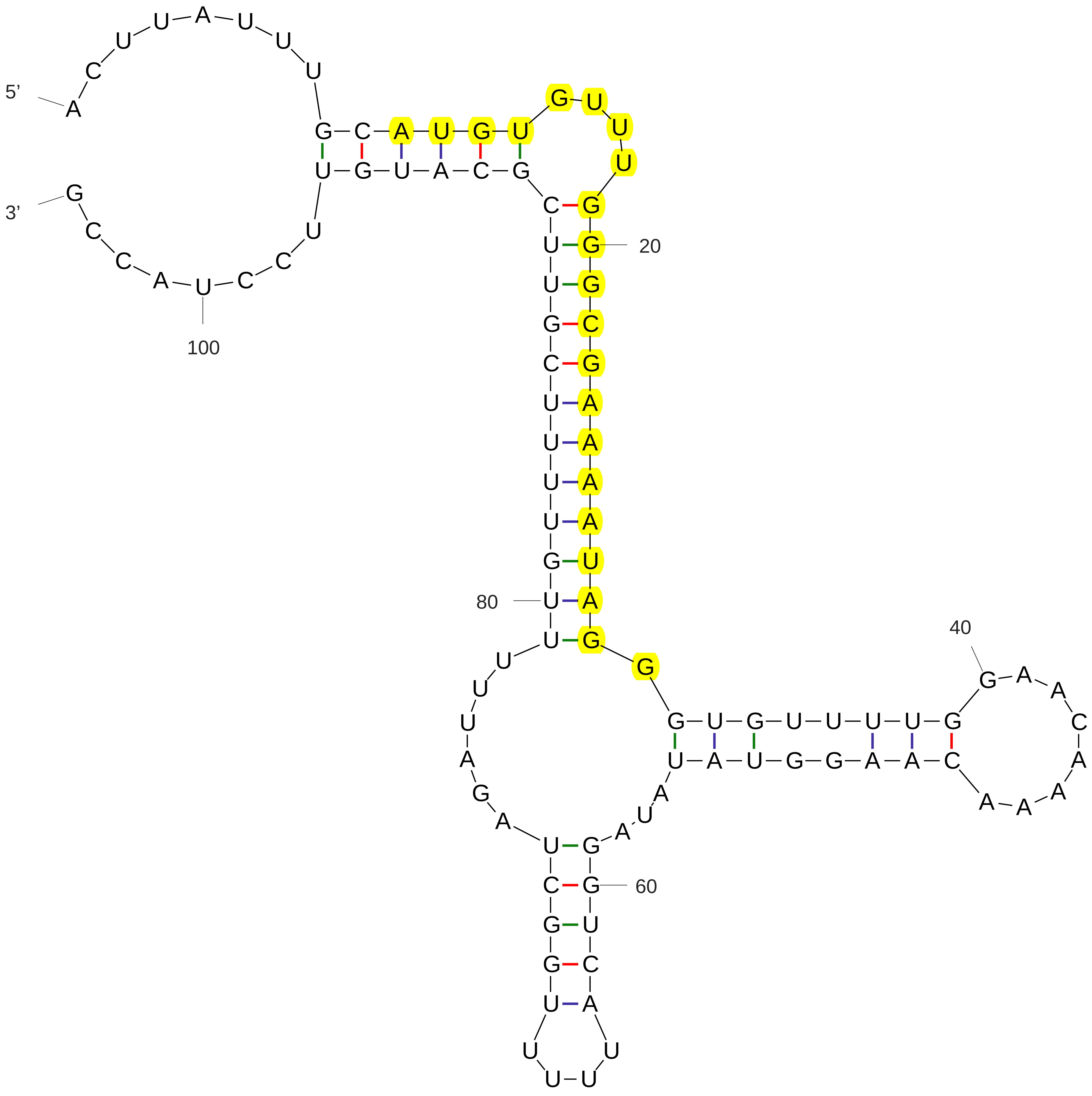

dG = -25.34 [Initially -24.60] Seu-miR27

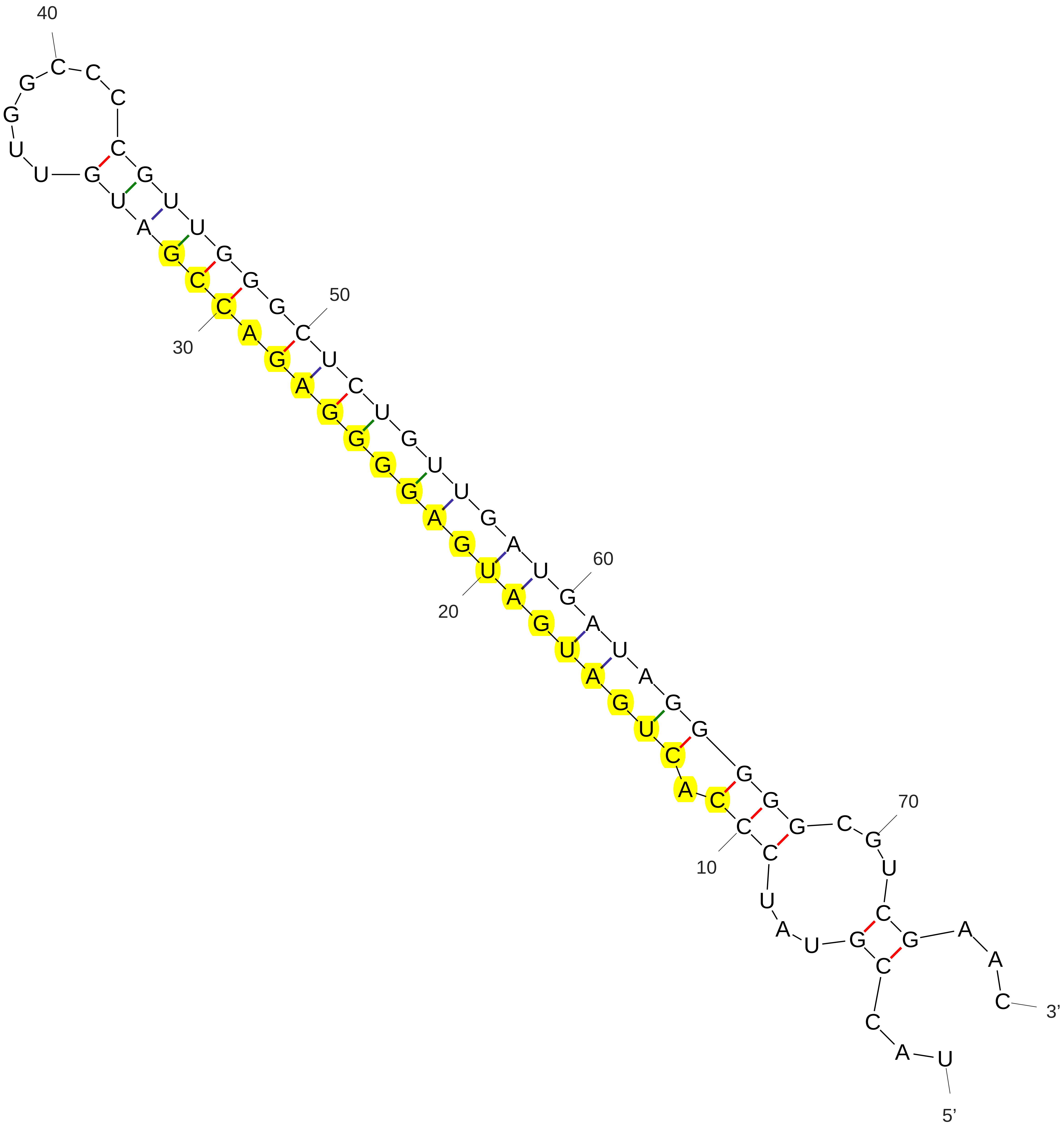

dG = -23.10 [Initially -23.10] Seu-miR28

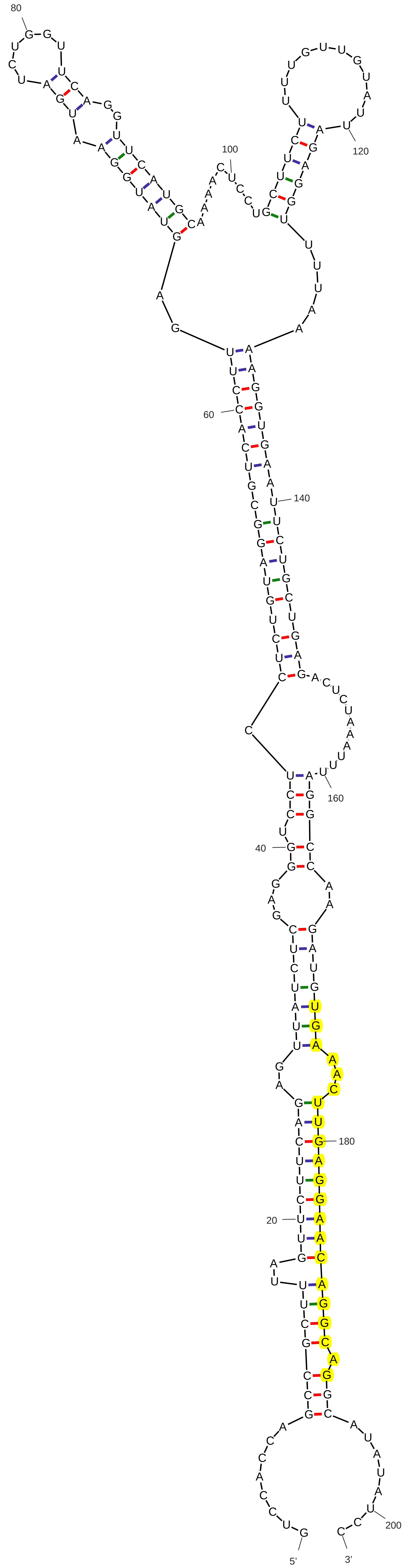

dG = -54.92 [Initially -58.20] Seu-miR29

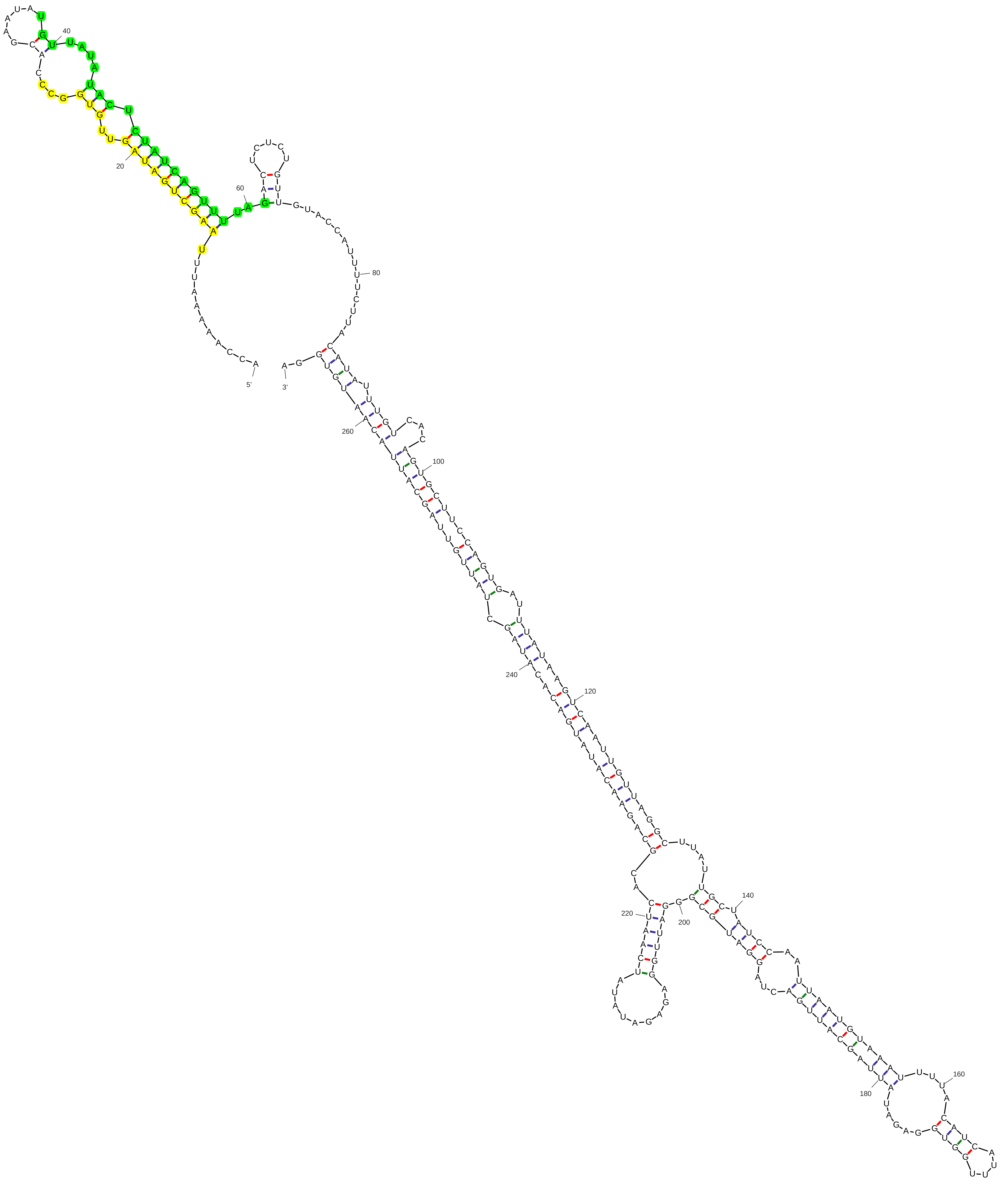

dG = -55.63 [Initially -57.80] Seu-miR30
