# Supplementary material for: High-throughput deep sequencing reveals that microRNAs play important roles in salt tolerance of euhalophyte Salicornia europaea
Source: BMC Plant Biol. 2015 Feb 26;15:63. doi: 10.1186/s12870-015-0451-3 (PMC4349674; doi:10.1186/s12870-015-0451-3)
Supplement: Additional file: 11. — The summary of salt-regulated miRNAs in S. europaea. [file 12870_2015_451_MOESM11_ESM.doc]

**Additional file 11. The summary of salt-responsive miRNAs in *S. europaea*.**

| miRNA | Seu | Tsa | Other species | Targets | Function description |
| --- | --- | --- | --- | --- | --- |
| miR156 |  |  | Zma Ath | SBP transcription factor | Vegetative phase change; root development |
| miR159 |  |  | Ath | retroelement pol polyprotein-like;  RNL (RNA LIGASE) | DNA integration;  Protein turnover |
| miR160 |  |  | Osa Pto | ARF transcription factor | Auxin signaling |
| miR164 |  |  | Zma | NAC transcription factor | Auxin signaling |
| miR166 |  |  | Sugarcane | ribonucleotide reductase small subunit;  ABC transporter | Cell cycle/division;  Transporter |
| miR165 |  |  | Pvi Ath |  |  |
| miR167 |  |  | Ath Zma | kinesin related protein;  DNAJ heat shock N-terminal domain-containing protein;  MEE50 (maternal effect embryo arrest 50) | Cell structure |
| miR168 | D |  | Ath Zma |  |  |
| miR169 |  |  | Osa | NF-YA transcription factor | root development, flowering time, nitrogen starvation responses, plant responses to drought and salt stress |
| miR171 |  |  | Ath | SCL transcription factor | shoot branching, primary root elongation, flower structure, leaf shape and patterning |
| miR172 |  |  | Cin | CER7 (ECERIFERUM 7);  UDP-sugar pyrophosphorylase  spliceosome protein-related | Transcription regulation;  Metabolism;  Transcription regulation |
| miR319 | D |  | Ath Pto Osa |  |  |
| miR393 |  |  | Pto | eIF3a | Protein turnover |
| miR394 | D |  | Gma Osa Pto Ath | F-box protein | Protein turnover |
| miR395 | D |  | Zma Gma Pto | OXA1 family protein,  NPY2,  GIP1  COP1-interacting protein-related | Protein turnover  Signaling  Transcription regulation  Vesicle transport |
| miR396 |  |  | Ath Zma Osa Pto | GRF transcription factor | leaf growth and development |
| miR398 |  |  | Ath Pto |  |  |
| miR399 |  |  | Pto | 2,3-biphosphoglycerate-independent phosphoglycerate mutase | Glygolysis, metabolism |
| miR408 |  |  | Pto Pvi |  |  |
| miR6300 |  |  |  |  |  |
| miR1 | S/R |  |  | | glyoxal oxidase | | --- | | RCa6 | | NBS-LRR type resistance protein | | Stress & disease response |
| miR2 | S/R |  |  | | leucine-rich repeat family protein / protein kinase family protein | | --- | | phosphoinositide 3-kinase | | signaling |
| miR4 | R |  |  | | anthraniloyal-CoA: methanol anthraniloyal transferase; | | --- | | AT4G37180 | | Metabolism  MYB transcription factor |
| miR5 | R |  |  | | auxin response factor 1 | | --- | | calcineurin-like phosphoesterase family protein | | Auxin signaling  signaling |
| miR8 | S/R |  |  | heat shock protein | Stress & disease response |
| miR9 | S/R |  |  |  |  |
| miR10a/b | S/R |  |  | | ATPAP29/PAP29 | | --- | | ankyrin repeat family protein | | transcription termination factor | | Metabolism  Stress & disease response  Transcription regulation |
| miR11 | S R |  |  | | ARPC1B (ACTIN-RELATED PROTEIN C1B); | | --- | | At5g59410; | | tetracycline transporter | | Cell structure  Vesicle transport  transporter |
| miR12 | S/R |  |  | | ABC transporter family protein | | --- | | LTR retrotransposon like protein | | Transporter  signaling |
| miR14 | S R |  |  | | gag non-LTR retrotransposase ; | | --- | | ASK21 (ARABIDOPSIS SKP1-LIKE 21) | | Transcription regulation  Protein turnover |
| miR18 | R |  |  | CXE carboxylesterase | metabolism |
| miR21 | S |  |  | | zinc-binding family protein | | --- | | membrane bound o-acyl transferase | | Unkonown  Lipid metabolism |

Seu, *Salicornia europaea*; Tsa, *Thellungiella salsuginea*; Ath, *Arabidopsis thaliana*; Zma, *Zea mays*; Osa, *Oryza sativa*; Pvi, *Panicum virgatum*, Pto, *Populus tomentosa*; Gma, *Glycine max*; Cin, *Caragana intermedia*

D: Dynamic expression patterns; ND: not determined or not change; S: shoot; R: root

Up-regulated down-regulated & some members were up-regulated, some were down-regulated
